# The transcriptomics of phenotypic nonspecificity in *Drosophila melanogaster*

**DOI:** 10.1093/g3journal/jkaf215

**Published:** 2025-09-15

**Authors:** Gabriella Sidhu, Anthony Percival-Smith

**Affiliations:** Department of Biology, The University of Western Ontario, London, Ontario N6A 5B7, Canada; Department of Biology, The University of Western Ontario, London, Ontario N6A 5B7, Canada

**Keywords:** doublesex, apterous, redundancy, transcription factor function, RNAseq, developmental genetics, 2-step nuclear search

## Abstract

Transcription factor (TF) function is redundant: TF phenotypes are frequently rescued by TFs not resident to the *TF* locus, a phenomenon termed phenotypic nonspecificity. Phenotypic nonspecificity in *Drosophila melanogaster* is not dependent on the DNA binding specificity of the TFs and generally due to genetic complementation. Two TF phenotypes (doublesex [dsx] and apterous [ap]) are rescued by multiple TFs. The rescue by resident TFs (DSX^F^ or AP) and the rescue and non-rescue by nonresident TFs of these 2 phenotypes were used to distinguish between 3 possible outcomes of the comparison of the TF-dependent mRNA accumulation in these 2 systems. First, the sets of TF-dependent mRNAs are independent and nonoverlapping; second, the sets of TF-dependent mRNAs are independent and overlapping; and third, the sets of TF-dependent mRNAs are constrained and have extensive overlap. The transcriptomes associated with rescue by resident TFs, and rescue and non-rescue by nonresident TFs, of the 2 TF phenotypes (dsx and ap) provided many examples of extensive overlap indicating regulation of constrained sets of genes. However, the strength of correlation of transcript accumulation observed between the resident and nonresident TFs was not a strong predictor for rescue of the phenotype by the nonresident TFs. The accumulation of a constrained set of mRNAs is discussed in relation to 3 potential explanations of phenotypic nonspecificity: limited specificity of TF function, the hypothetical assembly of TFs into wolfpacks, and chromatin accessibility.

## Introduction

The redundancy of transcription factor (TF) function results in the phenomenon of phenotypic nonspecificity where a phenotype due to the lack of expression of the resident TFa expressed from the *TFa* locus is rescued by expression of a nonresident TFb in place of TFa (Percival-Smith 2017; Percival-Smith et al. 2023). In *Drosophila melanogaster*, the rescuing resident and nonresident TFs often do not share similar DNA-binding sites nor belong to similar TF families. In most cases of phenotypic rescue, the rescue by the nonresident TFb is due to functional complementation rather than TFb being a downstream/epistatic component of the pathway. In addition, the rescue of the TF phenotypes by the nonresident TFs is differentially pleiotropic.

Phenotypic nonspecificity is a general phenomenon. It is detected at high frequencies with phenotypes induced by the ectopic expression of TFs in Drosophila such as wingless, eyeless, sex-comb-less, ectopic T1 beard formation, female abdominal pigmentation and arista to tarsus transformation phenotypes (Percival-Smith et al. 2005, 2023; Percival-Smith 2017), and in vertebrates for the re-establishment of pluripotency (Takahashi, and Yamanaka 2006; Maekawa et al. 2011). More importantly, phenotypic nonspecificity is detected in 8 of 9 Drosophila *TF* loci tested for rescue (Greig and Akam 1995; Hirth et al. 1998; Percival-Smith 2017; Percival-Smith et al. 2023). The estimated frequency of phenotypic nonspecificity of rescue of a TF phenotype in Drosophila is 1 in 10 to 20 TFs rescue. Phenotypic nonspecificity and phenotypic convergence share the conclusion that distinct sets of TFs expressed lead to the same phenotype (Konstantinides et al. 2018). Screens of TFs for the rescue of doublesex (dsx) female abdominal pigmentation pattern (88 TFs) and the rescue of the apterous (ap) wingless phenotype (52 TFs) identified multiple nonresident TFs that rescue these 2 phenotypes (Percival-Smith et al. 2023). Here, the transcriptomes associated with the expression of resident and nonresident TFs that rescue and do not rescue these 2 phenotypes are characterized.

The abdominal pigmentation pattern of Drosophila is a major external sexually dimorphic characteristic. The *dsx* gene encodes 2 functionally distinct TFs that determine secondary sexual characteristics: DSX^M^ determines male characteristics, and DSX^F^ determines female characteristics (Burtis and Baker 1989). Male abdominal pigmentation does not require DSX^M^ as a *dsx* null mutant has male-like pigmentation. For female abdominal pigmentation, DSX^F^ activates the expression of 2 functionally redundant TFs, Bric a´ Brac 1 (BAB1) and BAB2, which suppress the expression of Dopachrome-conversion enzyme encoded by the *yellow* (*y*) gene, and the beta alanyl dopamine hydrolase encoded by the *tan (t)* gene (Couderc et al. 2002; Han et al. 2002; True et al. 2005; Williams et al. 2008; Camino et al. 2015; Roeske et al. 2018). The male-like abdominal pigmentation of the dsx^null^ phenotype is rescued to female-like pigmentation by the expression of DSX^F^, BAB1, Antennapedia (ANTP), Buttonhead (BTD), Eyelesss (EY), Hunchback (HB), Knirps (KNI), Scalloped (SD), Sine ocullus (SO), or Oddskipped (ODD) but not by the expression of Forked head box subgroup O (FOXO) or Squeeze (SQZ) (Percival-Smith et al. 2023). The rescue by expression of BAB1 is expected as it functions downstream of DSX^F^; however, the other TFs that rescue have no normal requirement in abdominal pigmentation pattern. Analysis of the epistatic interactions between expression of DSX^F^, BAB1, ANTP, EY, or ODD and *dsx^null^* or *bab* alleles suggests that BAB1, ANTP, EY, and ODD all function after DSX^F^. As BAB1 and 2 bind *cis*-regulatory elements in *y* and likely *t* repressing expression, BAB1, ANTP, EY, and ODD likely function as interchangeable repressors of these 2 genes (Roeske et al. 2018). DSX^F^ activates BAB1 and BAB2 expression at around 72 h after pupa formation (APF), and the BAB dependent repression of a *y::GFP* transcriptional fusion gene is detected at 85 to 95 h APF, the P13-15 pupal stages (Williams et al. 2008; Roeske et al. 2018).

The spatially restricted expression of the Apterous (AP) TF during the second instar larval stage establishes the dorsal compartment of the wing imaginal disc and initiates a pathway required for wing formation (Cohen et al. 1992; Fisher et al. 2017). At the late first/early second instar larval stage, the first step in determination of the dorsal ventral axis of the wing imaginal disc is mutual negative interactions between Vein (VN) expressed on the future dorsal side and Wingless (WG) expressed on the future ventral side of the wing imaginal disc (Couso et al. 1993; Simcox et al. 1996). During the second instar larval stage, the VN signal activates the dorsal expression of AP that establishes the dorsal compartment. To initiate wing formation, AP activates the expression of Serrate (SER) and Fringe (FRN) (Diaz-Benjumea and Cohen 1993, 1995; Irvine and Wieschaus 1994). SER and FRN regulate the activity of the Notch pathway that then activates WG expression in the cells of the future wing margin (Klein and Arias 1998). WG expression along the wing margin is established by the late second/early third instar larval stage (Ng et al. 1996). Subsequently, WG and Decapentaplegic (DPP) promote the growth of the wing pouch, which during the pupal stages differentiates into the adult wing (Zecca and Struhl 2021). The wingless phenotype of an *ap^null^* mutant is rescued by the expression of AP, Caudal (CAD), Tramtrack (TTK), or Myb oncogene-like (MYB) but not Sisterless A (SISA). CAD and TTK are not required for wing development but loss of MYB does result in an altered wing suggesting a possible requirement in wing formation (Percival-Smith et al. 2023).

This paper characterizes the transcriptomes associated with the expression of the TFs BAB1, ANTP, EY, FOXO, ODD, or SQZ in a *dsx^null^* mutant background and the transcriptomes associated with the expression of the TFs AP, CAD, MYB, SISA, or TTK in an *ap^null^* mutant background. In these 2 systems (dsx and ap), the TF-dependent mRNA accumulations associated with the expression of various TFs were compared to distinguish between 3 potential outcomes. First, the rescuing resident and nonresident TFs regulate completely independent, nonoverlapping sets of genes; second, the TFs regulate independent overlapping sets of genes; or third, the TFs co-regulate a constrained set of genes. The TFs regulate expression of nonindependent, overlapping sets of mRNAs supporting the third outcome: the regulation of a constrained set of genes. The inclusion of nonresident TFs that do not rescue the phenotypes allowed comparison of the transcriptomic results of TFs that rescue with those that do not. The results are discussed in relation to previously proposed explanations for phenotypic nonspecificity of TF function (Percival-Smith 2018; Staller 2022; Percival-Smith et al. 2023).

## Materials and methods

### Drosophila husbandry

Flies were maintained at 24 °C and 60% humidity. The flies were reared in 20 ml vials containing corn meal media [1% (w/v) Drosophila grade agar, 6% (w/v) sucrose, 10% (w/v) cornmeal, 1.5% (w/v) yeast, and 0.375% (w/v) 2-methyl hydroxybenzoate]. For collection of early third instar larvae, flies laid eggs on apple juice plates [2.5% (w/v) Drosophila grade agar, 6% (w/v) sucrose, 50% apple juice, and 0.3% (w/v) 2-methyl hydroxybenzoate] smeared with a patch of yeast paste on the first day, and the progeny were allowed 1 d to hatch. After hatching, a layer of mashed corn meal media mixed with yeast paste was spread across the plate. On the fourth day, early third instar larvae (0 to 8 h old) were present for collection. For collection of P13-15 pupa, vials with wandering third instar larvae were inspected for white prepupa whose positions were marked on the vial with a permanent marker. The vials were incubated for 4 d and P13-15 pupa was identified based on described developmental markers (Ashburner 1989).

### The Drosophila crosses and genotype identification

The stocks used for the experiments in the *dsxGAL4/dsx^1^* null background were generated previously (Robinett et al. 2010; Percival-Smith et al. 2023; Supplementary Table 1). The following crosses were performed: *y w; dsx^1^/TM6B, Tb, P{walLy}* X *w; dsxGAL4/TM6B, Tb,* and *y w; P{UASX, w^+^}; dsx^1^/TM6B, Tb, P{walLy}* (where *X* could be *Antp, bab1, dsx^M^, ey, foxo*, or *sqz*) X *w; dsxGAL4/TM6B, Tb*, and *y w; P{UASdsx^F^, w^+^}, dsx^1^/TM6B, Tb, P{walLy}* X *w; dsxGAL4/TM6B, Tb*. The non-Tb pupae in the progeny were *dsxGAL4/dsx^1^* and expressing GAL4 alone or expressing GAL4 with a TF.

To identify wild-type male and female pupae, wandering third instar larvae were separated based on the size of the larval gonads: Male gonads are large causing a characteristic clearing (hole) of the opaque fat bodies. The male and female larvae were allowed to develop for 4 d in separate vials.

The stocks used for the experiments in the *apGAL4/ap^MIO1996-FLPSTOP.D^* null background were assembled using standard Drosophila crossing schemes (Supplementary Table 1). The following crosses were performed: *y w; apGAL4/CyO, P{UbiGFP, w^+^}PAD1* X *y w; ap^MIO1996-FLPSTOP.D^/CyO, P{UbiGFP, w^+^}PAD1*, and *y w; apGAL4/CyO, P{UbiGFP, w^+^}PAD1 X y w; ap^MIO1996-FLPSTOP.D^/CyO, P{UbiGFP, w^+^}PAD1; P{UASap, w^+^}*, and *y w; apGAL4/CyO, P{UbiGFP, w^+^}PAD1 X y w; ap^MIO1996-FLPSTOP.D^/CyO, P{UbiGFP, w^+^}PAD1; M{UASX, GW}ZH-86Fb* (where *X* is *cad*, *ttk*, or *sisA* fused to *3XHA tag*, or *myb*). The non-GFP-expressing larvae in the progeny were *apGAL4/ap^MIO1996-FLPSTOP.D^* expressing GAL4 alone or expressing GAL4 along with a TF.

### Tissue dissection

For dissection of P13-15 abdomens, pupae of the correct genotype were identified and dissected from the pupal case. In Drosophila Ringer's solution (3 mM CaCl_2_, 182 mM KCl, 46 mM NaCl, 10 mM Tris–HCl pH 7.2), the abdomen was dissected by grabbing the pupa between the thorax and abdomen with a pair of forceps and cutting off the abdomen by sliding a second pair of forceps along the first. The abdomens were placed in a microcentrifuge tube containing Ringer's solution on ice.

For dissection of late third instar larval wing imaginal discs, wandering third instar larvae were placed in Ringer's solution and screened for larvae that lacked GFP expression (no *CyO* balancer). The larvae were dissected using sharp forceps by grabbing the larvae in the middle with the first pair of forceps and pulling up a strip of dorsal cuticle with the second pair. The wing imaginal discs were identified and picked up with forceps and stored in a microcentrifuge tube containing Ringer's solution on ice.

Early third instar larvae (0 to 8 h after molting) were differentiated from late second instar larvae by the distinct, stage specific, structure of the anterior spiracles (Ashburner 1989). For dissection of early third instar larval tissue, larvae were placed in Ringer's solution and screened for lack of GFP expression. The larvae were dissected using sharp forceps by grabbing the larvae in the middle and pulling up a strip of dorsal cuticle to the mouth hooks and then cutting the larvae in half by sliding 1 pair of forceps over the other. The head skeleton including the attached gut and brain was removed with forceps leaving a patch of anterior cuticle that had the early third instar leg, wing, and haltere imaginal discs attached. The dissected patches were stored in a microcentrifuge tube containing Ringer's solution on ice.

### RNA extraction and RNAseq

The P13-15 abdomen samples had 5 to 8 abdomens per sample; the late third instar larval wing disc samples had 20 to 35 dissected wing discs per sample; and the early third instar larval anterior samples had 8 to 12 anterior cuticle patches per sample. Total RNA was extracted from the tissues using the RNeasy mini kit (QIAGEN). The number of biological replicates (≥5) was chosen based on published recommendations (Conesa et al. 2016) and was met for 20 sets of replicates with the exception of *n* = 4 for 4 sets of replicates (see Supplementary Table 2). The RNA samples were sequenced by Inomomic Inc. (Shenzhen, China) using DNBSEQ Eukaryotic Strand-specific Transcriptome Resequencing PE100 in 5 separate batches and PE150 for the final sixth batch. The number of clean forward reads and their batch and method are given in Supplementary Table 2.

### RNAseq data analyses

The reads were aligned to the BDGP6 assembly of the *D. melanogaster* genome using HISAT2 (version 2.2.1) (Kim et al. 2019). The count data were extracted with featureCounts (version 2.0.1) with the BDGP6.46.110 genome assembly (Liao et al. 2014; Hoskins et al. 2015) from the file of forward reads. Differential mRNA accumulation, often referred to as differentially expressed genes (DEG), was detected with DEseq2 (version 1.40.2) (Love et al. 2014) for a sample size of ≥5 (with 4 noted exceptions of *n* = 4), based on the recommendation of Li et al. (2022).

The base mean threshold for defining expressed genes from a DEseq2 analysis was chosen to be 1 rather than 10 because DEseq2 identified DEGs (*P*_adj_ < 0.05) between base means of 1 and 10, but not 0 and 1, and for the question posed, analysis of all DEGs needed to be included because the question pertains to the structure of the TF-dependent transcriptome and not the functional importance of individual genes identified. This is also the reason that no effect size threshold filter was applied to the data as well. Because the data analyzed were collected in 6 batches of sequencing, the principle components of variation in the DEseq2 analyses were determined (Supplementary Fig. 1). Generally for the dsx set of experiments, the PC2 (5% to 20% of the variation) showed separation of the control and TF samples and the PC1 (60% to 80% of the variation) showed the variation between replicates and the individual batches were spread out across PC1 in the dsx set of experiments suggesting a lack of a batch effect in batches 1, 2, and 3. The ANTP PCA was an exception showing a clustering of the TF from the control sloped across both PC1 and 2, and the ANTP analysis had the highest number of DEGs in the dsx set. The ap sets of experiments were unbalanced and had very high numbers of DEGs. For the L3 set of data, the TF and control were clearly separated along PC1, but due to the unbalanced origin of the samples, the effect of the TF could not be discerned from an effect of batch along PC1. The separation of the TF and control data occurred in 5 of the 6 PCAs of the E3 data along PC1 but to a lesser degree than L3; however, in the CAD analysis at E3, the data for the 3 batches were dispersed across PC1 (separation of the TF and control data was along PC2) suggesting a lack of a batch effect in batches 4, 5, and 6. Therefore, the data were analyzed in DEseq2 without taking a batch effect into consideration to reduce the degrees of freedom for the analyses. When plotting the log_2_ fold changes of 2 TF-dependent transcriptomes, the gene data with an NA (filtered out by DEseq2) for 1 of the 2 TFs were not included on the graphs.

Venn diagrams were generated with ggVennDiagram package version 1.5.4 (Gao et al. 2021). For the expected mean of the overlap of gene expression between *a* and *b* based on independent regulation, gene expression was calculated by multiplying the fractions, DEG*a*/total expressed genes*a* X DEG*b*/total expressed genes*b*, and multiplying the resulting fraction by the average of total expressed genes of *a* and *b*. The Monte Carlo simulation of the number of overlapping differentially expressed genes between 2 or more analyses was performed in 2 steps. First, a random number was chosen from a set of unique numbers equal to the total number of expressed genes determined in a DEseq2 analysis (base mean > 1) (Supplementary Method 1). This was repeated until the total number of unique numbers chosen was equal to the number of DEGs identified in the same DEseq2 analysis. These sets of numbers representing DEGs were generated for 2 or more independent DEseq2 analyses. In the next step, the union between the sets, which are the numbers shared between the sets, was counted. These Monte Carlo simulations were repeated 1,000 times generating a list of 1,000 simulated, shared DEG overlaps. The 95% confidence interval (CI) for each TF comparison was defined by the 25th numbers from both the lowest and highest numbers of DEGs generated in the 1,000 simulations.

For estimating the number of transcripts expressed from the endogenous *TF* loci and *UAS TF* constructs, Salmon (version 1.10.1) was used (Patro et al. 2017). Salmon was provided with the BDGP6 assembly of the *D. melanogaster* transcriptome supplemented with FASTA files of the UAS constructs used (Supplementary Data 1). The count data were then normalized to the sample with the highest total counts. In addition, because the relative lengths of transcripts affect the number of counts detected, the count data were divided by the length of the sequence used to search the reads in Salmon (counts/kb).

The virtual in situs were generated with the method described in Everetts et al. (2021). The script used to generate virtual in situs from a list of genes is included in the Supplementary Method 2. The virtual in situs were assigned to 1 of 11 expression categories by visual inspection.

### Statistical analysis

When DEseq2 performs an analysis of 2 sets of samples, the data are normalized; however, when the normalized count data of the same control samples of 2 DEseq2 analyses are compared, they differ. To avoid this variation in control count data when comparing multiple analyses, the featureCounts data were normalized to the one with the highest total counts in the dataset and the data plotted.

For the statistical analysis, the count data were treated as continuous data because all the counts of interest were high and above 0. The data were log transformed for some analyses to meet the criteria of an ordinary ANOVA, and multiple pairwise comparisons post hoc analyses were performed with Dunnett's test. For some analyses, a nonparametric analysis was required. These analyses were performed in the Prism statistics package (GraphPad).

### *Wingless* in situ hybridization

Late third instar larvae were dissected by grabbing the larva in the middle and ripping a strip of dorsal cuticle to the anterior tip and cutting the larvae in half. The tissue was fixed for 30 min in 5% formaldehyde in 1X phosphate buffed saline (PBS). The fixative was washed off with repeated washes of 1XPBS and the samples stored in 100% methanol. Thirty-five *wg* primers with the structure 21 *wg* bases followed by 30 Gs were synthesized. The 35 primers were mixed together at equal molarity and hybridized with a 3′ Alex488 labeled C15 primer at a molar ratio of 1 to 3.5, respectively. The tissue was hybridized at a concentration of 100 nM overnight at 37 °C and repeatedly washed as described in Calvo et al. (2021). The tissue was dispersed with a sharp hypodermic needle, mounted in Vectashield vibrance and the nuclei and *WG* imaged on a Nikon (Eclipse Ti2E) inverted microscope.

## Results

### Expression levels of the *UASTF* constructs

To assess whether the *UASTF* constructs were over- or under-expressed relative to the endogenous *TF* locus, the program Salmon was used to estimate the mRNA counts originating from the *UASTF* transgenes and the endogenous *dsx* and *ap* loci by adding the sequences of the *UASTF* transcripts to the Drosophila transcriptome (Patro et al. 2017). The transgenes, with 1 exception, were expressed higher relative to the endogenous loci: Transcripts from *UASdsx^F^* accumulate 1.8 times higher than endogenous *DSX^F^* transcripts, and transcripts from *UASap* accumulate 3.5 times higher in late third instar larval wing discs than transcripts from the endogenous *ap* locus (Table 1).

**Table 1.** Transcript abundance of expression from the UASTF transgenes relative to the endogenous locus.

mRNA	Pupa counts/kb	E3 counts/kb	L3 counts/kb	Expression ratio
Endogenous *DSX^F^*	170 ± 71	…	…	…
*UASDSX^F^*	310 ± 130	…	…	1.8
*UASBAB1*	640 ± 340	…	…	3.7
*UASANTP*	260 ± 200	…	…	1.5
*UASEY*	1,600 ± 350	…	…	9.2
*UASODD*	970 ± 710	…	…	5.6
*UASFOXO*	540 ± 210	…	…	3.1
*UASSQZ*	790 ± 80	…	…	4.6
Endogenous *AP*	…	150 ± 70	1,300 ± 530	…
*UASAP*	…	5,900 ± 2,800	4,500 ± 1,100	3.5
*UASCAD*	…	3,100 ± 1,200	4,100 ± 810	3.2
*UASMYB*	…	12,000 ± 4,700	15,000 ± 4,400	12
*UASTTK*	…	450 ± 100	1,100 ± 300	0.8
*UASSISA*	…	22,000 ± 5,800	26,000 ± 5,500	20

### DSX^F^ and BAB1-dependent mRNA accumulation

**Fig. 1.**
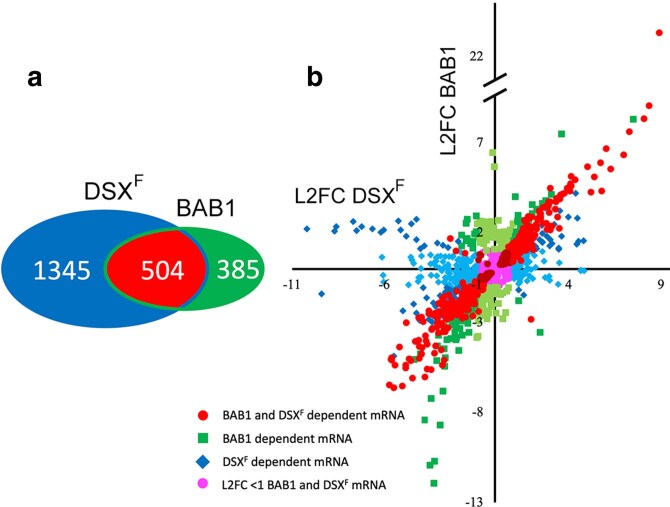
Analyses of the DSX^F^ and BAB1 dependent transcriptomes. a) A Venn diagram of the DEGs of the DSX^F^ and BAB1 dependent transcriptomes. b) Scatter plot of the log_2_ fold change (L2FC) of DSX^F^-dependent mRNA accumulation (blue diamond and red circular points) plotted on the *x* axis vs BAB1-dependent mRNA accumulation (green square and red circular points) plotted on the *y* axis. The circular points in red and burgundy are DEGs identified in both the DSX^F^ and BAB1 transcriptomes (*P*_adj_ < 0.05). The red circular points have a L2FC > 1 and <−1; the burgundy circular points have a L2FC < 1 and >−1. The diamond blue/light blue points are DEGs identified in the DSX^F^ transcriptome (*P*_adj_ < 0.05) but not the BAB1 transcriptome (*P*_adj_ > 0.05). The blue diamond points have a L2FC > 1 and <−1 with BAB1; light blue diamonds have a L2FC < 1 and >−1 with BAB1. The square green points are DEGs identified in the BAB1 transcriptome (*P*_adj_ < 0.05) and not the DSX^F^ transcriptome (*P*_adj_ < 0.05). The square green points have a L2FC > 1 and <−1 with DSX^F^; light green L2FC < 1; > −1 with DSX^F^. Magenta circular points were identified as DEGs in either the DSX^F^ or BAB1 transcriptomes with a L2FC of <1 and >−1 in both. A simplified legend is provided in the panel.

One expectation of DSX^F^ activating BAB expression for female pigmentation is that DSX^F^ expressed in a *dsx^null^* mutant should regulate a large set of genes that includes both DSX^F^ and BAB regulated genes; whereas, when BAB1 is expressed in a *dsx^null^* mutant, only the smaller subset of BAB1 regulated genes should be expressed. In a Venn diagram of the DSX^F^ and BAB1-dependent mRNAs, the set of BAB1-dependent mRNAs was smaller and 57% these mRNAs overlap with the DSX^F^-dependent mRNAs (base mean > 1; *P*_adj_ < 0.05) (Fig. 1a). Plotting the log_2_ fold change (L2FC) of the mRNAs displayed in the Venn diagram when DSX^F^ is expressed on the *x* axis and L2FC change of the mRNAs when BAB1 is expressed on the *y* axis showed a strong correlation of the TF-dependent mRNAs shared by these TFs (Pearson coefficient 0.94; Fig. 1b). Of the 504 mRNAs dependent on both DSX^F^ and BAB1 in the Venn diagram, only 7 showed alternate regulation (higher accumulation than the control with 1 TF; lower with the other TF). A linear regression of the red/burgundy points (R^2^ = 0.88, slope = 1.07 *y*-intercept = 0.06) supports the dependence of BAB1 regulated genes on the activation of BAB expression by DSX^F^; the slope of the L2FC is close to 1 indicating similar magnitudes of effect of BAB1 and DSX^F^, and 88% of the variance is explained by the relationship. A number of mRNAs (estimated at 80 to 150 mRNAs) that were not identified in the DSX^F^ set (green) and not identified in the BAB1 set (blue) showed similar fold changes in gene expression with both TFs, and this is due to not meeting the *P*_adj_ < 0.05 threshold with 1 of the TFs indicating that the 504 mRNAs dependent on both DSX^F^ and BAB1 are potentially an underrepresentation. Three hundred forty-three DSX^F^-dependent mRNAs (light blue points close to the *x* axis) showed a less than 2-fold change when BAB1 was expressed, as expected if there are DSX^F^ regulated genes that are not regulated by BAB1. However, there were 168 examples of BAB1-dependent mRNAs that showed a less than 2-fold change with DSX^F^ (light green points close to the *y* axis). Overall, the expectation of the set of BAB1-dependent mRNAs being nested in the set of DSX^F^-dependent mRNAs is generally supported.

### TF-dependent transcriptomes associated with rescue/non-rescue of female abdominal pigmentation

**Fig. 2.**
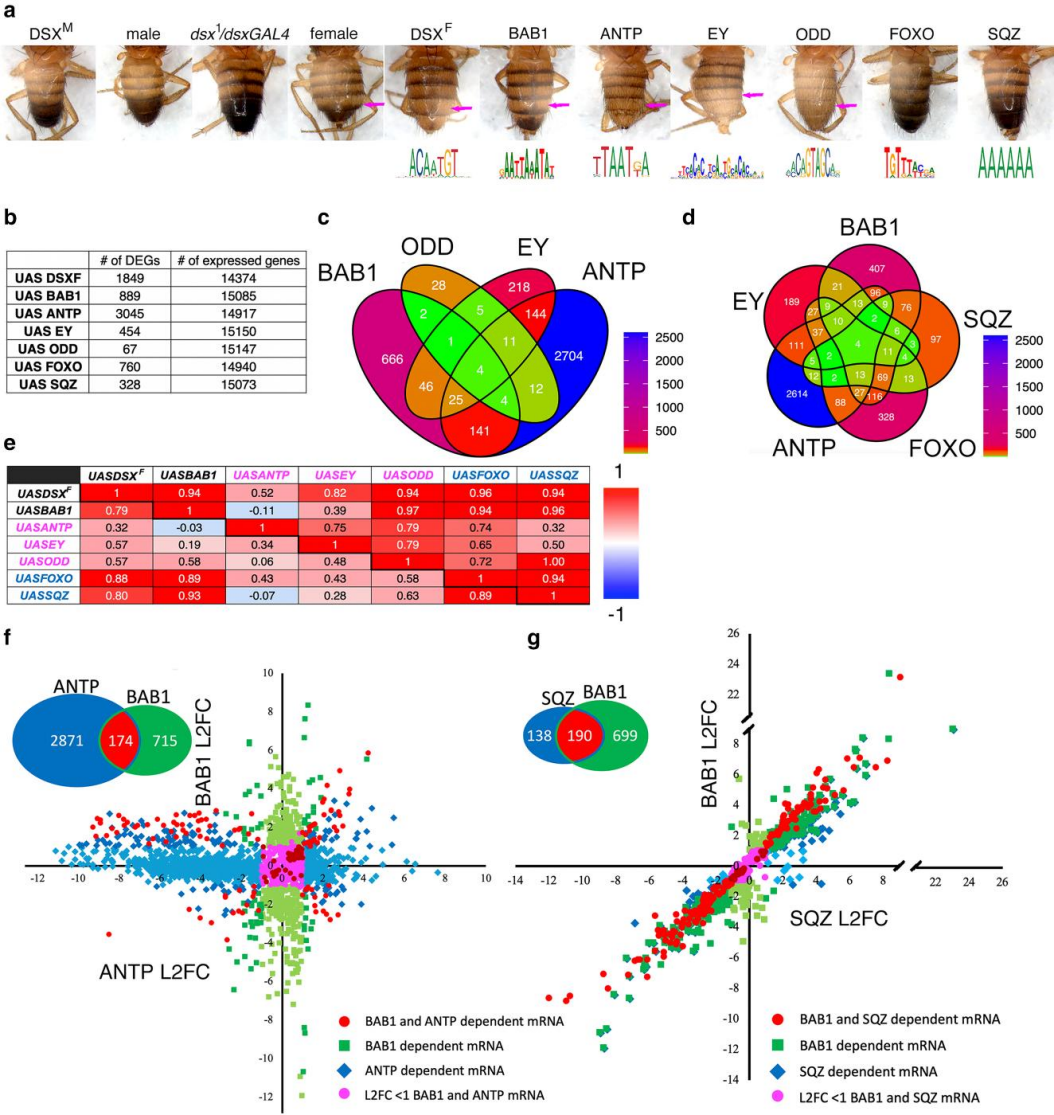
Analysis of the transcriptomes associated and not associated with rescue of female pigmentation in a *dsx^1^/dsxGAL4* null mutant background. a) Rescue of female pigmentation by expression of DSX^F^, BAB1, ANTP, EY, or ODD but not by FOXO or SQZ in a *dsx^1^/dsxGAL4* mutant background. Below the image of the abdominal phenotype associated with expression of a TF is a sequence logo of the DNA-binding site recognized by the TF (Vlieghe et al. 2006). The pink arrows indicate depigmentation of tergite 5. b) Table of the number of DEGs and expressed genes (base mean > 1) identified in the DEseq2 analyses. c) Venn diagram of the DEGs of the BAB1, ANTP, EY, and ODD transcriptomes. The color scale used to denote the number of DEGs is at the bottom right of the panel. d) Venn diagram of the DEGs of the BAB1, ANTP, EY, FOXO, and SQZ transcriptomes. The color scale used to denote the number of DEGs is at the bottom right of the panel. e) Table of Pearson's correlations between DEGs of various TF transcriptomes. The diagonal separates 2 sets of analyses. The Pearson's correlations below and left of the diagonal are for all the DEGs identified in 2 TF-dependent transcriptomes (all the points [red, burgundy, green, blue, magenta] in panels f) and g)). The Pearson's correlations above and right of the diagonal are for the DEGs shared between both TF-dependent transcriptomes (the red and burgundy circular points in panels f) and g)). f) Scatter plot of the log_2_ fold change (L2FC) of ANTP dependent mRNA accumulation plotted on the *x* axis vs BAB1-dependent mRNA accumulation plotted on the *y* axis. The inset is a Venn diagram of the data presented in the plot. The circular points in red and burgundy are DEGs identified in both the ANTP and BAB1 transcriptomes (*P*_adj_ < 0.05). The red circular points have a L2FC > 1 and <−1; the burgundy circular points have a L2FC < 1 and >−1. The diamond blue/light blue points are DEGs identified in the ANTP transcriptome (*P*_adj_ < 0.05) but not the BAB1 transcriptome (*P*_adj_ > 0.05). The diamond blue points have a L2FC > 1 and <−1 with BAB1; light blue diamonds have a L2FC < 1 and >−1 with BAB1. The square green points are DEGs identified in the BAB1 transcriptome (*P*_adj_ < 0.05) and not the ANTP transcriptome (*P*_adj_ > 0.05). The green square points have a L2FC > 1 and <−1 with ANTP; light green squares L2FC < 1; > −1. Magenta circular points are DEGs in either the ANTP or the BAB1 transcriptomes with a L2FC of <1 and >−1 in both. A simplified legend is provided in the panel. g) Scatter plot of the log_2_ fold change (L2FC) of SQZ dependent mRNA accumulation plotted on the *x* axis vs BAB1-dependent mRNA accumulation plotted on the *y* axis. The points are organized in the same way as panel f) with SQZ being substituted for ANTP. The inset is a Venn diagram of the data presented in the plot.

The TFs DSX^F^, BAB1, ANTP, EY, and ODD rescue female abdominal pigmentation of *dsx^null^* mutants and the TFs FOXO and SQZ do not rescue female pigmentation (Fig. 2a; Percival-Smith et al. 2023). All 7 TFs expressed in the *dsx^1^/dsxGAL4* background recognize distinct DNA-binding sites (Fig. 2a). From analysis of epistasis, the BAB1, ANTP, EY, and ODD TFs act after DSX^F^ and likely complement the loss of BAB expression in the *dsx^null^* mutant. The BAB1, ANTP, EY, ODD, FOXO, and SQZ dependent mRNAs expressed in the *dsx^null^* abdomens of P13-15 pupa were identified (Fig. 2b). Venn diagrams have an overlapping pattern of sharing between the sets of TF-dependent mRNAs with 4 mRNAs shared by BAB1, ANTP, EY, and ODD (Fig. 2c). A similar overlapping pattern of sharing was observed when SQZ and FOXO were added (Fig. 2d). Although this may suggest that the TFs potentially regulate independent sets of genes, the data were analyzed in 2 ways to assess further whether this pattern is truly stochastic and meets the criterion of independence.

First, the number of DEGs and total expressed mRNAs was used to repeatedly simulate the expected number of genes that would overlap to derive the 95% CI for the expected values. The observed values were then compared with the simulated data to assess whether they fall within the CI expected for stochastic behavior (Table 2). With the exception of 2 of 54 comparisons (BAB1 and ANTP; ANTP and SQZ), the observed number was higher than the simulated mean, the 52 observed numbers did not fall in the simulated 95% CI and the difference between the observed number and simulated mean have high z-scores indicating nonindependence (z-score = the difference between the observed number and the simulated mean/simulated standard deviation).

**Table 2.** Nonindependence of the overlap of transcriptomes in the rescue and non-rescue of dsx.

TF comparison	Shared genes	Expected	95% CI	Simulated	z-Score
BAB, ANTP	*174* ^a^	*180*	157 to 202	*179*	*0*
BAB, ODD	11	4	1 to 8	4	4
BAB, EY	76	27	17 to 37	27	10
BAB, SQZ	190	19	11 to 28	19	43
BAB, FOXO	259	45	33 to 57	45	36
ANTP, ODD	31	14	7 to 20	13	6
ANTP, EY	184	92	75 to 108	91	10
ANTP, SQZ	**49**	**67**	52 to 82	**66**	**−2**
ANTP, FOXO	183	155	135 to 177	155	3
ODD, EY	21	2	0 to 5	2	19
ODD, SQZ	7	1	0 to 4	1	6
ODD, FOXO	22	3	0 to 7	3	9
EY, SQZ	37	10	4 to 16	10	9
EY, FOXO	104	23	15 to 32	23	20
SQZ, FOXO	118	17	10 to 24	17	25
BAB, ANTP, ODD	8	1	0 to 3	0.8	7
BAB, ANTP, EY	29	5	1 to 11	5	12
BAB, ANTP, SQZ	28	4	1 to 8	4	12
BAB, ANTP, FOXO	54	9	4 to 15	9	15
BAB, ODD, EY	5	0.1	0 to 1	0.1	12
BAB, ODD, SQZ	3	0.1	0 to 1	0.1	10
BAB, ODD, FOXO	8	0.2	0 to 1	0.2	19
BAB, EY, SQZ	23	1	0 to 2	0.6	28
BAB, EY, FOXO	34	1	0 to 4	1	33
BAB, SQZ, FOXO	97	1	0 to 3	0.9	96
ANTP, ODD, EY	15	0.4	0 to 2	0.4	15
ANTP, ODD, SQZ	4	0.3	0 to 2	0.3	4
ANTP, ODD, FOXO	13	1	0 to 3	0.7	12
ANTP, EY, SQZ	13	2	0 to 5	2	11
ANTP, EY, FOXO	53	5	1 to 9	5	24
ANTP, SQZ, FOXO	21	3	0 to 8	3	9
ODD, EY, SQZ	4	0.04	0 to 1	0.05	20
ODD, EY, FOXO	13	0.1	0 to 1	0.1	43
ODD, SQZ, FOXO	6	0.1	0 to 1	0.1	20
EY, SQZ, FOXO	21	0.5	0 to 2	0.5	29
BAB, ODD, EY, ANTP	4	0.02	0 to 1	0.03	20
BAB, ODD, EY, FOXO	4	0.01	0 to 0	0.01	40
BAB, ODD, EY, SQZ	2	0.003	0 to 0	0.002	50
BAB, ODD, ANTP, FOXO	6	0.04	0 to 1	0.04	30
BAB, ODD, ANTP, SQZ	3	0.02	0 to 0	0.02	30
BAB, ODD, FOXO, SQZ	3	0.004	0 to 0	0.01	30
BAB, EY, ANTP, FOXO	14	0.3	0 to 2	0.3	27
BAB, EY, ANTP, SQZ	6	0.1	0 to 1	0.1	20
BAB, EY, FOXO, SQZ	15	0.03	0 to 1	0.03	75
BAB, ANTP, FOXO, SQZ	17	0.2	0 to 1	0.2	34
ODD, EY, ANTP, FOXO	10	0.02	0 to 0	0.02	100
ODD, EY, ANTP, SQZ	3	0.01	0 to 0	0.02	30
ODD, EY, FOXO, SQZ	4	0.002	0 to 0	0.002	100
ODD, ANTP, FOXO, SQZ	4	0.02	0 to 0	0.01	40
EY, ANTP, FOXO, SQZ	6	0.1	0 to 1	0.1	20
BAB, ODD, EY, ANTP, FOXO	3	0.001	0 to 0	0.001	100
BAB, ODD, EY, ANTP, SQZ	2	0.001	0 to 0	0.002	50
BAB, ODD, EY, FOXO, SQZ	2	0.0001	0 to 0	0.002	50
BAB, ODD, ANTP, FOXO, SQZ	3	0.001	0 to 0	0	3388
BAB, EY, ANTP, FOXO, SQZ	4	0.01	0 to 0	0.003	40
ODD, EY, ANTP, FOXO, SQZ	3	0.0005	0 to 0	0	6657
BAB, ODD, EY, ANTP, FOXO, SQZ	2	0.00003	0 to 0	0	75,274

CI, confidence interval.

^a^The italics indicates observed values that fall within the 95% confidence interval, and the bold indicates values that are lower than predicated.

Second, the overlap in a Venn diagram only represents a set of differential mRNA accumulation shared between 2 or more TFs and provides no information on the mode of regulation (positive or negative) and magnitude of the effect. Therefore, to characterize the transcriptomes further, the correlation between the effect of the TFs on log_2_ fold change (L2FC) of mRNA accumulation was assessed. Since in any analysis of differential mRNA accumulation about 50% are either up or downregulated by the action of a TF, there is no a priori reason to expect that 2 TFs have the same effect on the mode of regulation or the magnitude of regulation. The only other imposed factor on how mRNAs shared in a Venn diagram will behave in this analysis is that the shared mRNAs of both TFs (red/burgundy points) are required to have a strong enough effect on transcript accumulation to pass the *P*_adj_ of <0.05 threshold for being a DEG. For stochastic behavior, these considerations should result in little correlation between the L2FC of the 2 TFs, and the red/burgundy points, which are the shared mRNAs, should align in a diagonal cross with very few points along the *x* and *y* axes. This behavior was found for ANTP and BAB1 (Fig. 2f; the only pair in Table 2 that falls within the simulated 95% CI); however, for most comparisons there was a positive correlation (Fig. 2g). When the Pearson's correlation was calculated for all mRNAs dependent on either of 2 TFs (red/burgundy, green, magenta, and blue points), the correlations were generally good. To assess the magnitude of the response, linear regressions for the comparisons with strong Pearson's correlations (Fig. 2e) were performed and have slopes approaching 1 indicating a similar magnitude of effect of both TFs on accumulation of differentially expressed mRNA (Fig. 2g). The correlations were stronger when only the mRNAs that were dependent on both TFs were considered (red/burgundy points). Two interesting observations are: SQZ, which does not result in a female pattern of pigmentation, shows a strong correlation of transcript accumulation with BAB1 (Fig. 2g), but ANTP, which does result in female-like pigmentation, shows little correlation (Fig. 2f). Overall the correlations of transcript accumulation between TFs are not associated with phenotypic outcomes: FOXO and SQZ have the strongest correlations with BAB1, followed by ODD, then EY, and finally ANTP but FOXO and SQZ do not rescue and ODD, EY, and ANTP do. Both the simulation of stochastic behavior and analysis of correlations between transcriptomes suggest that the overlapping patterns of transcript accumulation are not stochastic and independent.

### Accumulation of mRNAs associated with sexually dimorphic pigmentation of the abdomen

**Fig. 3.**
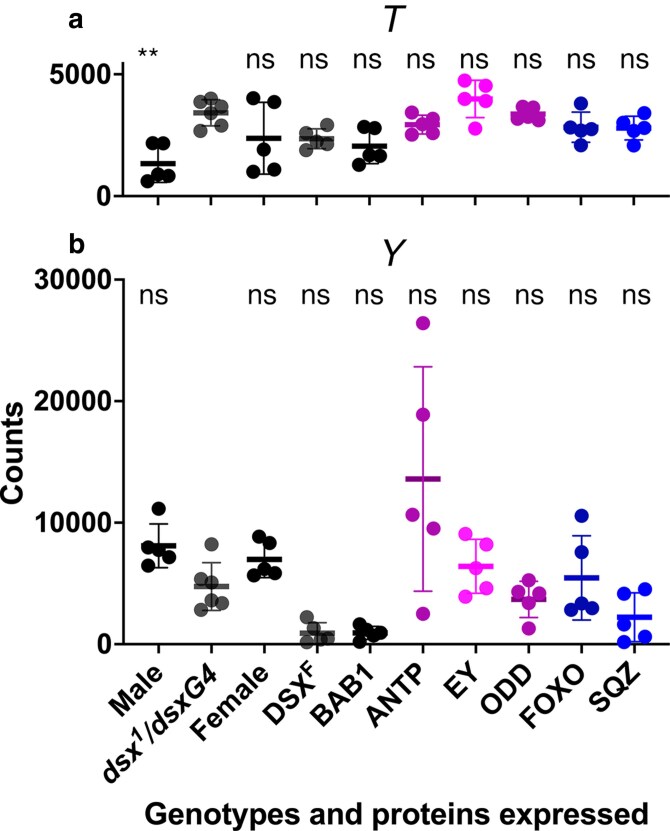
Accumulation of *T* and *Y* mRNA. Panels a) and b) are scatter plots of the normalized counts of *T* a) and *Y* b) mRNA accumulation for males, females, *dsx^1^/dsxGAL4* either not expressing a TF or expressing the TF indicated. At the top of the graphs is indicated the significance of any changes relative to *dsx^1^/dsxGAL4* in an ANOVA of ranks (*T* H(10) = 26, *P* < 0.002; *Y* H(10) = 34, *P* < 0.0001). ns, not significant; **P < 0.01.

The proteins encoded by the genes *y* and *t* are required for pigmentation of bristles and cuticle. Female cuticular pigmentation changes represent a small decrease in overall pigmentation of the abdomen. The accumulation of *Y* and *T* mRNAs overall (17/18) showed no changes (*P* > 0.05) in the levels of accumulation relative to the *dsx^1^/dsxGAL4* control. The only exception was a decrease of *T* accumulation in males which is unexpected because male abdomens are more pigmented than female abdomens (Fig. 3a). Although the requirement and regulation of *y* and *t* expression for cuticular pigmentation are well-characterized and established (Roeske et al. 2018), a potential role for Y and T in sexually dimorphic pigmentation would not have been identified in an RNAseq analysis of P13-15 male and female pupal abdomens, which is the time that Y and T are expressed and presumably required for abdominal pigmentation.

### Temporal characterization of AP-dependent mRNA accumulation

**Fig. 4.**
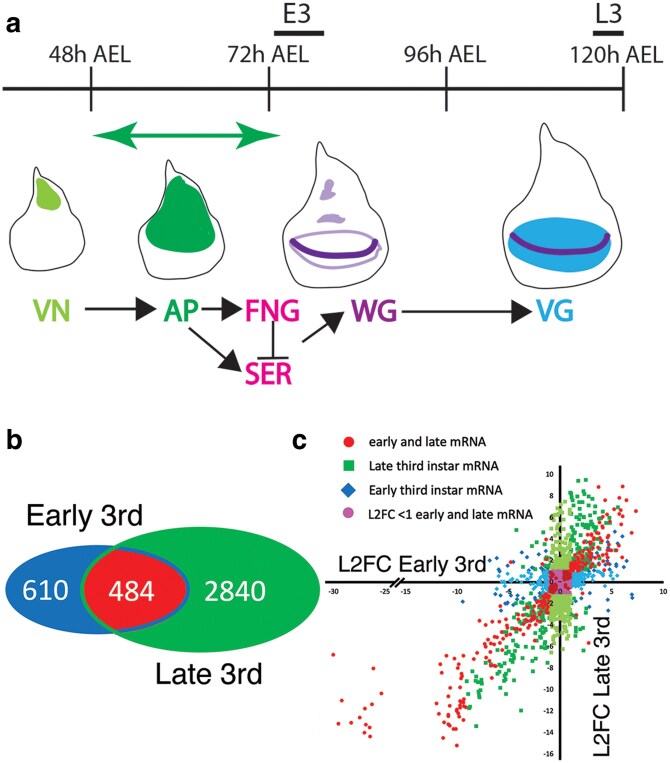
Temporal analysis of AP-dependent mRNA accumulation during larval development. a) Representation of the time course of wing imaginal disc development during the larval stages (AEL after egg-laying). Below the time are representations of developing and growing imaginal disc (the increase in size is not drawn to scale). During the first and early second instar larval stage, VN and WG determine the dorsal ventral axis, and VN activates the expression of AP (green). During the second instar and into the early third instar larval stage (E3) (the green double-headed arrow), AP is required for wing formation by activating FNG and SER expression (Diaz-Benjumea and Cohen 1993, 1995; Irvine and Wieschaus 1994; Fisher et al. 2017). FNG and SER through the regulation of Notch activity establish the expression of WG in the cells of the future wing margin (dark purple) by the early third instar larval stage. During the next 40 to 48 h till the late third instar larval stage (L3), the WG morphogen in cooperation with the DPP morphogen stimulates growth and differentiation of the wing pouch by regulating patterns of gene expression such as the expression of VG (blue). The bars above the timeline indicate the time intervals that tissue was dissected for RNA extraction. b) Venn diagrams of the AP-dependent transcriptomes identified by DEseq2 analysis at E3 and L3. c) Scatter plot of the log_2_ fold change (L2FC) of AP-dependent mRNA accumulation at E3 plotted on the *x* axis vs AP-dependent mRNA accumulation at L3 plotted on the *y* axis. The circular points in red and burgundy are DEGs identified in both the E3 and L3 transcriptomes as AP dependent (*P*_adj_ < 0.05). The red circular points have a L2FC > 1 and <−1; the burgundy circular points have a L2FC < 1 and >−1. The diamond blue/light blue points are DEGs identified in the E3 transcriptome (*P*_adj_ < 0.05) but not the L3 transcriptome (*P*_adj_ > 0.05). The blue diamond points have a L2FC > 1 and <−1 at L3; light blue diamonds have a L2FC < 1 and >−1 at L3. The square green points are DEGs identified in the L3 transcriptome (*P*_adj_ < 0.05) and not the E3 transcriptome (*P*_adj_ > 0.05). The green square points have a L2FC > 1 and <−1 at E3; light green squares L2FC < 1; > −1 at E3. Magenta circular points were identified as DEGs in either the E3 or L3 transcriptomes with a L2FC of <1 and >−1 in both. A simplified legend is provided.

During the second to early third instar larval stages, expression of AP initiates a pathway required for wing development (Fig. 4a; Fisher et al. 2017). At these early stages of imaginal disc development, AP regulates the expression of FNG and SER which result in the activation of WG expression along the future wing margin (Diaz-Benjumea and Cohen 1993, 1995; Irvine and Wieschaus 1994). During the following 48 h, WG patterns growth and development of the wing pouch. Larval wing imaginal discs are only large enough to dissect individually at the late third instar larval stage which is 2 d after the requirement of AP for initiation of wing development. To assess whether the AP-dependent transcriptome could be characterized closer to when AP is required, early third instar larvae were dissected by cutting them in half and removing the mouth hooks gut and brain to leave a patch of epidermis with leg, wing, and haltere discs attached. AP is expressed and required in both the wing and haltere imaginal discs (Cohen et al. 1992). Comparison of the AP-dependent transcriptomes of early third instar larvae and late third instar larvae showed significant overlap in AP-dependent mRNA accumulation in a Venn diagram (Fig. 4b) and showed a good correlation when the log_2_ fold changes of AP-dependent mRNA accumulation at early and late third instar larval stages were compared (Fig. 4c). Like the comparison of the DSX^F^ and BAB1 transcriptomes, the 484 genes that are shared in the Venn diagram are likely an underrepresentation as there are genes that were identified at 1 time point and show a similar log_2_ fold change in accumulation at the second time point but that did not meet the *P*_adj_ < 0.05 threshold at the second time point (blue and green points). The Pearson's correlation for the shared mRNAs is strong at 0.87; to assess the dependence of late expression on early expression, a linear regression of the shared mRNA was performed and has an R^2^ of 0.75. However, the slope of the regression was 0.7 which is due mainly to a set of 13 points that have very high negative fold changes at E3 (−25 to −30) pulling the slope down (removing these points, the slope is close to 1). These 13 genes all have high variability in the count data of the *ap^null^/apGAL4* control replicates which due to the methodology employed by DEseq2 to normalize the data and shrink log_2_ fold changes inflates the fold change (Love et al. 2014). Correlation between AP-dependent transcriptomes at early third and late third instar stages suggests that similar sets of AP-dependent transcripts are identified at these distinct temporal stages even though the early third instar larval tissues are not composed of only wing disc cells and that AP is also expressed in the haltere imaginal disc. Therefore, analysis of TF-dependent transcriptomes associated with phenotypic nonspecificity at early third instar larval stage, a time close to AP requirement, is feasible.

### TF-dependent transcriptomes associated with rescue/non-rescue of wing development

**Fig. 5.**
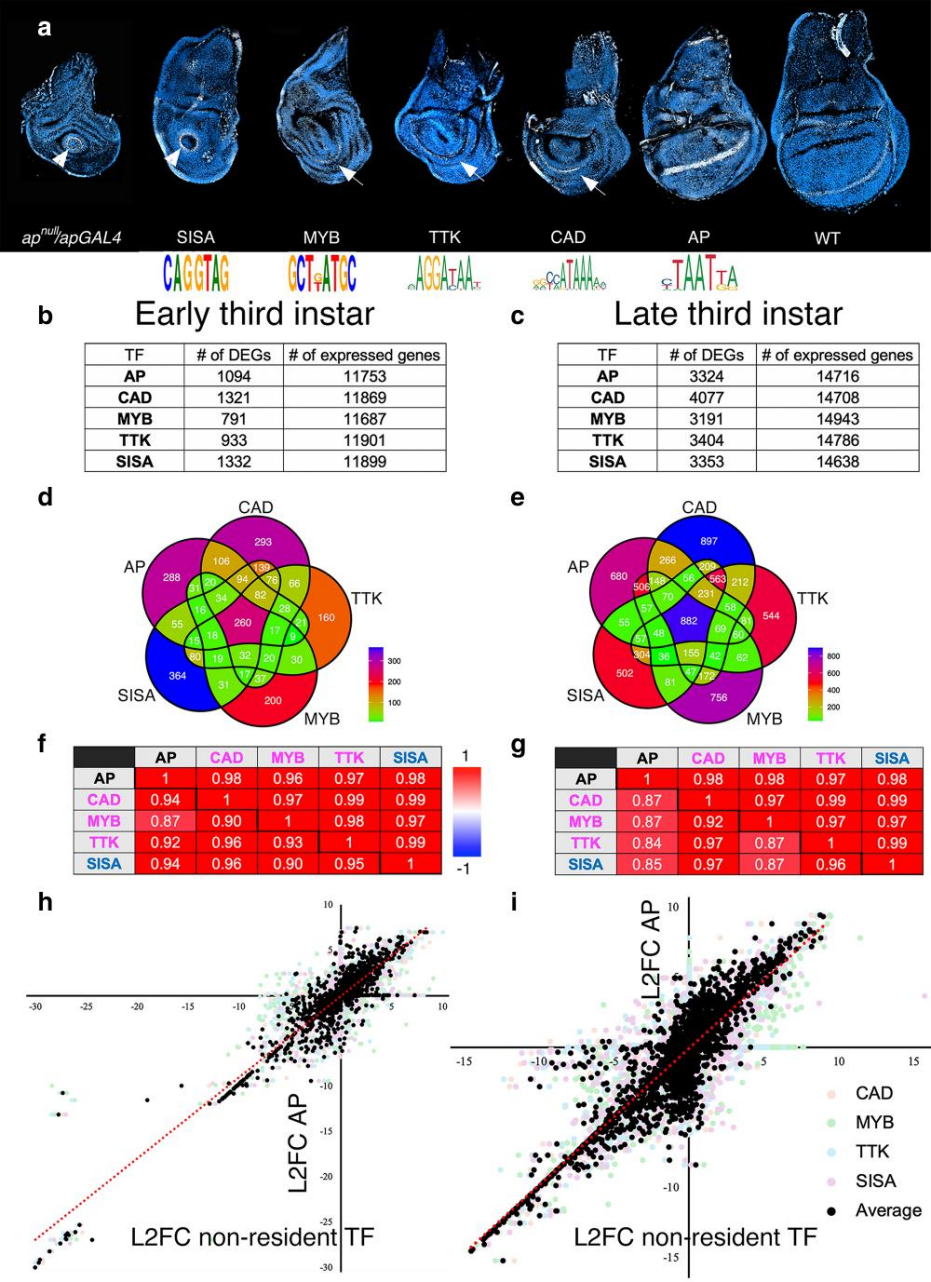
Analysis of transcriptomes associated and not associated with rescue of the wingless ap phenotype. a) *WG* in situ hybridization (white) on third instar imaginal discs with nuclei identified with DAPI (blue). The genotype or protein expressed is indicated below. The arrowheads point to a circle of *WG* expression and the arrows point to a crescent of *WG* expression potentially along the dorsal ventral boundary. Below the discs expressing particular TFs is the sequence logo of the AP, CAD, MYB, and TTK TFs, and the consensus binding sequence for the SISA TF (Erickson and Cline 1998; Vlieghe et al. 2006). b) Table of the number of DEGs and expressed genes (base mean > 1) identified in the DEseq2 analyses at the early third instar larval stage. c) Table of the number of DEGs and expressed genes (base mean > 1) identified in the DEseq2 analyses at the late third instar larval stage. d) Venn diagram of DEGs identified in *ap^null^/apGAL4* mutant background expressing AP, CAD, MYB, TTK, and SISA at the early third instar larval stage. e) Venn diagram of DEGs identified in *ap^null^/apGAL4* mutant background expressing AP, CAD, MYB, TTK, and SISA at the late third instar larval stage. The scale of the colors used in the Venn diagrams to denote the number of DEGs is at the bottom right of the 2 panels. f) Table of Pearson's correlations between DEGs of various TF transcriptomes at early third instar larval stage. The diagonal separates 2 sets of analyses. The Pearson's correlations below and left of the diagonal are for all the DEGs identified in either of the transcriptomes of the 2 TFs. The Pearson's correlations above and right of the diagonal are for the DEGs identified in both transcriptomes (*P*_adj_ < 0.05 for both). g) The same as f with the exception that they are the transcriptomes at the late third instar larval stage. h) Scatter plot of the log_2_ fold change (L2FC) of AP-dependent mRNA accumulation plotted on the *y* axis vs the L2FC for CAD, MYB, TTK, and SISA individually (lightly colored points, see legend), and the average of CAD, MYB, TTK, and SISA (black points) dependent mRNA accumulation plotted on the *x* axis for the transcriptomes at early third instar larval stage. i) Same as h except the transcriptomes are at late third instar larval stage. The dotted red line in h and i are the trend-lines of linear regressions. The legend for h and i is shown at the bottom right.

The ap wingless phenotype is partially rescued by expression of CAD, MYB, or TTK, but not SISA (Percival-Smith et al. 2023). The TFs AP, CAD, MYB, and TTK recognize distinct DNA-binding sites (Fig. 5a). SISA, from the sequence analysis of *Sex lethal* early promoter, is proposed to bind a consensus sequence (Fig. 1a) but this sequence has not been confirmed in a protein DNA binding assay with SISA as a DNA-binding site (Erickson and Cline 1998). To characterize the rescue further, wing imaginal discs from *ap* mutant third instar larvae expressing AP, CAD, MYB, TTK, or SISA were dissected and *WG* expression assessed. The wing discs expressing GAL4 alone or with SISA have a circular ring of WG expression in the reduced wing pouch (Fig. 5a). In the wing discs expressing CAD, TTK, or MYB, *WG* is expressed in a crescent shape that is potentially along the dorsal ventral boundary of the disc (Fig. 5a). The expression of AP rescues the expression of *WG* in wing margin cells.

The TF-dependent transcriptomes were identified at both the early and late third instar larval stages (Fig. 5, b and c). The Venn diagram comparison of the TF-dependent transcriptomes (AP, CAD, MYB, TTK, and SISA) at both larval stages showed extensive overlap of TF-dependent mRNA accumulation: At early third instar larval stage, accumulation of 1,373 mRNAs was dependent on 2 or more TFs and 1,305 were dependent on a single TF; at late third instar larval stage, accumulation of 3,467 mRNAs was dependent on 2 or more TFs and 3,379 were dependent on a single TF (Fig. 5, d and e). Using the number of DEGs and the total number of expressed mRNAs to simulate the expected values and determine a 95% CI based on stochastic behavior, not a single observed value was low enough to fall into the CI and all z-scores were high (Table 3). When all the L2FCs of the mRNAs that changed in 1 of 2 TF analyses were plotted on either the *x* or *y* axis, the correlations were high. The correlation increased when only those shared between the 2 TFs were compared (Fig. 5, f and g). To visualize the data for all TFs on 1 plot, the average of the fold changes observed with CAD, MYB, TTK, and SISA for 2,678 mRNAs at E3 and 6,846 mRNAs at L3 were plotted against the fold change observed with AP (Fig. 5, h and i; black points). This approach was possible because first the correlations were very high at both larval stages. Second, at the early third instar stage, 90% of the mRNAs differentially expressed with the 4 nonresident TFs had all 4 L2FC values either all positive or all negative and this increased to 96% at the late third instar larval stage. To indicate the variance in the data, the L2FC of the individual nonresident TFs were plotted as lightly colored transparent points below the averages; a small proportion of lightly colored points are found outside the cloud of average points (Fig. 5, h and i lightly colored points). Four separate plots of the data are presented in Supplementary Figs. 2 and 3. The slope of a linear regression was close to 1 indicating that the magnitude of the effect on mRNA accumulation was similar with all 5 TFs. One striking set of points is the near perfect line of points in the bottom left quadrant of the graphs comprised of genes repressed by all TFs (Fig. 5, h and i; Supplementary Figs. 2 and 3). These points all have counts in the control replicates and very low or 0 counts with any of the TFs expressed. This pattern of counts in the data, in combination with the similarity between overall transcriptomes, results in the very similar L2FC with different TFs producing a straight line of points (Supplementary Figs. 2 and 3). The transcriptomes associated with the resident AP TF and these 4 nonresident TFs show strong co-expression suggesting the regulation of a constrained set of genes. The correlation was strong irrespective of whether the ap phenotype was rescued or not.

**Table 3.** Nonindependence of the overlap of transcriptomes in the rescue and non-rescue of ap.

TF comparison	Early third instar					Late third instar				
	Shared genes	Expected	95% CI	Simulated	z-Score	Shared genes	Expected	95% CI	Simulated	z-Score
AP, MYB	405	121	101 to 142	121	28	1,840	715	669 to 750	710	54
AP, CAD	641	74	60 to 90	74	52	1,780	920	874 to 963	921	37
AP, TTK	450	86	70 to 102	86	46	1,486	766	725 to 806	765	34
AP, SISA	574	122	103 to 143	122	44	1,456	759	713 to 796	756	33
MYB, CAD	437	63	72 to 104	62	49	1,585	878	829 to 916	872	32
MYB, TTK	405	63	48 to 76	62	49	1,354	731	685 to 767	727	30
MYB, SISA	427	89	72 to 105	89	42	1,376	723	672 to 758	716	30
CAD, TTK	581	104	87 to 121	103	53	2,212	941	895 to 981	938	55
CAD, SISA	734	148	127 to 167	148	53	2,213	932	883 to 975	930	56
TTK, SISA	582	104	86 to 122	105	53	2,276	776	730 to 813	773	72
AP, MYB, CAD	331	8	3 to 14	8	108	1,169	198	171 to 223	196	75
AP, MYB, TTK	304	10	2 to 11	6	149	1,059	165	142 to 186	163	75
AP, MYB, SISA	328	8	3 to 14	8	107	1,057	163	138 to 186	162	75
AP, CAD, TTK	387	10	4 to 16	10	126	1,240	212	186 to 238	212	73
AP, CAD, SISA	470	14	7 to 21	14	114	1,239	210	183 to 235	210	74
AP, TTK, SISA	375	10	4 to 16	10	122	1,218	175	150 to 197	174	87
MYB, CAD, TTK	329	7	2 to 12	7	107	1,148	202	173 to 227	200	68
MYB, CAD, SISA	343	10	4 to 17	10	111	1,154	200	173 to 225	199	73
MYB, TTK, SISA	329	7	2 to 12	7	107	1,121	167	142 to 188	165	80
CAD, TTK, SISA	450	12	6 to 18	12	146	1,831	215	188 to 243	214	116
AP, MYB, CAD, TTK	277	1	0 to 2	1	276	951	45	33 to 60	46	129
AP, MYB, CAD, SISA	294	1	0 to 3	1	293	952	45	34 to 58	45	151
AP, MYB, TTK, SISA	278	1	0 to 3	1	277	930	38	25 to 49	37	149
AP, CAD, TTK, SISA	342	1	0 to 4	1	341	1,113	49	35 to 62	48	152
MYB, CAD, TTK, SISA	292	1	0 to 3	1	291	1,037	46	32 to 59	46	142
AP, CAD, MYB, TTK, SISA	260	0.1	0 to 1	0.1	867	882	10	5 to 16	10	291

CI, confidence interval.

### Genes potentially directly regulated by AP

**Fig. 6.**
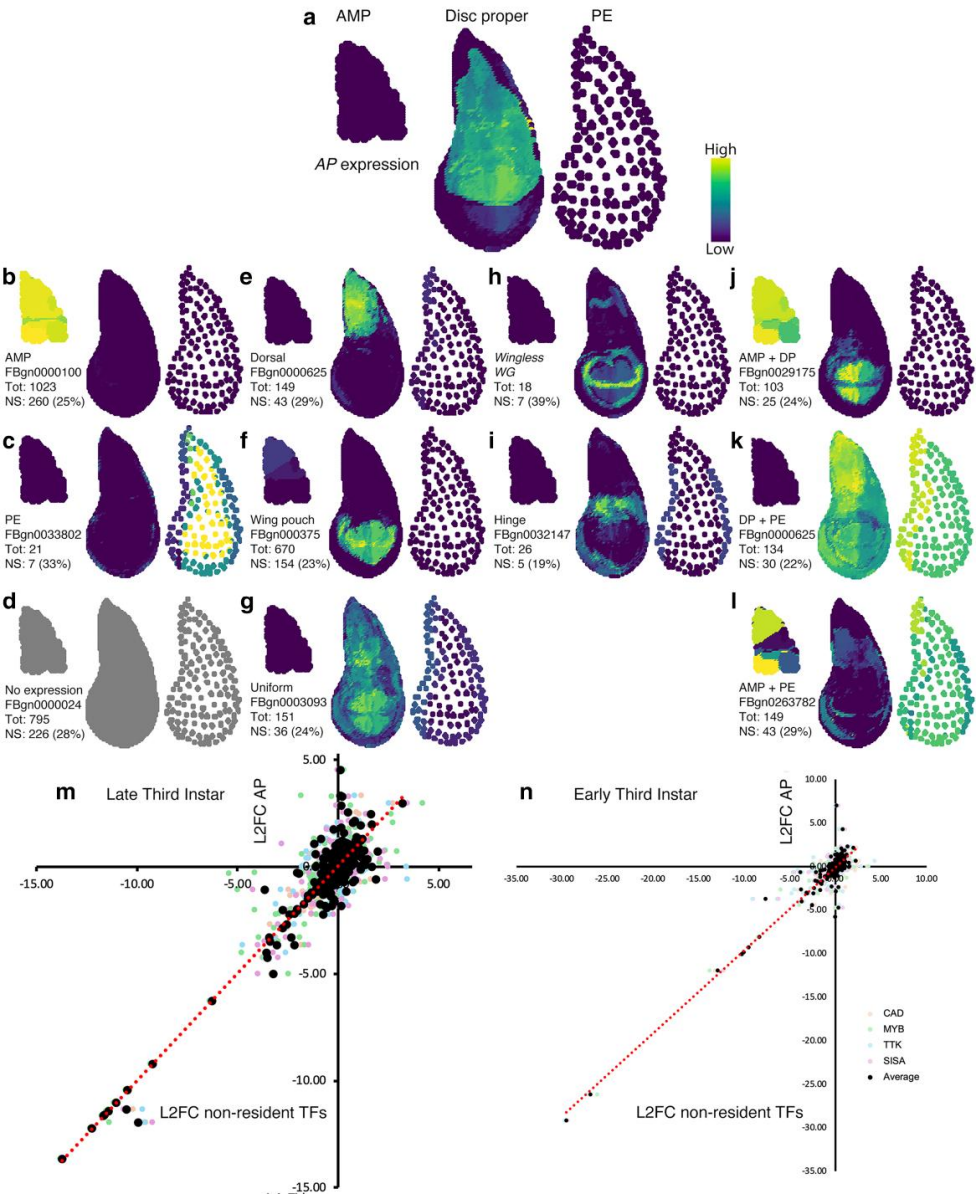
Virtual in situ analysis of AP-dependent mRNA accumulation and the change of AP-dependent mRNA accumulation in AP-expressing cells. a) A virtual in situ of AP expression in the dorsal compartment of the disc proper. The expression in all virtual in situs is shown in the mesodermal adult muscle precursor cells (AMP), the epithelial disc proper cells, and the peripodial envelope cells (PE). (b to l) In situs for all 3,324 AP-dependent mRNAs sorted into 11 expression categories of which an exemplar is shown. The total (Tot) number of mRNAs in each nonoverlapping category is given. The number of mRNAs shared by AP, CAD, MYB, and TTK is given below the total (Phenotypically nonspecific: NS) for each category with a percent of the total given. m) Scatter plot of the log_2_ fold change (L2FC) of AP-dependent mRNA accumulation plotted on the *x* axis vs the average of CAD, MYB, TTK, and SISA dependent mRNA accumulation plotted on the *y* axis for the transcriptomes at late third instar larval stage for the 294 genes that are expressed in the dorsal compartment (black circles). Along with the average are plotted the AP vs the 4 individual TFs (CAD light orange, MYB light green, TTK light blue, and SISA light red) to display the variance in the data. n) Scatter plot is as shown in m but with data from the early third instar larval stage (base mean > 1). The dotted red line in m and n is the trend-line of a linear regression. The legend for m and n is shown at the bottom right.

At the late third instar larval stage, the total number of mRNAs showing a highly correlated change in accumulation is very high at close to 40% of the expressed mRNAs. Therefore, it is possible that AP and the nonresident TFs (CAD, MYB, TTK, and SISA) trip a common switch that initiates a massive common response that obscures the specific and diverse regulation of genes by these 5 different TFs within the dorsal AP-expressing cells (Fig. 6a). Our approach to address this issue was to ask whether mRNAs expressed in the dorsal epithelium of the wing imaginal disc had been identified as AP-dependent. The virtual in situ tool developed by Everetts et al. (2021) was used to classify all the AP-dependent mRNAs into 11 classes of expression patterns (Fig. 6, b to l). No obvious preference was observed in how the 951 mRNAs shared by AP, CAD, MYB, and TTK partitioned between the 11 expression categories; each category has about 25% of these mRNAs. The 149 AP-dependent mRNAs expressed in dorsal cells and 147 additional mRNAs identified as nonresident TF-dependent and expressed in dorsal cells were plotted AP vs the average of the 4 nonresident TFs (Fig. 6, m and n; black circles) and AP vs the individual TFs CAD, MYB, TTK, and SISA (Fig. 6, m and n, the lightly colored points). The average was chosen again for the same reasons as before for presenting a single plot, and the graphs of the L2FCs for the individual nonresident TFs are presented in Supplementary Figs. 4 and 5. The correlation between these mRNA expressed in the AP-expressing cells, and therefore, potentially directly regulated by AP was very high at both the late and early third instar larval stages. Although not all of these 296 (4% of the total DEGs) genes may be directly regulated by AP, there was still a strong correlation of expression observed allowing the average of the 4 nonresident TFs to be plotted against AP, which would not have been the case if diverse gene regulation patterns were initiated in dorsal cells by the different TFs.

### Expression of genes required for wing development

**Fig. 7.**
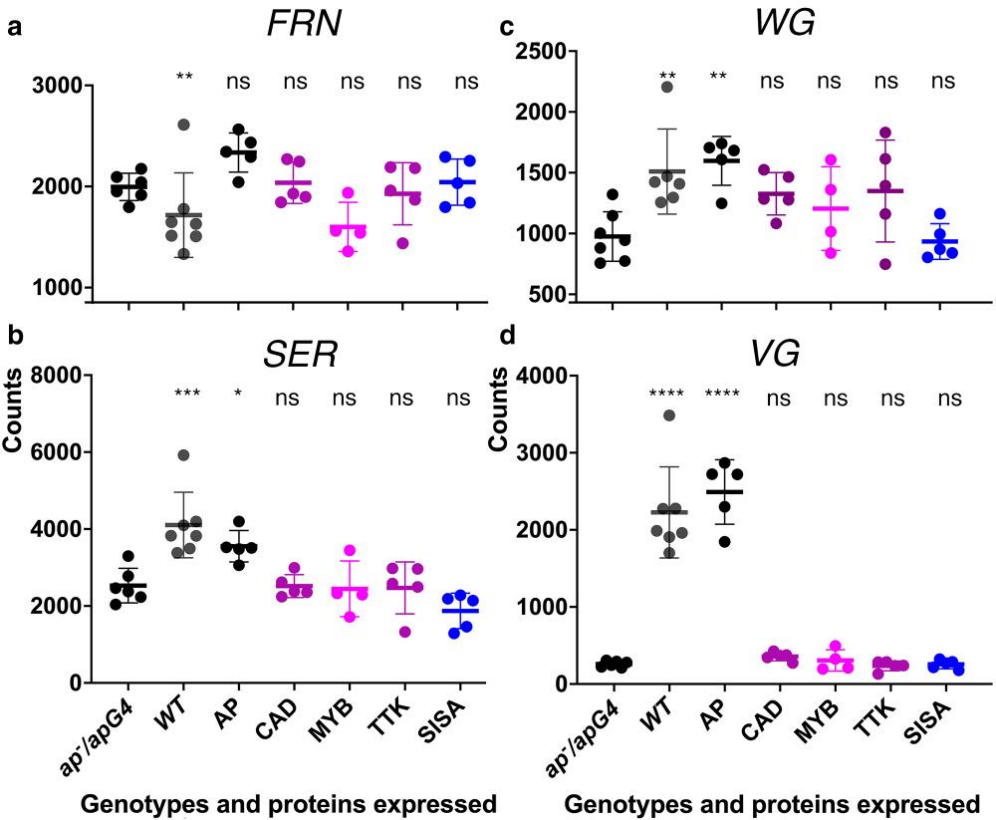
Accumulation of *FRN*, *SER*, *WG*, and *VG* mRNA. Panels a) to d) are scatter plots of the normalized counts of *FRN* a), *SER* b), *WG* c), and *VG* d) mRNA accumulation in *ap^null^/apGAL4* either not expressing a TF or expressing the TF indicated or WT. At the top of the graph is indicated the significance of any changes relative to *ap^null^/apGAL4* in an ordinary ANOVA (*FRN* F_7,33_ = 6, *P* < 0.0002; *SER* F_7,33_ = 9, *P* < 0.0001; *WG* F_7,33_ = 5, *P* < 0.001; *VG* F_7,33_ = 64, *P* < 0.0001). ns, not significant; *P < 0.05; **P < 0.01; ***P < 0.001; ****P < 0.0001.

The WG developmental pathway required for wing development is initiated by the AP TF. AP regulates the expression of 2 genes, *frn* and *Ser*, whose products regulate Notch receptor activity required for establishment of WG secretion from the cells of the future wing margin. The WG morphogen activates the expression of the *vg* gene. The components of this pathway were identified by the phenotype of mutations; therefore, would RNAseq analysis at late third instar larval stage have identified this developmental pathway? The *FNG* mRNA is present in all samples but is unexpectedly lower in wild type imaginal wing discs (Fig. 7a). The *SER* mRNA does increase in abundance in WT and AP rescued wing discs (Fig. 7b). The *WG* mRNA does increase in abundance in WT, and AP rescued wing imaginal discs as defined by having a *P* < 0.05 (Fig. 7c), but it should also be noted that the magnitude of *WG* expression was higher in WT, AP, CAD, TTK, and MYB than in both *ap^null^/apGAL4* and SISA. However, these 3 mRNAs do not meet the threshold of a 2-fold change set in most RNAseq analyses for the differential expression effect size and would have been overlooked. This small change is expected from prior expression analyses: At the late third instar larval stage, *FRN* mRNA is uniformly expressed across the wing imaginal disc; expression of SER protein is increased in the AP domain but not restricted to the AP domain of early third instar larval imaginal discs; in wing imaginal discs, WG expression is not restricted only to the cells of the future wing margin (Irvine and Wieschaus 1994; Diaz-Benjumea and Cohen 1995; Calvo et al. 2021). *VG* mRNA strongly accumulates in WT and AP rescued wing imaginal discs, but not in CAD, MYB, or TTK rescued wing imaginal discs (Fig. 7d). Therefore, only *VG* would have been identified in an RNAseq analysis.

## Discussion

### Transcriptomes associated with phenotypic nonspecificity

The goal that initiated this study was distinguishing between 3 possible expectations for the transcriptomes associated with phenotypic nonspecificity. The transcriptomes associated with TFs that both rescued and did not rescue the dsx and ap phenotypes were overlapping sets of mRNA accumulation that were not stochastic/independent. The lack of independence was observed as a higher than expected overlap between transcriptomes and a high correlation of the responses to expression of different TFs. For both the dsx and ap systems, the regulation of a constrained set of genes is supported. However, these correlations in response to expression of different TFs are not good predictors of phenotypic nonspecificity. The ANTP transcriptome, which shows low correlation with BAB1 transcriptome, does rescue but the SQZ transcriptome, which correlates well with the BAB1 transcriptome, does not rescue. For the analyses of the ap phenotype, the SISA transcriptome, which does not rescue the ap phenotype, does have a high correlation with the AP transcriptome. The high degree of correlation between the TF-dependent transcriptomes would have led to speculation that some of the nonresident TFs may be able to rescue the dsx or ap phenotype, if this transcriptomic study had been performed without knowledge of phenotypic nonspecificity.

### Proposed mechanisms for phenotypic nonspecificity

The systematic analyses of TF function and real-time visualization of transcription have challenged many common assumptions of the role of eukaryotic TFs in regulating the rate of transcription initiation. These 4 assumptions are: (i) a primary requirement for the DNA-binding domain (DBD) in occupying particular sites in the genome; (ii) clear modularity/separation of domains required for occupancy, transcriptional regulation, and pleiotropy; (iii) mutual cooperativity in occupancy; and (iv) assembly of TFs and RNA polymerase results in a constant rate of transcriptional initiation. A large-scale analysis of the requirement of the DBD of 48 yeast TFs for genome occupancy concluded that the mechanism of occupancy of TFs can be anywhere on a spectrum from complete DBD dependence to complete DBD independence (Brodsky et al. 2020; Kumar et al. 2023). The DBD dependence/independence of the Drosophila Fushi tarazu (FTZ) TF is differentially pleiotropic with FTZ being DBD-independent for embryonic segmentation but DBD-dependent for deletion of wings and eyes suggesting that where TFs sit on the DBD-dependence/independence spectrum can change both temporally and spatially (Hyduk and Percival-Smith 1996; Percival-Smith 2017). Systematic mutational analysis of the yeast Msn2p TF has uncovered 2 characteristics of occupancy and transcriptional activity. First, occupancy does not require the DBD and instead is dependent on intrinsically disordered regions (IDR) of the protein (Brodsky et al. 2020). Second, there are amino acids important for transcriptional activity but not for occupancy in the IDR, indicating that binding and expression determinants in the IDR are dispersed, separable, and intermingled. These determinants important for both binding and expression are short dispersed elements and sequences of low complexity (Mindel et al. 2024). The analysis of Msn2p was performed in a single cell type and with the same environmental conditions but analysis of TF function/pleiotropy in different tissues/phenotypes indicates that dispersed functional elements of TFs outside the DBD make small differential contributions to overall TF activity (Bhoite et al. 2002; Hittinger et al. 2005; Sivanantharajah and Percival-Smith 2009, 2014, 2015; Percival-Smith et al. 2013), as opposed to TFs being composed of compact domains with specific requirements for TF activity in a particular tissue. In addition, differential pleiotropy of TF function extends to the coordinate regulation of gene expression within a cell. In a yeast cell, Bas1p and Bas2p are 2 TFs required for expression of *HIS4* and *ADE5,7* genes, and the requirement of Bas2p amino acids for expression of *HIS4* and *ADE5,7* differs providing an example of differential pleiotropy within the same cell (Bhoite et al. 2002). Cooperativity in the co-occupancy of a set of 12 TFs associated with the yeast stress response is not mutual but 1 sided: Msn2p binding is required for the binding of the other 11 TFs; however, the other 11 TFs are not required for the binding of Msn2p to its sites (Lupo et al. 2023). In addition, a massively parallel binding assay revealed limited TF binding cooperativity in yeast (Lang et al. 2024). The initiation rate of an actively transcribed gene observed in real time is pulsed and not continuous (Chubb et al. 2006). The TF clusters that form, and believed important for transcription bursting, are dynamic assemblies (Rodriguez and Larson 2020). These observations suggest that the traditional view of how TFs act to regulate the rate of transcription requires revision at the very least.

The limited specificity of TF function and the assembly of TFs into membrane-less compartments, wolfpacks, are distinct models used to explain phenotypic nonspecificity (Percival-Smith 2018; Staller 2022; Percival-Smith et al. 2023). Limited specificity proposes that TFs are independent entities that bind many sites in the genome using relatively nonspecific cooperative interactions and transcription is an emergent property associated with the number of TFs bound to an enhancer/silencer. In this model, TFs regulate distinct patterns of gene expression, and phenotypic nonspecificity is a consequence of the overlap between the distinct patterns of gene expression containing the information required for rescue of the phenotype. In this unbiased model for phenotypic nonspecificity, the transcriptomes of TF-dependent mRNA accumulation should behave stochastically relative to one another which is not supported by the analyses of dsx and ap sets of transcriptomes. The wolfpack along with the 2-step nuclear search model for TF genome occupancy explains DBD independence observed with many yeast TFs (Brodsky et al. 2020; Staller 2022; Kumar et al. 2023). To explain phenotypic nonspecificity, the wolfpack model was modified such that each nucleus contains a discrete number of wolfpacks containing a similar number of TFs that is proportionally related to the frequency of phenotypic nonspecificity. For Drosophila, the estimated number of wolfpacks in the nucleus is 10 to 20 based on a frequency of phenotypic nonspecificity of 1 in 10 to 20 TFs, and therefore, if every cell expresses between 100 and 150 TFs, each wolfpack is composed of between 5 and 15 TFs (Percival-Smith et al. 2023). As each TF in a wolfpack makes a small contribution to the information required to find specific binding sites in a genome, the substitution of 1 TF (TFa) for another (TFb) has little consequence on the sites bound in the genome because the information provided by the other 4 to 14 TFs dominates over that of TFa and TFb for the sites bound such that wolfpacks containing either TFa or TFb share a high percentage of sites (Wunderlich and Mirny 2009). The transcriptomes associated with the expression of TFa and TFb should be similar due to the regulation of a constrained set of genes as observed with most transcriptomes in this study. The clear exception to this is the lack of correlation in the dsx set between the ANTP transcriptome and the other transcriptomes. This lack of correlation is potentially explained by ANTP participating in 2 or more distinct wolfpacks obscuring correlation which would also be consistent with the observation that ANTP has the largest number of DEGs relative to the other TFs studied in the dsx set.

The nucleoplasm is separated into many specialized nonmembrane bound compartments: the nucleolus required for rRNA transcription and transcript processing; histone locus bodies for replication dependent expression of histone mRNAs; and Cajal bodies (Liu et al. 2006; Falahati et al. 2016; Geisler et al. 2023). The wolfpack model, as used to explain phenotypic nonspecificity, suggests that the 100 to 150 TFs expressed in a Drosophila cell are partitioned into 10 to 20 nucleoplasm compartments, and when a compartment interacts with a gene's regulatory DNA sequence, the formation of a transcription hub may be initiated. An expectation of this proposal is the genome occupancy of TFs from the same wolfpack would correlate. Analysis of 141/150 yeast TFs expressed during partial nutrient deprivation shows examples of colocalization/correlation: 23 TFs associated with Cyc8; 12 TFs associated with Med-15; plus 14 to 18 groups composed of 2 to 9 TFs; but 50 to 60 TFs do not correlate strongly with others (Lupo et al. 2023). Although, about two-thirds of yeast TFs show correlation in binding, yeast do not contain 10 to 20 neat and tidy TF compartments of approximately equal size; however, due to the small size of yeast regulatory sequences relative to those of other eukaryotes, it is suggested that the frequency of phenotypic nonspecificity would be lower in yeast in turn suggesting a larger number of nuclear compartments exist in yeast (Percival-Smith 2018).

Although the wolfpack model explains the constrained set of genes regulated by TFs observed, another potential explanation is that TFs bind to regulatory sites after chromatin has been made accessible. Since eukaryotic TFs recognize sequences of low information content, any region of the genome opened up and accessible would be bound by both TFa and TFb explaining the DNA-binding site recognition independence of phenotypic nonspecificity (Wunderlich and Mirny 2009). One model proposed for creating accessible chromatin is that a specialized class of TFs, pioneer TFs, bind to DNA in nucleosomes and open up chromatin allowing access to DNA by other non-pioneer TFs (Zaret 2020). This model has some experimental support and also an exception (Hansen et al. 2022; Brennan et al. 2023). A remaining problem is how pioneer TFs, which themselves recognize DNA-binding sites with low information content, find specific sites in the genome to initiate making chromatin accessible (Wunderlich and Mirny 2009). It is possible that in formation of a wolfpack, pioneering function is imparted to the wolfpack through incorporating a subset of TFs that can interact with DNA in nucleosomes providing entry of the whole wolfpack to specific sites in chromatin.

### Transcriptomes and phenotypes

A common assumption made about the regulation of gene expression and its association with phenotype is that trans-acting regulatory factors active in a cell activate the expression of a specific set of RNAs (transcriptome) and subsequently proteins (translatome) that then fashion the observed phenotype. This assumption is supported at the individual gene level where removal or addition of a function expressed in a restricted temporal and spatial pattern does change phenotype; however, the assumption is only now being tested experimentally at the level of the genome, transcriptome, and translatome. Both phenotypic nonspecificity and phenotypic convergence suggest that unique sets of TFs bring about similar transcriptomes or phenotypes (Konstantinides et al. 2018; Percival-Smith et al. 2023). “You show me which genes are on, I’ll tell you what cell type” is a central tenet important for the interpretation of both scRNAseq and RNAseq analyses (Hahn et al. 2023; Dance 2024). Indeed, in the *Caenorabditis elegans* nervous system, the transcriptome type (t-type) of neurons is associated with a specific morphology (m-type) and function (f-type) types; however, in the zebrafish optic tectum, single t-types can have more than 1 m-type and f-type (Taylor et al. 2021; Shainer et al. 2025). We found that the overall structure of the transcriptome is not a strong predictor of the phenotype generated. This is similar to the intraspecies differences in developmental transcriptomes expressed during sporulation of yeast. Two yeast strains share only 60% of the genes differentially expressed during sporulation (Primig et al. 2000) but both nonetheless sporulate. Although these observations require more examples in a diverse range of species, it should be taken into account in interpretation of any future analyses, as transcriptomes are being used as markers of cancer cell type, which may require more investigation for use as a precise diagnostic tool (Varešlija et al. 2019; Tsimberidou et al. 2022).

RNAseq is inefficient at identifying genes important for a process. In our RNAseq analyses, many genes known to be involved in abdominal pigmentation and wing formation would not have been identified. The systematic functional genomic analysis of yeast sporulation provides 2 other issues with RNAseq analyses: Of the many genes expressed preferentially during sporulation, only a low proportion are required, and of the genes identified by a nonlethal deletion required for sporulation, only 39% are upregulated during sporulation and most surprisingly 21% of the genes required are downregulated (Deutschbauer et al. 2002; Enyenihi and Saunders 2003). Most RNAseq analyses screening for candidates that are required for a particular process ignore downregulated genes. Although some, or much, of the inefficiency observed in yeast sporulation may be due to extensive redundancy, as has been observed during vegetative growth (Costanzo et al. 2010), the poor intraspecies conservation of the transcriptional program associated with sporulation points to major deficiencies in RNAseq analyses for efficiently identifying factors important for developmental processes. A major deficiency in using RNAseq to identify genes regulated directly by the binding of TFs to the gene's regulatory sequence in a multicellular organism composed of many cell types is highlighted in this study by only 4% of AP-dependent mRNA accumulation being assigned to AP-expressing cells.

### Limitations and future studies

Although this study accomplishes its initial goal of distinguishing between 3 general transcriptomic outcomes, it can only speculate on the underlying mechanisms that govern the expression of similar sets of mRNAs by multiple TFs. From the point of view of the study of phenotypic nonspecificity, the dsx and ap phenotypic systems are good in that multiple examples of nonspecificity were identified, but from the perspective of transcriptome analysis, the abdomen and wing imaginal disc are complex organs composed of many cell type specific transcriptomes preventing more far reaching conclusions. For future studies of the mechanism that explains phenotypic nonspecificity, the system employed must have multiple examples of phenotypic nonspecificity. But to thoroughly investigate whether a limited number of wolfpacks and expression programs exist in cells expressing the TF, these cells must have 1 cellular/transcriptomic phenotype and can be isolated in large enough numbers during determination to perform an analysis of occupancy of all the TFs expressed in the cells.

## Supplementary Material

jkaf215_Supplementary_Data

## Data Availability

The raw clean forward read data and extracted count data used for analyses are available on the Gene Expression Omnibus (GEO) at GSE296804. Supplemental material available at G3 online.
