# Supplementary material for: The transcriptomics of phenotypic nonspecificity in Drosophila melanogaster
Source: G3 (Bethesda). 2025 Sep 15;15(11):jkaf215. doi: 10.1093/g3journal/jkaf215 (PMC12611258; doi:10.1093/g3journal/jkaf215)
Supplement: jkaf215_Supplementary_Data [file jkaf215_supplementary_data.pdf]

Supplementary Materials

Table of Contents

Table of Contents	Page
Supplementary Table 1	2
Supplementary Table 2	3-6
Supplementary Figure 1	7-8
Supplementary Figure 2	9
Supplementary Figure 3	10
Supplementary Figure 4	11
Supplementary Figure 5	12
Supplementary Method 1	13-17
Supplementary Method 2	18-19
Supplementary Data 1	20-36

Supplementary Table 1

Genotype	Stock #	Origin
w[*]; TI{GAL4}fru[GAL4.P1.D]/TM3, Sb[1]	66674	Bloomington
y w;P{UASdsx^F, w$^+$}, dsx^1/ TM6B, Tb, P{walLy}		Percival-Smith et al., 2023
y w/ Dp(1;Y)Bar[S]; P{UASbab1, w+}; dsx[1] p[p]/ TM6B, Tb, P{walLy}		Percival-Smith et al., 2023
y w/ Dp(1;Y)Bar[S]; P{UASAntp, w+}; dsx[1] p[p]/ TM6B, Tb, P{walLy}		Percival-Smith et al., 2023
y w/ Dp(1;Y)Bar[S]; P{UASey, w+};dsx[1] p[p]/ TM6B, Tb, P{walLy}		Percival-Smith et al., 2023
y w/ Dp(1;Y)Bar[S]; P{UASodd, w+};dsx[1] p[p]/ TM6B, Tb, P{walLy}		Percival-Smith et al., 2023
y w/ Dp(1;Y)Bar[S]; P{UASfoxo, w+}; dsx[1] p[p]/ TM6B, Tb, P{walLy}		Percival-Smith et al., 2023
y w/Dp(1;Y)Bar[S]; P{UASsqz, w+}; dsx[1] p[p]/ TM6B, Tb, P{walLy}		Percival-Smith et al., 2023
y w; In(2LR)Gla, wg[Gla-1] PPO1[Bc]/CyO, P{Ubi-GFP.S65T, w+}PAD1	4888	Bloomington
y w; ap[MI01996-FlpStop.D]/CyO, P{Ubi-GFP, w+}PAD1		This work
y w; ap[MI01996-FlpStop.D]/CyO, P{Ubi-GFP, w+}PAD1; P{UASap, w+}		This work
y w; ap[MI01996-FlpStop.D]/CyO, P{Ubi-GFP, w+}PAD1; M{UAS-cad.ORF.3xHA.GW}ZH-86Fb		This work
y w; ap[MI01996-FlpStop.D]/CyO, P{Ubi-GFP, w+}PAD1; M{UAS-Myb.ORF}ZH-86Fb		This work
y w; ap[MI01996-FlpStop.D]/CyO, P{Ubi-GFP, w+}PAD1; M{UAS-sisA.ORF.3xHA.GW}ZH-86Fb		This work
y w; ap[MI01996-FlpStop.D]/CyO, P{Ubi-GFP, w+}PAD1; M{UAS-ttk.ORF.3xHA.GW}ZH-86Fb		This work
y w; apGAL4/CyO, P{Ubi-GFP, w+}PAD2		This work

Supplementary Table 2

File name	Genotype	Batch	Date received	# Clean reads	Method
Male1	OreR	1	15-Jan-23	24M	PE100
Male2	OreR	2	23-Mar-23	24M	PE100
Male3	OreR	2	23-Mar-23	24M	PE100
Male4	OreR	3	30-Jun-23	24M	PE100
Male5	OreR	3	30-Jun-23	24M	PE100
Female1	OreR	1	15-Jan-23	24M	PE100
Female2	OreR	2	23-Mar-23	24M	PE100
Female3	OreR	2	23-Mar-23	24M	PE100
Female4	OreR	3	30-Jun-23	24M	PE100
Female5	OreR	3	30-Jun-23	22M	PE100
Condsx1	*w; dsx1/dsxGAL4*	1	15-Jan-23	24M	PE100
Condsx2	*w; dsx1/dsxGAL4*	2	23-Mar-23	24M	PE100
Condsx3	*w; dsx1/dsxGAL4*	2	23-Mar-23	24M	PE100
Condsx4	*w; dsx1/dsxGAL4*	3	30-Jun-23	24M	PE100
Condsx5	*w; dsx1/dsxGAL4*	3	30-Jun-23	24M	PE100
Condsx6	*w; dsx1/dsxGAL4*	3	30-Jun-23	24M	PE100
DSXFdsx1	*w; dsx1, P{UASdsxF}/dsxGAL4*	3	30-Jun-23	24M	PE100
DSXFdsx2	*w; dsx1, P{UASdsxF}/dsxGAL4*	3	30-Jun-23	24M	PE100
DSXFdsx3	*w; dsx1, P{UASdsxF}/dsxGAL4*	3	30-Jun-23	24M	PE100
DSXFdsx4	*w; dsx1, P{UASdsxF}/dsxGAL4*	3	30-Jun-23	24M	PE100
DSXFdsx5	*w; dsx1, P{UASdsxF}/dsxGAL4*	3	30-Jun-23	24M	PE100
BAB1dsx1	*w; P{UASbab1}; dsx1/dsxGAL4*	3	30-Jun-23	24M	PE100
BAB1dsx2	*w; P{UASbab1}; dsx1/dsxGAL4*	3	30-Jun-23	24M	PE100
BAB1dsx3	*w; P{UASbab1}; dsx1/dsxGAL4*	3	30-Jun-23	24M	PE100
BAB1dsx4	*w; P{UASbab1}; dsx1/dsxGAL4*	3	30-Jun-23	24M	PE100
BAB1dsx5	*w; P{UASbab1}; dsx1/dsxGAL4*	3	30-Jun-23	24M	PE100
ANTPdsx1	*w; P{UASAntp}; dsx1/dsxGAL4*	1	15-Jan-23	24M	PE100
ANTPdsx2	*w; P{UASAntp}; dsx1/dsxGAL4*	2	23-Mar-23	24M	PE100
ANTPdsx3	*w; P{UASAntp}; dsx1/dsxGAL4*	2	23-Mar-23	24M	PE100
ANTPdsx4	*w; P{UASAntp}; dsx1/dsxGAL4*	3	30-Jun-23	24M	PE100
ANTPdsx5	*w; P{UASAntp}; dsx1/dsxGAL4*	3	30-Jun-23	24M	PE100
EYdsx1	*w; P{UASey}; dsx1/dsxGAL4*	1	15-Jan-23	24M	PE100
EYdsx2	*w; P{UASey}; dsx1/dsxGAL4*	2	23-Mar-23	24M	PE100
EYdsx3	*w; P{UASey}; dsx1/dsxGAL4*	2	23-Mar-23	24M	PE100
EYdsx4	*w; P{UASey}; dsx1/dsxGAL4*	3	30-Jun-23	24M	PE100

EYdsx5	*w; P{UASey}; dsx1/dsxGAL4*	3	30-Jun-23	24M	PE100
ODDdsx1	*w; P{UASodd}; dsx1/dsxGAL4*	1	15-Jan-23	24M	PE100
ODDdsx2	*w; P{UASodd}; dsx1/dsxGAL4*	2	23-Mar-23	24M	PE100
ODDdsx3	*w; P{UASodd}; dsx1/dsxGAL4*	2	23-Mar-23	24M	PE100
ODDdsx4	*w; P{UASodd}; dsx1/dsxGAL4*	3	30-Jun-23	24M	PE100
ODDdsx5	*w; P{UASodd}; dsx1/dsxGAL4*	3	30-Jun-23	24M	PE100
FOXOdsx1	*w; P{UASfoxo}; dsx1/dsxGAL4*	3	30-Jun-23	22M	PE100
FOXOdsx2	*w; P{UASfoxo}; dsx1/dsxGAL4*	3	30-Jun-23	24M	PE100
FOXOdsx3	*w; P{UASfoxo}; dsx1/dsxGAL4*	3	30-Jun-23	24M	PE100
FOXOdsx4	*w; P{UASfoxo}; dsx1/dsxGAL4*	3	30-Jun-23	24M	PE100
FOXOdsx5	*w; P{UASfoxo}; dsx1/dsxGAL4*	3	30-Jun-23	24M	PE100
SQZdsx1	*w; P{UASsqz}; dsx1/dsxGAL4*	3	30-Jun-23	24M	PE100
SQZdsx2	*w; P{UASsqz}; dsx1/dsxGAL4*	3	30-Jun-23	23M	PE100
SQZdsx3	*w; P{UASsqz}; dsx1/dsxGAL4*	3	30-Jun-23	24M	PE100
SQZdsx4	*w; P{UASsqz}; dsx1/dsxGAL4*	3	30-Jun-23	24M	PE100
SQZdsx5	*w; P{UASsqz}; dsx1/dsxGAL4*	4	14-Nov-23	24M	PE100
WTE31	*y w*	4	14-Nov-23	24M	PE100
WTE32	*y w*	4	14-Nov-23	24M	PE100
WTE33	*y w*	4	14-Nov-23	24M	PE100
WTE34	*y w*	5	30-Jan-24	24M	PE100
WTE35	*y w*	5	30-Jan-24	24M	PE100
ConE31	*y w; apnull/apGAL4*	4	14-Nov-23	24M	PE100
ConE32	*y w; apnull/apGAL4*	4	14-Nov-23	24M	PE100
ConE33	*y w; apnull/apGAL4*	4	14-Nov-23	24M	PE100
ConE34	*y w; apnull/apGAL4*	5	30-Jan-24	24M	PE100
ConE35	*y w; apnull/apGAL4*	5	30-Jan-24	24M	PE100
APE31	*y w; apnull/apGAL4; P{UASap}*	6	23-Apr-24	32M	PE150
APE32	*y w; apnull/apGAL4; P{UASap}*	6	23-Apr-24	24M	PE150
APE33	*y w; apnull/apGAL4; P{UASap}*	6	23-Apr-24	22M	PE150
APE34	*y w; apnull/apGAL4; P{UASap}*	6	23-Apr-24	23M	PE150
CADE31	*y w; apnull/apGAL4; M{UAScad3XHA}*	6	23-Apr-24	27M	PE150
CADE32	*y w; apnull/apGAL4; M{UAScad3XHA}*	6	23-Apr-24	22M	PE150
CADE33	*y w; apnull/apGAL4; M{UAScad3XHA}*	6	23-Apr-24	30M	PE150
CADE34	*y w; apnull/apGAL4; M{UAScad3XHA}*	6	23-Apr-24	28M	PE150
CADE35	*y w; apnull/apGAL4; M{UAScad3XHA}*	6	23-Apr-24	28M	PE150
MYBE31	*y w; apnull/apGAL4; M{UASmyb}*	6	23-Apr-24	33M	PE150
MYBE32	*y w; apnull/apGAL4; M{UASmyb}*	6	23-Apr-24	28M	PE150
MYBE33	*y w; apnull/apGAL4; M{UASmyb}*	6	23-Apr-24	25M	PE150
MYBE34	*y w; apnull/apGAL4; M{UASmyb}*	6	23-Apr-24	29M	PE150

SISAE31	*y w; apnull/apGAL4; M{UASsisA3XHA}*	6	23-Apr-24	28M	PE150
SISAE32	*y w; apnull/apGAL4; M{UASsisA3XHA}*	6	23-Apr-24	23M	PE150
SISAE33	*y w; apnull/apGAL4; M{UASsisA3XHA}*	6	23-Apr-24	28M	PE150
SISAE34	*y w; apnull/apGAL4; M{UASsisA3XHA}*	6	23-Apr-24	26M	PE150
TTKE31	*y w; apnull/apGAL4; M{UASttk3XHA}*	6	23-Apr-24	23M	PE150
TTKE32	*y w; apnull/apGAL4; M{UASttk3XHA}*	6	23-Apr-24	24M	PE150
TTKE33	*y w; apnull/apGAL4; M{UASttk3XHA}*	6	23-Apr-24	28M	PE150
TTKE34	*y w; apnull/apGAL4; M{UASttk3XHA}*	6	23-Apr-24	47M	PE150
TTKE35	*y w; apnull/apGAL4; M{UASttk3XHA}*	6	23-Apr-24	26M	PE150
WTL31	*y w*	4	14-Nov-23	24M	PE100
WTL32	*y w*	4	14-Nov-23	24M	PE100
WTL33	*y w*	4	14-Nov-23	24M	PE100
WTL34	*y w*	4	14-Nov-23	13M	PE100
WTL35	*y w*	4	14-Nov-23	24M	PE100
WTL36	*y w*	5	30-Jan-24	24M	PE100
WTL37	*y w*	5	30-Jan-24	24M	PE100
ConL31	*y w; apnull/apGAL4*	4	14-Nov-23	24M	PE100
ConL32	*y w; apnull/apGAL4*	4	14-Nov-23	24M	PE100
ConL33	*y w; apnull/apGAL4*	4	14-Nov-23	24M	PE100
ConL34	*y w; apnull/apGAL4*	4	14-Nov-23	24M	PE100
ConL35	*y w; apnull/apGAL4*	4	14-Nov-23	23M	PE100
ConL36	*y w; apnull/apGAL4*	4	14-Nov-23	14M	PE100
APL31	*y w; apnull/apGAL4; P{UASap}*	6	23-Apr-24	25M	PE150
APL32	*y w; apnull/apGAL4; P{UASap}*	6	23-Apr-24	22M	PE150
APL33	*y w; apnull/apGAL4; P{UASap}*	6	23-Apr-24	22M	PE150
APL34	*y w; apnull/apGAL4; P{UASap}*	6	23-Apr-24	32M	PE150
APL35	*y w; apnull/apGAL4; P{UASap}*	6	23-Apr-24	21M	PE150
CADL31	*y w; apnull/apGAL4; M{UAScad3XHA}*	5	30-Jan-24	24M	PE100
CADL32	*y w; apnull/apGAL4; M{UAScad3XHA}*	5	30-Jan-24	24M	PE100
CADL33	*y w; apnull/apGAL4; M{UAScad3XHA}*	5	30-Jan-24	24M	PE100
CADL34	*y w; apnull/apGAL4; M{UAScad3XHA}*	5	30-Jan-24	24M	PE100
CADL35	*y w; apnull/apGAL4; M{UAScad3XHA}*	5	30-Jan-24	24M	PE100
MYBL31	*y w; apnull/apGAL4; M{UASmyb}*	6	23-Apr-24	25M	PE150
MYBL32	*y w; apnull/apGAL4; M{UASmyb}*	6	23-Apr-24	23M	PE150
MYBL33	*y w; apnull/apGAL4; M{UASmyb}*	6	23-Apr-24	23M	PE150
MYBL34	*y w; apnull/apGAL4; M{UASmyb}*	6	23-Apr-24	33M	PE150
SISAL31	*y w; apnull/apGAL4; M{UASsisA3XHA}*	5	30-Jan-24	18M	PE100
SISAL32	*y w; apnull/apGAL4; M{UASsisA3XHA}*	5	30-Jan-24	24M	PE100
SISAL33	*y w; apnull/apGAL4; M{UASsisA3XHA}*	5	30-Jan-24	24M	PE100

SISAL34	*y w; apnull/apGAL4; M{UASsisA3XHA}*	5	30-Jan-24	24M	PE100
SISAL35	*y w; apnull/apGAL4; M{UASsisA3XHA}*	5	30-Jan-24	24M	PE100
TTKL31	*y w; apnull/apGAL4; M{UASttk3XHA}*	5	30-Jan-24	24M	PE100
TTKL32	*y w; apnull/apGAL4; M{UASttk3XHA}*	5	30-Jan-24	24M	PE100
TTKL33	*y w; apnull/apGAL4; M{UASttk3XHA}*	5	30-Jan-24	24M	PE100
TTKL34	*y w; apnull/apGAL4; M{UASttk3XHA}*	5	30-Jan-24	24M	PE100
TTKL35	*y w; apnull/apGAL4; M{UASttk3XHA}*	5	30-Jan-24	24M	PE100

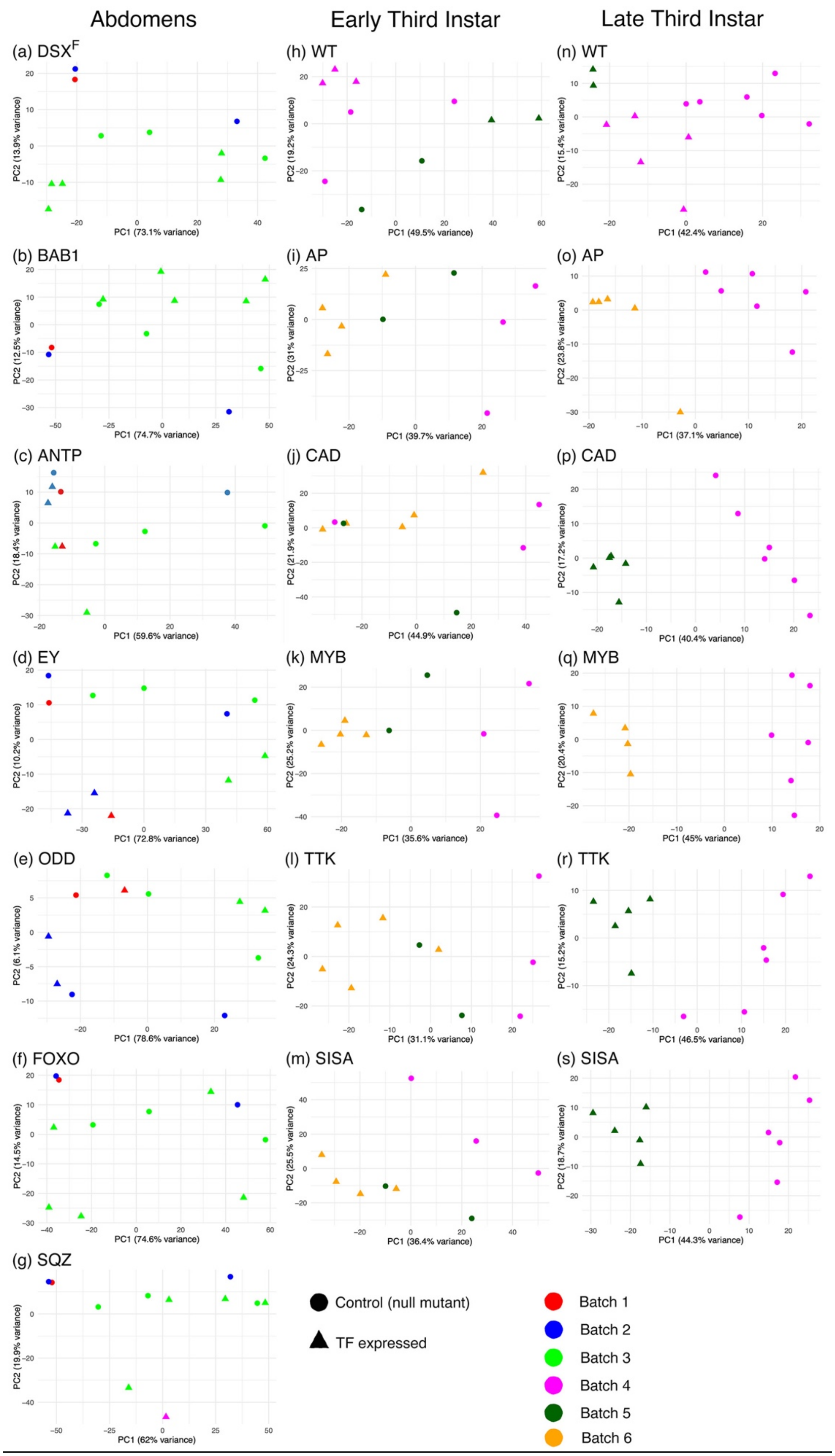
Abdomens
Early Third Instar
Late Third Instar
(a) DSX^F
(b) BAB1
(c) ANTP
(d) EY
(e) ODD
(f) FOXO
(g) SQZ
(h) WT
(i) AP
(j) CAD
(k) MYB
(l) TTK
(m) SISA
(n) WT
(o) AP
(p) CAD
(q) MYB
(r) TTK
(s) SISA
Control (null mutant)
TF expressed
Batch 1
Batch 2
Batch 3
Batch 4
Batch 5
Batch 6

Supplementary Figure 1 Principle component analyses for the DEseq2 data. a-g are replicates of transcriptomes of RNA extracted from abdomens, and all have the same *dsx*^{1}*/dsxGAL4* control but express different TFs: a DSXF; b BAB1; c ANTP; d EY; e ODD; f FOXO; and g SQZ. h-m are replicates of transcriptomes of RNA extracted from early third instar larvae, and all have the same *ap*^{null}*/apGAL4* control but express different TFs: h wild type (ap^{+}); i AP; j CAD; k MYB; l TTK; and m SISA. n-s are replicates of transcriptomes of RNA extracted from early third instar larvae, and all have the same *ap*^{null}*/apGAL4* control but express different TFs: n wild type (ap^{+}); o AP; p CAD; q MYB; r TTK; and s SISA. The control and TF expressing data is represented with circles and triangles, respectively. The sequencing batches are represented by different colors: batch 1 red; batch 2 blue; batch 3 light green; batch 4 purple; batch 5 dark green; and batch 6 orange.

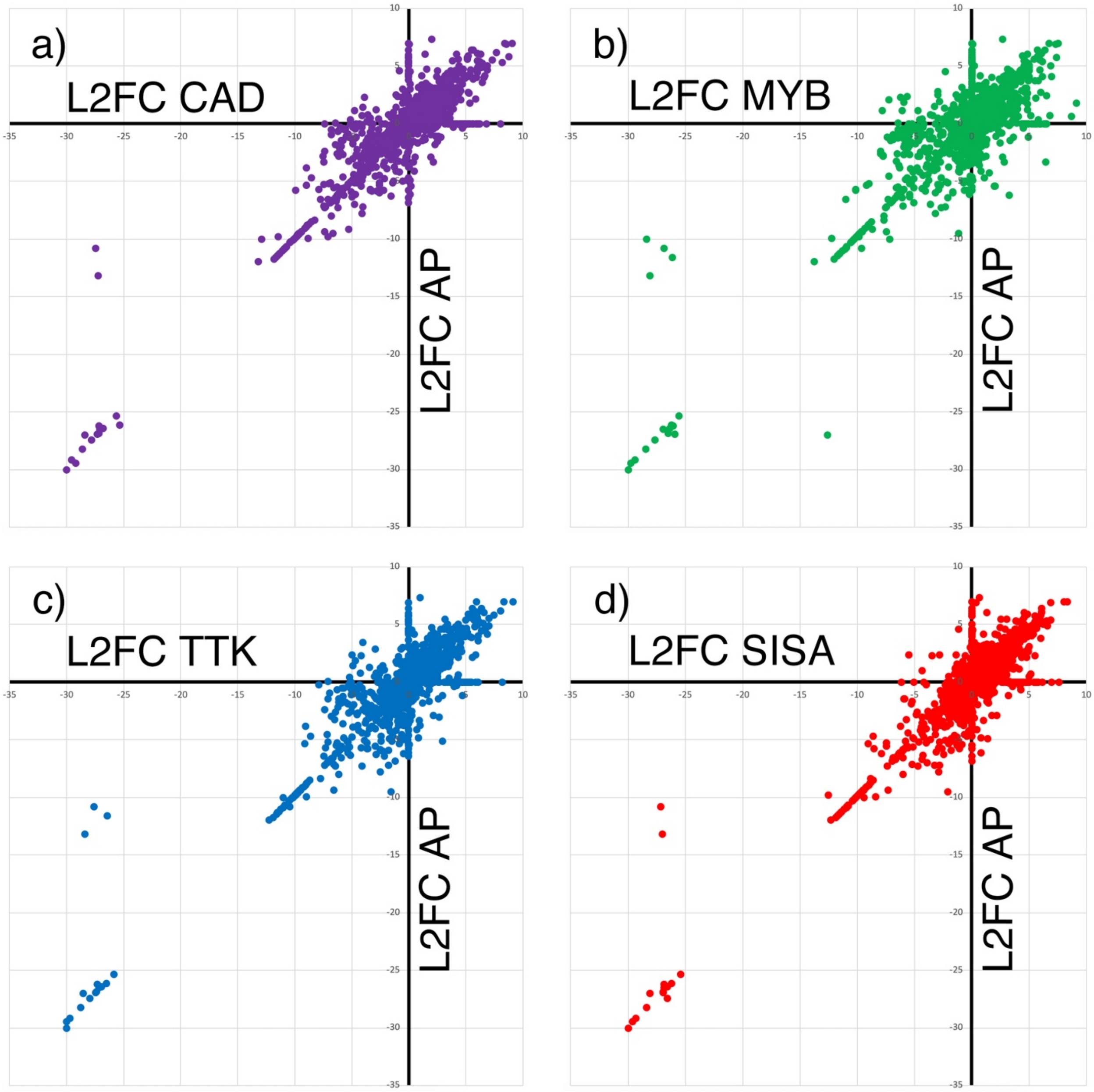

Supplementary Figure 2 Comparisons of $\log_2$ fold changes (L2FC) of non-resident TFs with the changes of the resident TF AP at the early third instar larval stage. In all panels (a-d), the L2FC of the AP dependent transcriptome is plotted on the y-axis. In panels a-d the L2FC of the non-resident TF dependent transcriptomes CAD (a purple), MYB (b green), TTK (c blue) and SISA (d red) are plotted on the x-axis.

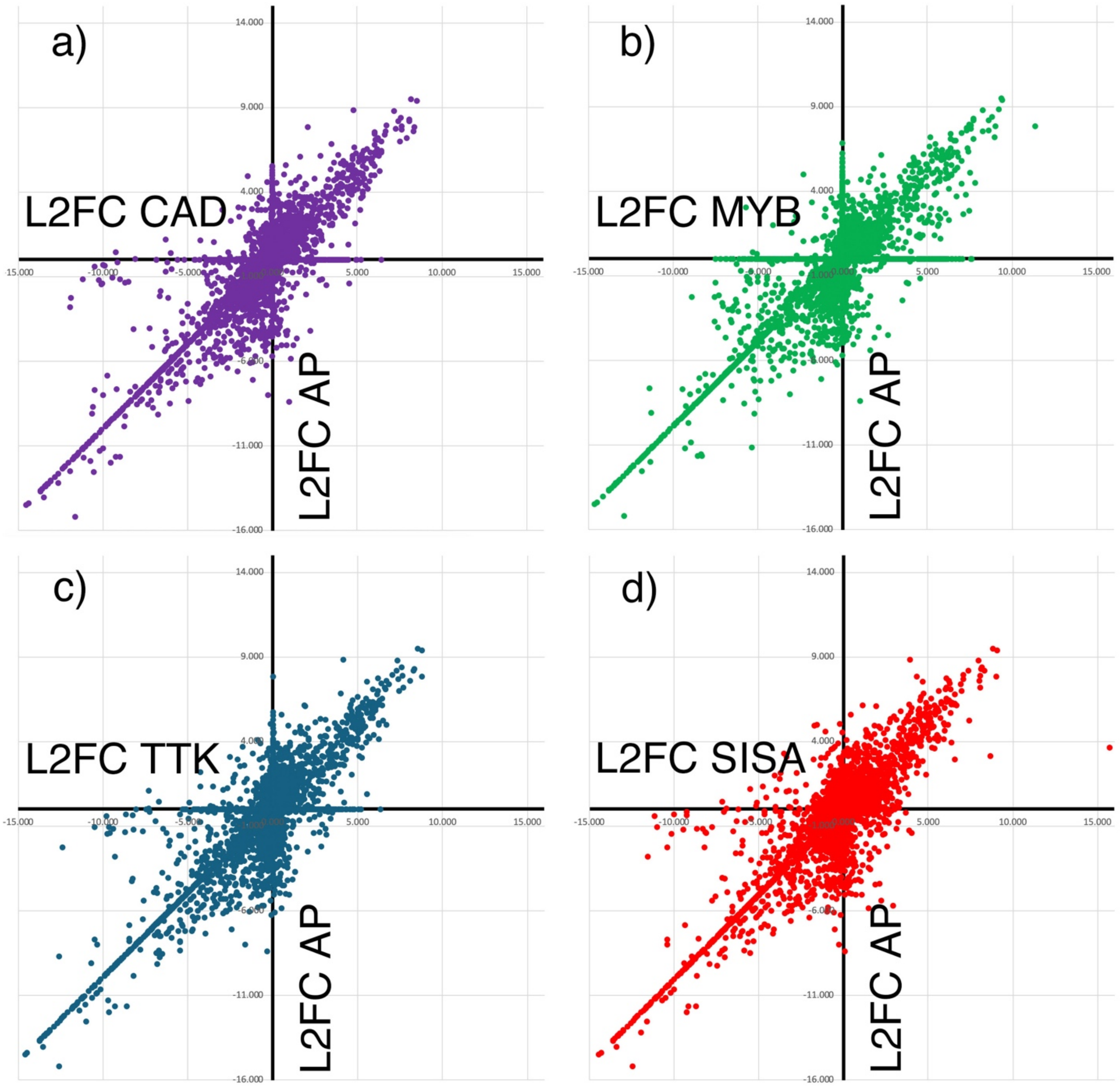

Supplementary Figure 3 Comparisons of log_2 fold changes (L2FC) of non-resident TFs with the changes of the resident TF AP in late third instar larval wing imaginal discs. In all panels (a-d), the L2FC of the AP dependent transcriptome is plotted on the y-axis. In panels a-d the L2FC of the non-resident TF dependent transcriptomes CAD (a purple), MYB (b green), TTK (c blue) and SISA (d red) are plotted on the x-axis.

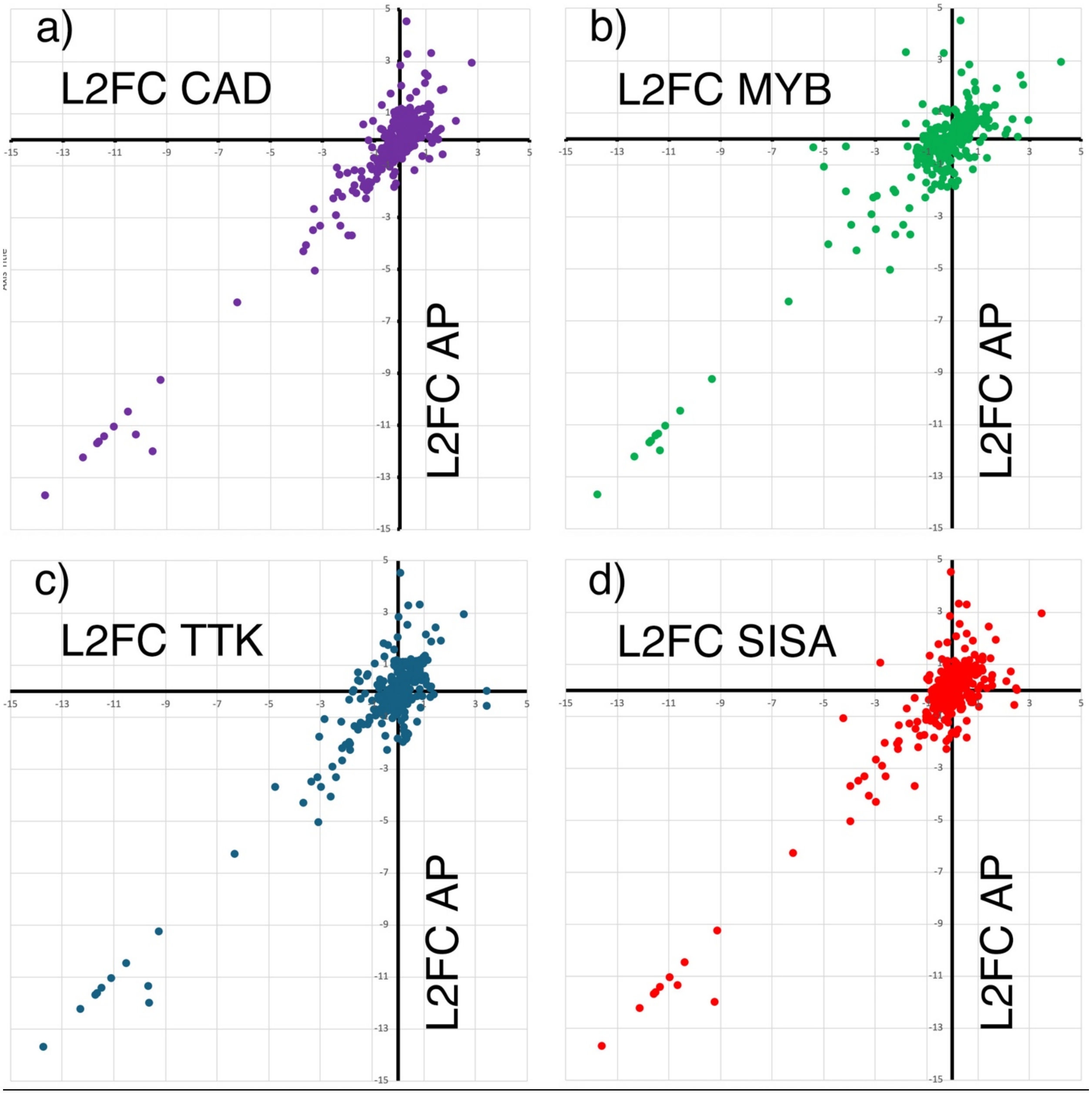

Supplementary Figure 4 Comparisons of $\log_2$ fold changes (L2FC) of non-resident TFs with the changes of the resident TF AP in late third instar larval stage imaginal wing discs of genes expressed in the dorsal wing imaginal disc cells. In all panels (a-d), the L2FC of the AP dependent transcriptome is plotted on the y-axis. In panels a-d the L2FC of the non-resident TF dependent transcriptomes CAD (a purple), MYB (b green), TTK (c blue) and SISA (d red) are plotted on the x-axis.

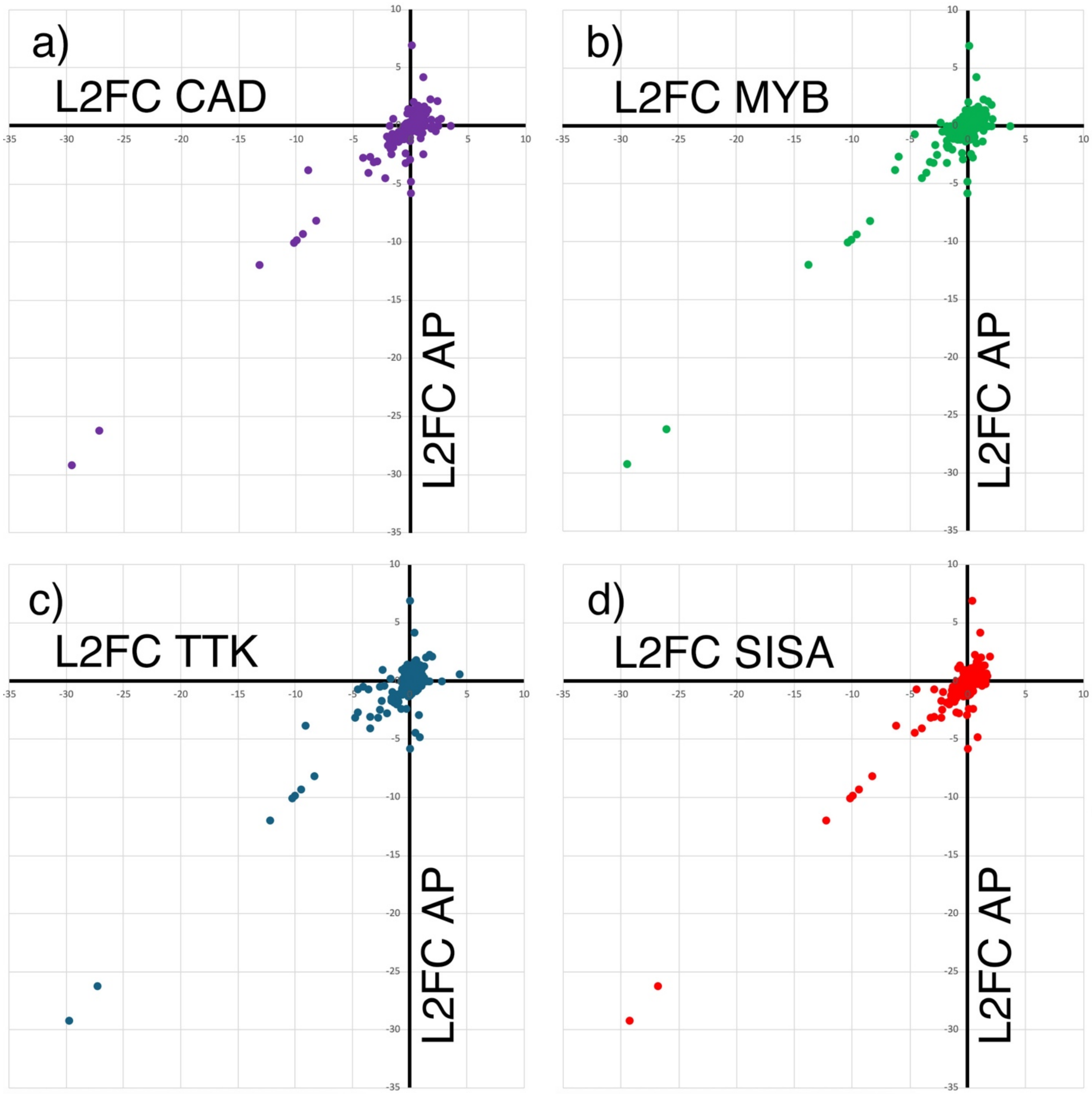

Supplementary Figure 5 Comparisons of log_2 fold changes (L2FC) of non-resident TFs with the changes of the resident TF AP at the early third instar larval stage of genes expressed in the dorsal cells of the wing imaginal disc. In all panels (a-d), the L2FC of the AP dependent transcriptome is plotted on the y-axis. In panels a-d the L2FC of the non-resident TF dependent transcriptomes CAD (a purple), MYB (b green), TTK (c blue) and SISA (d red) are plotted on the x-axis.

Supplementary Method 1

R script used to execute and save the results of the Monte Carlo simulations for random differential expression.

The entire script (steps 1-5) is to be run as a single command. Executing the script once will generate and save a file of 1000 numerical estimates of differential gene expression (DEG) overlap pertaining to a single comparison of transcription factors (TFs).

2 TF comparison

```
# 1. Set parameters
num_simulations <- 1000                    # Number of simulations to be performed
num_genes_condition1 <- 14841              # Number of total expressed genes by TF 1
num_genes_condition2 <- 14919              # Number of total expressed genes by TF 2
num_diff_exp_genes_condition1 <- 2892      # Number of DEGs by TF 1
num_diff_exp_genes_condition2 <- 3404      # Number of DEGs by TF 2

# 2. Define a function to generate random sets of DEGs
generate_diff_exp_genes <- function(total_genes, num_diff_exp_genes) {
  diff_exp_genes <- sample(1:total_genes, num_diff_exp_genes, replace = FALSE)
  return(diff_exp_genes)
}

# 3. Perform simulations
overlap_counts <- numeric(num_simulations)
for (i in 1:num_simulations) {
  diff_exp_genes_condition1 <- generate_diff_exp_genes(num_genes_condition1,
num_diff_exp_genes_condition1)
  diff_exp_genes_condition2 <- generate_diff_exp_genes(num_genes_condition2,
num_diff_exp_genes_condition2)

# 4. Count the number of overlapping genes between the 2 randomized sets of DEGs in each
simulation
  overlap_counts[i] <- length(intersect(diff_exp_genes_condition1,
diff_exp_genes_condition2))
}
```

```
# 5. Save the 1000 numerical estimates of DEG overlap to the current directory
write.table(overlap_counts, file = "overlap_counts_condition1_condition2.txt", row.names =
FALSE, col.names = FALSE)
```

3 TF comparison

```
# 1. Set parameters
num_simulations <- 1000                 # Number of simulations to be performed
num_genes_condition1 <- 14951           # Number of total expressed genes by TF 1
num_genes_condition2 <- 14919           # Number of total expressed genes by TF 2
num_genes_condition3 <- 14796           # Number of total expressed genes by TF 3
num_diff_exp_genes_condition1 <- 4077   # Number of DEGs by TF 1
num_diff_exp_genes_condition2 <- 3404   # Number of DEGs by TF 2
num_diff_exp_genes_condition3 <- 3353   # Number of DEGs by TF 3

# 2. Define a function to generate random sets of DEGs
generate_diff_exp_genes <- function(total_genes, num_diff_exp_genes) {
   diff_exp_genes <- sample(1:total_genes, num_diff_exp_genes, replace = FALSE)
   return(diff_exp_genes)
}

# 3. Perform simulations
overlap_counts <- numeric(num_simulations)
for (i in 1:num_simulations) {
   diff_exp_genes_condition1 <- generate_diff_exp_genes(num_genes_condition1,
num_diff_exp_genes_condition1)
   diff_exp_genes_condition2 <- generate_diff_exp_genes(num_genes_condition2,
num_diff_exp_genes_condition2)
   diff_exp_genes_condition3 <- generate_diff_exp_genes(num_genes_condition3,
num_diff_exp_genes_condition3)

# 4. Count the number of overlapping genes between the 2 randomized sets of DEGs in each
simulation
   overlap_counts[i] <- length(Reduce(intersect, list(diff_exp_genes_condition1,
diff_exp_genes_condition2, diff_exp_genes_condition3)))
}


# 5. Save the 1000 numerical estimates of DEG overlap to the current directory
write.table(overlap_counts, file = "overlap_counts_condition1_condition2.txt", row.names =
FALSE, col.names = FALSE)
```

4 TF comparison

```
# 1. Set parameters
num_simulations <- 1000                    # Number of simulations to be performed
num_genes_condition1 <- 14841              # Number of total expressed genes by TF 1
num_genes_condition2 <- 14951              # Number of total expressed genes by TF 2
num_genes_condition3 <- 14919              # Number of total expressed genes by TF 3
num_genes_condition4 <- 14796              # Number of total expressed genes by TF 4
num_diff_exp_genes_condition1 <- 2892      # Number of DEGs by TF 1
num_diff_exp_genes_condition2 <- 4077      # Number of DEGs by TF 2
num_diff_exp_genes_condition3 <- 3404      # Number of DEGs by TF 3
num_diff_exp_genes_condition4 <- 3353      # Number of DEGs by TF 4

# 2. Define a function to generate random sets of DEGs
generate_diff_exp_genes <- function(total_genes, num_diff_exp_genes) {
  diff_exp_genes <- sample(1:total_genes, num_diff_exp_genes, replace = FALSE)
  return(diff_exp_genes)
}

# 3. Perform simulations
overlap_counts <- numeric(num_simulations)
for (i in 1:num_simulations) {
  diff_exp_genes_condition1 <- generate_diff_exp_genes(num_genes_condition1,
num_diff_exp_genes_condition1)
  diff_exp_genes_condition2 <- generate_diff_exp_genes(num_genes_condition2,
num_diff_exp_genes_condition2)
  diff_exp_genes_condition3 <- generate_diff_exp_genes(num_genes_condition3,
num_diff_exp_genes_condition3)
  diff_exp_genes_condition4 <- generate_diff_exp_genes(num_genes_condition4,
num_diff_exp_genes_condition4)

# 4. Count the number of overlapping genes between the 2 randomized sets of DEGs in each
simulation
  overlap_counts[i] <- length(Reduce(intersect, list(diff_exp_genes_condition1,
diff_exp_genes_condition2, diff_exp_genes_condition3, diff_exp_genes_condition4)))
}

# 5. Save the 1000 numerical estimates of DEG overlap to the current directory
write.table(overlap_counts, file = "overlap_counts_condition1_condition2.txt", row.names =
FALSE, col.names = FALSE)
```

5 TF comparison

```
# 1. Set parameters
num_simulations <- 1000                    # Number of simulations to be performed
total_genes <- c(
  condition1 = 14841,                      # Number of total expressed genes by TF 1
  condition2 = 14951,                      # Number of total expressed genes by TF 2
  condition3 = 14919,                      # Number of total expressed genes by TF 3
  condition4 = 14796,                      # Number of total expressed genes by TF 4
  condition5 = 15000                       # Number of total expressed genes by TF 5
)

diff_exp_genes <- c(
  condition1 = 2892,                       # Number of DEGs by TF 1
  condition2 = 4077,                       # Number of DEGs by TF 2
  condition3 = 3404,                       # Number of DEGs by TF 3
  condition4 = 3353,                       # Number of DEGs by TF 4
  condition5 = 3000                        # Number of DEGs by TF 5
)

# 2. Define a function to generate random sets of DEGs
generate_diff_exp_genes <- function(total_genes, num_diff_exp_genes) {
  diff_exp_genes <- sample(1:total_genes, num_diff_exp_genes, replace = FALSE)
  return(diff_exp_genes)
}

# 3. Perform simulations
overlap_counts <- numeric(num_simulations)
for (i in 1:num_simulations) {
  diff_exp_genes_list <- lapply(names(total_genes), function(condition)
generate_diff_exp_genes(total_genes[condition], diff_exp_genes[condition]))

# 4. Count the number of overlapping genes between the 2 randomized sets of DEGs in each
simulation
  overlap_counts[i] <- length(Reduce(intersect, diff_exp_genes_list))
}


# 5. Save the 1000 numerical estimates of DEG overlap to the current directory
write.table(overlap_counts, file = "overlap_counts_condition1_condition2.txt", row.names =
FALSE, col.names = FALSE)
```

6 TF comparison

```
# 1. Set parameters
num_simulations <- 1000                # Number of simulations to be performed
total_genes <- c(
  condition1 = 14841,                  # Number of total expressed genes by TF 1
  condition2 = 14951,                  # Number of total expressed genes by TF 2
  condition3 = 14919,                  # Number of total expressed genes by TF 3
  condition4 = 14796,                  # Number of total expressed genes by TF 4
  condition5 = 15000,                  # Number of total expressed genes by TF 5
  condition6 = 15200                   # Number of total expressed genes by TF 6
)

diff_exp_genes <- c(
  condition1 = 2892,                   # Number of DEGs by TF 1
  condition2 = 4077,                   # Number of DEGs by TF 2
  condition3 = 3404,                   # Number of DEGs by TF 3
  condition4 = 3353,                   # Number of DEGs by TF 4
  condition5 = 3000,                   # Number of DEGs by TF 5
  condition6 = 3200                    # Number of DEGs by TF 6
)

# 2. Define a function to generate random sets of DEGs
generate_diff_exp_genes <- function(total_genes, num_diff_exp_genes) {
  diff_exp_genes <- sample(1:total_genes, num_diff_exp_genes, replace = FALSE)
  return(diff_exp_genes)
}

# 3. Perform simulations
overlap_counts <- numeric(num_simulations)
for (i in 1:num_simulations) {
  diff_exp_genes_list <- lapply(names(total_genes), function(condition)
generate_diff_exp_genes(total_genes[condition], diff_exp_genes[condition]))

# 4. Count the number of overlapping genes between the 2 randomized sets of DEGs
  overlap_counts[i] <- length(Reduce(intersect, diff_exp_genes_list))
}

# 5. Save the 1000 numerical estimates of DEG overlap to the current directory
write.table(overlap_counts, file = "overlap_counts_condition1_condition2.txt", row.names =
FALSE, col.names = FALSE)
```

Supplementary Method 2

R script used to print any number of virtual in-situ images using the code written by Everetts et al., 2021

The entire script (steps 1-4) is to be run as a single command. Executing the script once will generate and save an image of all genes in your list (‘genes_of_interest’), preserving their order. In RStudio, the dimensions (height and width) of the images will match those of the current 'Plots' window (where each image appears).

To ensure accurate results, do not use your computer for other tasks while the script is running. Creating multiple images with this script is computationally intensive. The script will finish running after it has printed and saved images of all genes in your list. Therefore, longer lists will take a greater amount of time to complete. If using RStudio, do not alter the size of the ‘Plots’ window while the script is running, as this will cause the R session to abort. For this reason, adjust the window to the desired size before running the script.

The script will also stop running if it encounters a gene in your list that is not found within the scRNA-seq correlation matrix. Previously printed and saved images will not be deleted if this occurs.

```
# 1. Create list of genes you would like to make images of
genes_of_interest <- c('FBgn0267978', 'FBgn0267978', 'FBgn0027790')

# 2. Define a function to create and save the image of 1 gene
create_and_save_image <- function(gene, output_filename) {
  VISH <- computeVISH(dmDiscImputed, gene, threshold = 0)
  VISHcolor <- pickcolor(VISH, 0.5, 1)

  toGraph <- as.data.frame(cbind(geometryfile, VISHcolor))
  Nzlayers <- unique(toGraph$z)
  plotsHere <- list()
  counter <- 1

  for (n in Nzlayers) {
    tosave <- toGraph[toGraph$z == n,]
    p1 <- ggplot(data = tosave, mapping = aes(x, y, color = VISHcolor)) +
      xlim(min(toGraph$x), max(toGraph$x)) +
      ylim(min(toGraph$y), max(toGraph$y)) +
      theme_void() +
      geom_point(size = 5, show.legend = FALSE, alpha = 1) +
      scale_colour_gradientn(colours = viridis, limits = c(min(toGraph$VISHcolor),
max(toGraph$VISHcolor)))
    plotsHere[[counter]] <- p1
    counter <- counter + 1
  }

g <- grid.arrange(grobs = plotsHere, ncol = length(plotsHere), top = paste(gene))
  ggsave(filename = output_filename, plot = g, device = "png")
}

# 3. Repeat step 2 for each gene in 'genes_of_interest'
for (gene in genes_of_interest) {
  output_filename <- paste(gene, "_virtual_in_situ.png", sep = "")  # Include gene name in the
filename
  create_and_save_image(gene, output_filename)
}
```

Supplementary Data 1

The sequence of the UAS constructs used with Salmon to estimate expression.

UAS dsxF (2,032 bases)

CAAGAAGAGAACTCTGAATAGGGAATTGGGAATTCATGGTTTCGGAGGAGACTGGA
ATAGCGACACGATGTCCGACTCGGACATGATCGACTCAAAGAACGACGTCTGTGGC
GGAGCCTCCAGTTCCAGCGGCAGCTCGATTTCGCCGAGGACACCACCGAACTGCGC
CCGCTGCCGCAATCATGGCCTAAAGATCACCCTAAAGGGACACAAGCGGTACTGCA
AGTTCCGCTACTGCACGTGCGAGAAGTGCCGACTGACGGCGGACCGCCAGCGGGTG
ATGGCTCTGCAAACGGCCTTGAGGCGAGCCCAGGCGCAGGATGAGCAGCGGGCGCT
GCACATGCACGAGGTGCCGCCTGCCAATCCGGCGGCCACGACCCTCCTGAGTCATC
ACCATCATGTCGCAGCTCCCGCTCATGTCCACGCCCACCATGTGCACGCCCATCACG
CACACGGAGGACACCACTCGCATCACGGACATGTCCTGCACCACCAGCAGGCAGCG
GCGGCCGCAGCAGCAGCTCCGTCGGCTCCAGCCTCCCATCTGGGTGGCTCCAGCACG
GCCGCTTCCAGCATCCACGGTCACGCCCACGCGCACCACGTCCACATGGCAGCTGCT
GCAGCCGCCTCGGTGGCTCAGCACCAGCACCAAAGCCACCCACACTCGCACCACCA
CCACCACCAGAACCACCACCAGCATCCGCATCAGCAACCGGCCACGCAGACCGCCC
TGCGATCTCCGCCGCACAGCGACCATGGTGGCAGTGTGGGTCCGGCCACCAGCAGC
TCCGGCGGTGGAGCACCCAGTTCCAGCAATGCGGCAGCGGCCACCTCGAGCAACGG
ATCCAGCGGCGGTGGAGGAGGAGGCGGAGGGGGCAGCTCGGGTGGCGGGGCAGGA
GGTGGTAGGTCATCGGGAACATCGGTGATCACTAGCGCCGATCACCACATGACCAC
GGTGCCTACGCCCGCCCAATCGCTGGAGGGGTCCTGCGACTCGTCGTCGCCCTCGCC
GTCGTCCACTTCCGGTGCAGCCATTTTGCCGATCTCAGTTTCCGTCAATCGAAAGAA
CGGCGCCAATGTGCCCTTGGGCCAAGACGTTTTCCTAGACTATTGCCAAAAGCTATT
AGAAAAATTCCGCTATCCTTGGGAGCTGATGCCACTCATGTATGTGATATTAAAGGA
CGCAGACGCCAACATTGAAGAGGCTTCCCGGCGAATCGAAGAGGGCCAATACGTTG
TGAATGAGGAATATTATAAAAAGGATAAACCTGAAATGTCAGAAATAAACTATTTT
CCTTAAACCTCGAGGGTACCTCTAGAGGATCTTTGTGAAGGAACCTTACTTCTGTGG
TGTGACATAATTGGACAAACTACCTACAGAGATTTAAAGCTCTAAGGTAAATATAA
AATTTTTAAGTGTATAATGTGTTAAACTACTGATTCTAATTGTTTGTGTATTTTAGAT
TCCAACCTATGGAACTGATGAATGGGAGCAGTGGTGGAATGCCTTTAATGAGGAAA
ACCTGTTTTGCTCAGAAGAAATGCCATCTAGTGATGATGAGGCTACTGCTGACTCTC
AACATTCTACTCCTCCAAAAAAGAAGAGAAAGGTAGAAGACCCCAAGGACTTTCCT
TCAGAATTGCTAAGTTTTTTGAGTCATGCTGTGTTTAGTAATAGAACTCTTGCTTGCT
TTGCTATTTACACCACAAAGGAAAAAGCTGCACTGCTATACAAGAAAATTATGGAA
AAATATTTGATGTATAGTGCCTTGACTAGAGATCATAATCAGCCATACCACATTTGT
AGAGGTTTTACTTGCTTTAAAAAACCTCCCACACCTCCCCCTGAACCTGAAACATAA
AATGAATGCAATTGTTGTTGTTAACTTGTTTATTGCAGCTTATAATGGTTACAAATAA
AGCAATAGCATCACAAATTTCACAAATAAAGCATTTTTTTCACTGCATTCTAGTTGT
GGTTTGTCCAAACTCATCAATGTNNCTTATCATGTCTGGATCCACTAGAAGGCC

UASdsxM (2,413 bases)

CAAGAAGAGAACTCTGAATAGGGAATTGGGAATTCGATGGTTTCGGAGGAGAACTG
GAATAGCGACACGATGTCCGACTCGGACATGATCGACTCAAAGAACGACGTCTGTG
GCGGAGCCTCCAGTTCCAGCGGCAGCTCGATTTGCCGAGGACACCACCGAATCGCG
CCCGCTGCCGCAATCATGGCCTAAAGATCACCCTAAAGGGACACAAGCGGTACTGC
AAGTTCCGCTACTGCACGTGCGAGAAGTGCCGACTGACGCGGACCGCCAGCGGGTG
ATGGCTCTGCAAACGGCCTTGAGGCGAGCCCAGGCGCAGGATGAGCAGCGGGCGCT
GCACATGCACGAGGTGCCGCCTGCCAATCCGGCGGCCACGACCCTCCTGAGTCATC
ACCATCATGTCGCAGCTCCCGCTCATGTCCACGCCACCAGTGGCACGCCCATCACGC
ACACGGAGGACACCACTCGCATCACGGACATGTCCTGCACCACCAGCAGGCAGCGG
CGGCCGCAGCAGCAGCTCCGTCGGCTCCAGCCTCCCATCTGGGTGGCTCCAGCACGG
CCGCTTCCAGCATCCACGGTCACGCCCACGCGCACCACGTCCACATGGCAGCTGCTG
CAGCCGCCTCGGTGGCTCAGCACCAGCACCAAAGCCACCCACACTCGCACCACCAC
CACCACCAGAACCACCACCAGCATCCGCATCAGCAACCGGCCACGCAGACCGCCCT
GCGATCTCCGCCGCACAGCGACCATGGTGGCAGTGTGGGTCCGGCCACCAGCAGCT
CCGGCGGTGGAGCACCCAGTTCCAGCAATGCGGCAGCGGCCACCTCGAGCAACGGA
TCCAGCGGCGGTGGAGGAGGAGGCGGAGGGGGCAGCTCGGGTGGCGGGGCAGGAG
GTGGTAGGTCATCGGGAACATCGGTGATCACTAGCGCCGATCACCACATGACCACG
GTGCCTACGCCCGCCCAATCGCTGGAGGGGTCCCGCGACTCGTCGTCGCCCTCGCCG
TCGTCCACTTCCGGTGCAGCCATTTTGCCGATCTCAGTTTCCGTCAATCGAAAGAAC
GGCGCCAATGTGCCCTTGGGCCAAGACGTTTTCCTAGACTATTGCCAAAAGCTATTA
GAAAAATTCCGCTATCCTTGGGAGCTGATGCCACTCATGTATGTGATATTAAAGGAC
GCAGACGCCAACATTGAAGAGGCTTCCCGGCGGAATCGAAGAGGCTCGAGTGGAAA
TAAATCGCACTGTAGCCCGAGATCTACTACAACTACTACACCCCGATGGCCCTGGTG
AACGGGGCGCCCATGTACCTGACCTACCCGAGCATCGAACAGGGTCGCTATGGGGC
GCACTTCACCCATCTGCCGCTCACACAGATTTGTCCACCGACTCCAGAGCCGCTGGC
CCTCAGCCGCTCCCCGAGCAGTCCCAGTGGACCGTCGGCTGTCCACAACCAAAAGC
CCTCCCGACCGGGCAGCAGCAATGGCACCGTCCACTCCGCGGCCTCACCCACAATG
GTCACCACGATGGCCACGACCTCCTCCACGCCCACGCTCAGCCGCCGTCAGAGATCG
CGCTCGGCCACGCCCACCACTCCGCCACCACCGCCACCGGCGCACAGCAGCAGCAA
CGGAGCCTACCACCACGGCCACCACCTGGTCAGCTCCACGGCTGCCACGTAGCTCG
AGGGTACCTCTAGAGGATCTTTGTGAAGGAACCTTACTTCTGTGGTGTGACATAATT
GGACAAACTACCTACAGAGATTTAAAGCTCTAAGGTAAATATAAAATTTTTAAGTGT
ATAATGTGTTAAACTACTGATTCTAATTGTTTGTGTATTTTAGATTCCAACCTATGGA
ACTGATGAATGGGAGCAGTGGTGGAATGCCTTTAATGAGGAAAACCTGTTTTGCTCA
GAAGAAATGCCATCTAGTGATGATGAGGCTACTGCTGACTCTCAACATTCTACTCCT
CCAAAAAAGAAGAGAAAGGTAGAAGACCCCAAGGACTTTCCTTCAGAATTGCTAAG
TTTTTTGAGTCATGCTGTGTTTAGTAATAGAACTCTTGCTTGCTTTGCTATTTACACC
ACAAAGGAAAAAGCTGCACTGCTATACAAGAAAATTATGGAAAAATATTTGATGTA
TAGTGCCTTGACTAGAGATCATAATCAGCCATACCACATTTGTAGAGGTTTTACTTG
CTTTAAAAAACCTCCCACACCTCCCCCTGAACCTGAAACATAAAATGAATGCAATTG
TTGTTGTTAACTTGTTTATTGCAGCTTATAATGGTTACAAATAAAGCAATAGCATCAC
AAATTTCACAAATAAAGCATTTTTTTCACTGCATTCTAGTTGTGGTTTGTCCAAACTC
ATCAATGTNNCTTATCATGTCTGGATCCACTAGAAGGCC

UAS BAB1 (4,443 bases)

CAAGAAGAGAACTCTGAATAGGGAATTGGGAATTCGTTAACAGATCTTGCGGCCGC
GCTCGAGGGTACCAGACGAATGTCGGCTTGGCGTCCGAACAGGGACCAGTGGCTCA
GAGGCAGCGCAAAGGGACGGGATCGGGCGCCGATTCGCCCAAGAGTAACAGAAGC
TCGCCCACTCAGCAGGAGGAGAAGCGTATCAAAAGCGAGGATCGCACTTCACCAAC
TGGCGGGGCCAAGGACGAGGACAAGGAGAGTCAAGGTCATGCTGTAGCCGGAGGG
GGAGGATCTTCGCCCGTCAGTTCGCCACAGGGCAGGAGTTCTTCGGTAGCCTCGCCC
AGTTCCAGCTCCCAGCAATTCTGCCTGCGCTGGAACAACTATCAGACGAACCTGACC
ACCATCTTTGACCAGCTGCTCCAGAACGAGTGCTTCGTGGACGTGACCTTGGCATGC
GATGGTCGGTCCATGAAGGCCCACAAGATGGTCCTGTCCGCCTGCTCGCCCTACTTC
CAAACACTTCTGGCCGAAACGCCCTGCCAGCATCCCATTGTGATCATGCGGGACGTA
AATTGGTCGGATCTCAAGGCCATTGTGGAGTTCATGTATCGCGGCGAGATCAACGTG
AGCCAGGACCAGATAGGTCCTCTGCTCAGGATAGCTGAGATGTTGAAAGTGCGTGG
TCTGGCGGATGTGACCCATATGGAGGCGGCCACGGCAGCAGCGGCTGCCGCTTCGT
CGGAGAGAATGCCCTCCTCGCCCAAGGAGAGCACTTCAACTTCCAGAACTGAACAC
GACAGGGAACGGGAGGCCGAGGAGCTGCTGGCCTTCATGCAGCCCGAGAAGAAGC
TACGCACTTCGGACGGGATCCCGCTGAGCTGAGGCTCTCCCCACTGGAGCGGCAGC
AGGGCAGGAATGTAAGAAAGCGCCGGTGGCCATCGGCGGACACAATATTCAATCCA
CCCGCACCACCCAGTCCACTGAGCAGCCTGATTGCGGCCGAAAGGATGGAGCTGGA
GCAAAAGGAAAGAGAGAGACAGAGGGACTGTTCGCTGATGACACCCCCACCCAAA
CCACCAATGAGCAGTGGCTCCACAGTGGGAGCCACGAGGCGCCTGGAGACCGCCAT
CCACGCCCTGGACATGCCATCGCCGGCTGCCACGCCAGGACCTCTGTCCCGATCGTC
GAGACCTCACTCGCAGAGCCCCCAGCAGCAGCAGGCACAGCAGCAGGGTCAGCTTC
CTTTGCCCCTGCCCCTGCATCCGCACCATCACGCATCACCCGCCCCACATCCCTCCCA
GACCGCCGGATCAGCCCACCACCCGGCATCGCCTGCTGGAGATTCCCGTTTTCCCCT
CGGCCCAGCAGCCGCCATGGCCGCTGCCAGGGAACTGAGTGGCCTGGGACCAGGTC
CGTCCGCCGAGCCACGCCTTCCGCCTCCACCGCCGCACCACCATGGCGGTGGTGGAG
TGGGCGGCGGGGGAGTTGGAGGAGGAGGTGCAGGCGGAGTGGGTTCAGGCGGGGG
ATCCTCGCTCGCCGATGACTTGGAGATCAAGCCAGGGATCGCCGAGATGATCCGAG
AGGAAGAAAGGGCCAAAATGATGGAGAACTCGCACGCCTGGATGGGCGCCACCGG
ATCAACGCTGGCAGCAGACAGCTACCAGTACCAGCTGCAGTCCATGTGGCAAAAGT
GCTGGAACACCAACCAGAATCTGATGCATCACATGCGCTTCCGCGAGCGAGGTCCT
CTGAAGTCGTGGCGACCCGAGACCATGGCGGAGGCCATTTTCAGTGTGCTAAAGGA
GGGTCTATCGCTATCTCAGGCCGCCCGCAAGTACGACATCCCGTATCCAACATTCGT
GCTCTATGCGAACAGGGTGCACAATATGCTGGGACCATCCATTGACGGCGGGCCCG
ATTTGCGGCCCAAGGGGCGTGGCAGGCCGCAGCGAATCCTTCTGGGCATCTGGCCC
GACGAGCACATTAAGGGCGTCATCAAGACGGTGGTCTTTCGCGACACCAAGGACAT
CAAGGACGAGAGCCTGGCCGCTCACATGCCACCCTACGGTCGACATTCGCCCGCGTT
TCCCTTGCAGGACCTCCCTCTCAGCTATCCCGGAGCCAGTGGCGCCCTGGCAGGCGC
GCCCAGCTCCATGGCCTGTCCGAATGGCAGTGGACCGCAGACCGGAGTGGGCGTGG
CCGGAGAGCAGCATATGTCACAGGAAACGGCCGCCGCGGTGGCCGCCGTGGCGCAC
AACATCCGCCAGCAGATGCAAATGGCAGCGGTTCCGCCCGGCTTATTCAATCTGCCG
CCTCATCCGGGAGTGGGCGGTGGAGTGGGCAACGTTCCCGGCGCAGCTGGAGGCAG
GGCCAGCATATCGCCGGCCCTGAGCAGTGGCTCCGGACCAAGGCACGCTCCCTCGC
CCTGCGGTCCCGCCGGCCTCCTGCCGAACCTGCCGCCCAGCATGGCCGTCGCTCTGC
ACCACCAGCAGCAACAGCAGGCGGCGCACCACCACATGCAGCAGCTGCACCTGCAG
CAGCAACAGGCCCACTTGCACCACCATCAGCAGCAACAGCAACAGCAGCAGCAGCA

GCACCATCAGGGCGGCCATCAGGTGGCCCACAAGTCCGGTTTCGGTGCCAGCTCCA
GTTCCTCAGCCTCCTCGTCGTCAATGGGCCAGCACCATGCGCCCAAGGCCAAGAGCA
GTCCGTTGCGCAGCGAAACGCCTCGGCTGCACTCCCCGCTCGGCGATCTTGGCCTGG
ACATGGCCAGCTACAAGCGCGAATTCTCGCCCAGCCGCCTCTTCGCCGAGGATCTGG
CCGAGCTGGTGGGCGCCAGTGTCTCATCTTCCTCATCATCGGCGGCGGCAGCGACGG
CTCCTCCGGAAAGATCGGCAGGAGCAGCTTCCGCAGCCACAGGCGCGGATGCACCC
AGTTCCTCGAGCAGTGGAGGCATCAAGGTGGAACCCATTACCACCACTAGCGAGTA
AAGGGAGTAAAGGGAGGGTGAAACGAAGGAAATGATAAAGTTGAGAAATGATAAT
GGGTGAATGAACGCAAATCAGAAGCTTCGGCAGCTTTACTTGGCCTGTGTAAGCCTA
CGCCTTGGTTAAATTAAATTAATTTAAATTATATATTATTTATTATACGTGGAATCTG
TAAACTGCATTCCCCGATTTATGCCCATAGTGCAAATTGATTGAATTTGGCCCGAGA
AAACCGCATTAGGCATTGCATCCACATATTGTAAACGAGTTACTTTCCAACTACATG
ATATATCACATTTCAAATTATAATTATGTGTAAATAAACTAGATATATACGTTTTGA
AGGTTTCCTCCAAATCTTTGCGTTTTCACCTCGAACAATTCCTACACTTGATATCCAA
TCGAATTTACAAATAAGCTTTAACCACGTTCTGTTTATCATATCAAAAGTGTACATTG
CAATCTATTGTAGTTAACCAAGGTGTAGGCTGTCGCATTAGCCAGTTTTACTCTAGTT
TCCTCCACGCGATCTTCGCCACCTAGTTTCTATTCCTAGCGTACTTACTGAATCAAAA
TTAAAGTATTATTATGTTATAGTCCATCAGTCTAACTAACTAACTAAATAGCGACCG
ATCTCTTTAGTTGTAAGCTACAGCGAAGGTGGAAGCAGCAAACCCGGAACATTCCA
CAAACCATTCGAAATCTGTTTCGAATGCAACTTCAAACTTCAATTGAAACCGAATCA
GTCTAGAGGATCTTTGTGAAGGAACCTTACTTCTGTGGTGTGACATAATTGGACAAA
CTACCTACAGAGATTTAAAGCTCTAAGGTAAATATAAAATTTTTAAGTGTATAATGT
GTTAAACTACTGATTCTAATTGTTTGTGTATTTTAGATTCCAACCTATGGAACTGATG
AATGGGAGCAGTGGTGGAATGCCTTTAATGAGGAAAACCTGTTTTGCTCAGAAGAA
ATGCCATCTAGTGATGATGAGGCTACTGCTGACTCTCAACATTCTACTCCTCCAAAA
AAGAAGAGAAAGGTAGAAGACCCCAAGGACTTTCCTTCAGAATTGCTAAGTTTTTTG
AGTCATGCTGTGTTTAGTAATAGAACTCTTGCTTGCTTTGCTATTTACACCACAAAGG
AAAAAGCTGCACTGCTATACAAGAAAATTATGGAAAAATATTTGATGTATAGTGCCT
TGACTAGAGATCATAATCAGCCATACCACATTTGTAGAGGTTTTACTTGCTTTAAAA
AACCTCCCACACCTCCCCCTGAACCTGAAACATAAAATGAATGCAATTGTTGTTGTT
AACTTGTTTATTGCAGCTTATAATGGTTACAAATAAAGCAATAGCATCACAAATTTC
ACAAATAAAGCATTTTTTTCACTGCATTCTAGTTGTGGTTTGTCCAAACTCATCAATG
TNNCTTATCATGTCTGGATCCACTAGAAGGCC

UAS ANTP (5,483 bases)

CAAGAAGAGAACTCTGAATAGGGAATTGGGAATTCAGTTTGATAGGAGTCGTAAAG
AAATCAACATCACACAACAAAGCACTCGAGGACCCGTCCGTGTTCCGTTGACTTAGC
CCACGTGCGACTGTATGTGTGTGTGTGCGAGAGGGATCCCAGCAGCATAACGGTATT
CGGTGCCCCGTATTCCGTAGTCGCTATAGCGGCCAATATCTTGGGGCTGGGCTCTCG
GAGCTGAACTGAACTGAACTAAACTGAACTGAACTATTCGCATTCGTATTCGCTCGC
TCTCCGTCGTCGCGACCGCTCTCTTATCCCAATTGTCAGCCCGCTAAGATCATAAAG
GCCGTAAAAATAATCATAACAATAATCGTAAAAAATTAGAACCGCAATCAGTCGCA
CACTGTCCATCGCATGGCGCAGATCGTCATAAACCCATTATTATAATAATAACCGTA
ATCGTAACCGTAATCGTAATTGTAATCGCAGGCGCCATCGCATAAACAGCAAACAA

AAACACCGTCAGAGAAATTGGAATTGAACTTCGCGCGCGTTTCTTTTTATGATGTTC
GAGTGTCTGGCAGGTGTCTGGATTCAAATTAAAATTCATCCACATATTGTGCAATAC
TACTAATGGATCTGGGCTGACTGTGCTGCAAATGAGCTAGAGTTCAAATTTATCAAG
CCAAATCCTACTACCTAAGTGTTTATCTATTCTTAAAAACGCATCGTGCACCGAAAA
CTCGAATCTGTACAACAACGTTTTCTAGTCCCCCGTAAATCGAGCAATATCTACACA
CTAGCAACAAACAAAAGCTGAAGCTTTCAACTCAAACCACAACAAATTCAACAGAA
ATAAAACGTAGACAGCCAAAATAACACAAAAAAAAAAATAAATAATAAAAAACAAA
ATACATAAAACGGAAAAGAATAAGGAGCGGAAAAAGTTTCGATTCCACTGCGGGCA
AACTTTTTATTTTGGCGCACGTGCAAGCTACCGTTAGCTGAGAATTCCAAAAGCCAG
CAGGATTGAAGGATAAGCGCACGTTATTGGATTTAAGAGTGCAATTTTGGATTAGCC
CAGCTGTAAATGGACAACAAAGGTGGATTACCTACATGGATAAGGACAATTTAGCC
CCGCAACGCAAAAGAGTTTCAAAATCAAAATTGAGGAATACCAACTAGAGGATAAG
GCTACTTAAGGATCAAAAAACACCAAGGAGACGAGATTTTCTACCAAATCGAGAGA
CGAGGGGCAGGTTAATTTCGTCATTTTTGGCCAAGACAGCAAATAGAGGAACAGCA
AAGCGAAAATCATTTTATACCTCACACAACAACTACACACTAACTAAGATTAGGCTA
CGCAACTGTACATTGTACTTAAGTGTTCAAAGTATATTTAGTTTACTTTGTATATAAG
AAAAGTAGCTAAAAGCACGCGGACAGGGAGGCAGGAGCACCACAGTCACTAGCCA
CTAAGCAGAGTCACAGTCACGATCACGTTCACTCCAGGATCAGGACTCGGGGCGGG
ATCAGCAGACGCTGAGGAAGCTGCCACGATGACGATGAGTACAAACAACTGCGAGA
GCATGACCTCGTACTTCACCAACTCGTACATGGGGGCGGACATGCATCATGGGCACT
ACCCGGGCAACGGGGTCACCGACCTGGACGCCCAGCAGATGCACCACTACAGCCAG
AACGCGAATCACCAGGGCAACATGCCCTACCCGCGCTTTCCACCCTACGACCGCATG
CCCTACTACAACGGCCAGGGGATGGACCAGCAGCAGCAGCACCAGGTCTACTCCCG
CCCGGACAGCCCCTCCAGCCAGGTGGGCGGGGTCATGCCCCAGGCGCAGACCAACG
GTCAGTTGGGTGTTCCCCAGCAGCAACAGCAGCAGCAGCAACAGCCCTCGCAGAAC
CAGCAGCAACAGCAGGCGCAGCAGGCCCCACAGCAACTGCAGCAGCAGCTGCCGC
AGGTGACGCAACAGGTGACACATCCGCAGCAGCAACAACAGCAGCCCGTCGTCTAC
GCCAGCTGCAAGTTGCAAGCGGCCGTTGGTGGACTGGGTATGGTTCCCGAGGGCGG
ATCGCCTCCGCTGGTGGATCAAATGTCCGGTCACCACATGAACGCCCAGATGACGCT
GCCCCATCACATGGGACATCCGCAGGCGCAGTTGGGCTATACGGACGTTGGAGTTC
CCGACGTGACAGAGGTCCATCAGAACCATCACAACATGGGCATGTACCAGCAGCAG
TCGGGAGTTCCGCCGGTGGGTGCCCCACCTCAGGGCATGATGCACCAGGGCCAGGG
TCCTCCACAGATGCACCAGGGACATCCTGGCCAACACACGCCTCCTTCCCAAAACCC
GAACTCGCAGTCCTCGGGGATGCCGTCTCCACTGTATCCCTGGATGCGAAGTCAGTT
TGGTAAGTGTCAAGAACGCAAACGCGGAAGGCAGACATACACCCGGTACCAGACTC
TAGAGCTAGAGAAGGAGTTTCACTTCAATCGCTACTTGACCCGTCGGCGAAGGATCG
AGATCGCCCACGCCCTGTGCCTCACGGAGCGCCAGATAAAGATTTGGTTCCAGAATC
GGCGCATGAAGTGGAAGAAGGAGAACAAGACGAAGGGCGAGCCGGGATCCGGAGG
CGAAGGCGACGAGATAACACCACCCAACAGTCCGCAGTAGGATCGACGGAGTCTAC
CCACTTAAATGAAATTTCTATCTAAATACAATTTACGTTAGTTCGGAGAGCGCAAAT
GAATTTACTTCGATCCCAGAGGACTATCTAATAACTATCCAATCCGTTGAACTTCGC
GTGAACAAACCTAAACTAAACTAAACAAAGAGCAGAGCTGAGAACTCTACCTACAA
CTTAGTTAATTGTTATTATTTTCTACTTATTATTTAATTGTACACGAAAGGCAAGTGG
GGAAAGCGAAATAAGATTAACGTAAAGATAGCGATTACGATAAAGATACAAGTAA
AGCGTAAAACTCAAACAAAACCAACTCATGTGACCTCAGATCTAAATAAGCTATATT
TAACTATAATGCATATATATATACACATAAATATATGGATAACTATAAATGATACCA

AGTAAAGCTAAAGGCAAGGAGTTATATATAAATAAATATATATGAAGCATATATAA
TGTAACATTAGATCTACGCGTCATAAGTACTATACGATTAACTTATATATACACCCC
AGCATAAACCCTAAAACTAAACCTAAACATTAAACTAAATCAATGTTTGTAGCAATC
CTAGCGCAAAAATATAAAATAAAATCCAATAAATAAAAATAAAAAACAAATGGCGT
CAAAATCCATTGCATGTTGGTTCATAAAACCTATACATTTTTCATAACTCTGAACATG
ATAACAGAAAACTTTGACCTAAGTGAATGTCGCACTTTTAGACAAAGAAATACCAA
AACTACGAAAGAAGCGTTGCTTAAAGTGAAATTAACGTTTTACACATACAATAAGA
GTAAAACCTATAAACTGGCAGATGCTTAACTATAAATAGAAAGAACTCGCAAGAGA
TTGGCCAACTTAAAAAAAAATAGATGTATATTTCTACGACAATTCAACTTTCAAAAA
CTCGCAAGTGGATATTTATGCTTAACCAATTTGAGAGTTCCCCTTTTCTTTGGCTTCA
ATACCCATTATATCTGTATTTTTATTTGTTTGTAATTTCTTGTGCAATTTTTAGTTCTT
GCAAAAAAACAAAATTCGAATTAGGTCGAAAAGGATATAAAGTATACCGAATTACA
AAAAAATATGAATTGGCAAGTAAGGAGGAAAAATAAAAATTGTTAACAGAGAATC
ATATGTATAAATAATGAATTCTAACAAAACCCGTGTAAAGTAGTAAATTGAAATGC
ATTATTATACGAGAATGTAGGGAATCCATTTTGAAAGAGCAACCGAACGATTTAAAT
ATGAATATTTCCCTAACAACTATATTAATGTATGTGTACGTAACTTAAAATCATTTTC
CACGCCATCTGTGGAAATCCATATACCAAAGTCAAAGGAACGAAGGAGAAAGAAA
AAGGAGGACGGAATGGCAAACTATAAAGTATCATATGTTTATATGTAGATATATAT
ATTTAAACAAGCCTAATACAAAACATGTAACTTTATAGAGCGTTTCGTTTGTAAATT
CCCCAGAAATCCCATTTACCTCCCCAGCCCGAATCCCAATCCCAGGTGAAAAGACTT
GTGATTTGCAATAGAACCGAATAGTCAAGAAAAAAAATCTTTACGAAGTATTGGCT
AAGCAACATTGAGAGCAAAATTCAACTCAATCCAGATACGTAACTTTCGGCCTCATT
CTGTAAGAAACTAATTATTTAAGTTTCAATTATGAAAATTATTAAAAAAAAAACGAA
CAAAAAAAAAAACGTAAATATAAAAAAGTAGAAATTCACAAGAAACAAAATTCGA
AAGCGTTGATTAAAATATATATATATGTGAAACAAAAACAAAAAACCTTAAACAAA
CCCAAACATTTGTGCGAAATTGGAACGAAATGTGGAAGCTAAATAATTTGTTGTAAA
TAAAATTTAAACTTTAGTGTAAAGAAGAGAACGGAGAATGCAGTGAAAGGGCTGCA
AAGCGGAAAGCATAAAGAAAATTATAACTAAATTACAAATGAGATTTTGTTTTTATT
TGTAATATTTATAATAAAAATATAGTATTTAAAAATTTGAATTCGTTAACAGATCTT
GCGGCCGCGCTCGAGGGTACCTCTAGAGGATCTTTGTGAAGGAACCTTACTTCTGTG
GTGTGACATAATTGGACAAACTACCTACAGAGATTTAAAGCTCTAAGGTAAATATA
AAATTTTTAAGTGTATAATGTGTTAAACTACTGATTCTAATTGTTTGTGTATTTTAGA
TTCCAACCTATGGAACTGATGAATGGGAGCAGTGGTGGAATGCCTTTAATGAGGAA
AACCTGTTTTGCTCAGAAGAAATGCCATCTAGTGATGATGAGGCTACTGCTGACTCT
CAACATTCTACTCCTCCAAAAAAGAAGAGAAAGGTAGAAGACCCCAAGGACTTTCC
TTCAGAATTGCTAAGTTTTTTGAGTCATGCTGTGTTTAGTAATAGAACTCTTGCTTGC
TTTGCTATTTACACCACAAAGGAAAAAGCTGCACTGCTATACAAGAAAATTATGGA
AAAATATTTGATGTATAGTGCCTTGACTAGAGATCATAATCAGCCATACCACATTTG
TAGAGGTTTTACTTGCTTTAAAAAACCTCCCACACCTCCCCCTGAACCTGAAACATA
AAATGAATGCAATTGTTGTTGTTAACTTGTTTATTGCAGCTTATAATGGTTACAAATA
AAGCAATAGCATCACAAATTTCACAAATAAAGCATTTTTTTCACTGCATTCTAGTTG
TGGTTTGTCCAAACTCATCAATGTNNCTTATCATGTCTGGATCCACTAGAAGGCC

UAS EY (3,482 bases)

CAAGAAGAGAACTCTGAATAGGGAATTGGGAATTCGTTAACAGATCTTGCGGCCGC
GCTCGAGAAGCTTTCATGAGCAGTGCATGTAATAAAAACTGAGATCCAACTATGTTT
ACATTGCAACCAACTCCAACTGCTATAGGCACCGTGGTTCCCCCATGGTCAGCGGGA
ACATTGATAGAGCGCCTGCCGTCTTTAGAAGACATGGCTCACAAGGGTCACAGTGG
AGTAAATCAGCTGGGTGGCGTTTTTGTTGGAGGAAGGCCTTTGCCAGATTCAACACG
GCAAAAAATTGTCGAACTGGCACATTCTGGAGCTCGGCCATGTGATATTTCTCGAAT
TCTGCAAGTATCAAATGGATGTGTGAGCAAAATTCTCGGGAGGTATTATGAAACAG
GAAGCATACGACCACGTGCTATCGGAGGATCCAAGCCACGTGTGGCCACAGCCGAA
GTCGTTAGCAAAATTTCGCAGTACAAACGCGAGTGTCCTAGCATATTTGCTTGGGAA
ATTCGGGATAGATTACTTCAGGAGAACGTTTGTACTAACGATAATATACCAAGTGTG
TCCTCAATAAACCGTGTATTGAGAAACTTGGCTGCGCAAAAGGAGCAGCAAAGCAC
GGGATCCGGGAGCTCCAGCACATCCGCCGGCAACTCAATCAGCGCAAAAGTGTCTG
TCAGCATCGGTGGCAACGTGAGCAATGTGGCAAGCGGATCGAGAGGCACGTTGAGC
TCTTCCACCGATCTTATGCAGACAGCCACTCCTCTTAACTCTTCGGAAAGCGGTGGC
GCAACGAACTCCGGGGAGGGTAGTGAACAGGAGGCGATTTACGAGAAGCTTCGGCT
GTTAAATACTCAGCACGCTGCAGGACCAGGACCACTGGAGCCTGCCAGAGCAGCGC
CCTTGGTAGGTCAATCACCCAACCACCTAGGAACCCGATCCAGCCACCCCCAGCTGG
TGCACGGTAACCATCAGGCACTACAGCAGCATCAACAGCAGAGCTGGCCGCCCCGT
CACTATTCCGGATCTTGGTACCCCACCTCTCTTAGCGAAATACCCATCTCATCGCTCC
CAATATCGCATCCGTTACGGCGTATGCATCAGGACCTTCACTTGCTCACTCACTGAG
TCCACCCAACGACATCAAAAGCCTGGCCAGTATCGGTCACCAGAGAAACTGCCCCG
TTGCAACGGAGGACATACATTTAAAAAAAGAACTTGATGGTCATCAGTCCGATGAA
ACGGGCTCCGGTGAAGGTGAAAACTCCAATGGTGGCGCTTCAAATATAGGAAACAC
TGAGGATGATCAAGCTCGGCTCATACTAAAAAGAAAGTTGCAACGCAATCGAACAT
CTTTCACGAACGACCAGATAGACAGTCTTGAAAAAGAGTTTGAACGAACACACTAT
CCAGATGTTTTTGCCCGCGAACGTTTGGCTGGAAAGATTGGGTTGCCAGAGGCAAGA
ATTCAGGTTTGGTTCTCAAACCGTCGAGCAAAATGGCGTCGCGAGGAGAAGCTGCG
AAACCAGCGAAGAACACCAAATTCCACAGGAGCTAGTGCAACTTCTTCCTCTACATC
GGCAACCGCCTCTTTGACTGACAGCCCTAACAGCCTAAGTGCTTGTTCCTCGCTGCT
GTCCGGATCAGCTGGGGGTCCCTCAGTCAGTACCATTAATGGCTTATCGTCTCCAAG
CACATTGTCTACTAATGTCAATGCTCCAACGCTTGGCGCTGGGATCGATAGCTCTGA
AAGCCCAACACCAATCCCGCACATTCGGCCTAGCTGCACCTCTGACAATGACAATG
GTCGTCAAAGTGAAGATTGCAGAAGAGTTTGTTCTCCATGCCCACTTGGCGTTGGCG
GGCATCAAAATACTCATCATATCCAGAGCAATGGTCACGCCCAAGGTCATGCACTTG
TTCCTGCCATTTCGCCACGACTCAATTTTAATAGTGGTAGTTTCGGCGCGATGTACTC
CAACATGCATCATACGGCGTTATCCATGAGCGATTCATATGGGGCGGTTACGCCGAT
TCCGAGCTTTAACCACTCAGCTGTCGGTCCGCTGGCTCCGCCATCGCCAATACCGCA
ACAGGGCGATCTTACCCCTTCCTCGTTATATCCGTGCCACATGACCCTACGACCCCC
TCCGATGGCTCCCGCTCACCATCACATCGTGCCGGGTGACGGTGGCAGACCTGCGGG
CGTTGGCCTAGGCAGTGGCCAATCTGCGAATTTGGGAGCAAGCTGCAGCGGATCGG
GATACGAAGTGCTATCTGCCTACGCGTTGCCACCGCCCCCTATGGCGTCGAGCTCTG
CTGCTGATTCAAGCTTCTCAGCCGCGTCCAGTGCCAGCGCTAATGTGACCCCACATC
ACACCATAGCCCAAGAATCATGCCCCTCTCCGTGTTCAAGCGCGAGCCACTTTGGAG
TTGCTCACAGTTCTGGGTTTTCGTCCGACCCGATTTCACCGGCTGTATCTTCGTATGC
ACATATGAGCTACAATTACGCGTCGTCCGCTAACACCATGACGCCTTCCTCCGCCAG
CGGCACATCAGCACACGTGGCCCCGGGAAAACAACAGTTCTTCGCCTCCTGTTTCTA

CTCACCGTGGGTCTAGGAACAGACTGGCGATTTGAGCAGAGAAGCACTGCGAAAGG
ACTATTTACATAGTTGAATGTATATCTAAAGGAGGCCATAATAAATCGAATTTACAT
ATCTCTTGAAAAATAATGGAGGTTGTAGAAAAATACATTTGTATGTCTAGAGGATCT
TTGTGAAGGAACCTTACTTCTGTGGTGTGACATAATTGGACAAACTACCTACAGAGA
TTTAAAGCTCTAAGGTAAATATAAAATTTTTAAGTGTATAATGTGTTAAACTACTGA
TTCTAATTGTTTGTGTATTTTAGATTCCAACCTATGGAACTGATGAATGGGAGCAGT
GGTGGAATGCCTTTAATGAGGAAAACCTGTTTTGCTCAGAAGAAATGCCATCTAGTG
ATGATGAGGCTACTGCTGACTCTCAACATTCTACTCCTCCAAAAAAGAAGAGAAAG
GTAGAAGACCCCAAGGACTTTCCTTCAGAATTGCTAAGTTTTTTGAGTCATGCTGTG
TTTAGTAATAGAACTCTTGCTTGCTTTGCTATTTACACCACAAAGGAAAAAGCTGCA
CTGCTATACAAGAAAATTATGGAAAAATATTTGATGTATAGTGCCTTGACTAGAGAT
CATAATCAGCCATACCACATTTGTAGAGGTTTTACTTGCTTTAAAAAACCTCCCACA
CCTCCCCCTGAACCTGAAACATAAAATGAATGCAATTGTTGTTGTTAACTTGTTTATT
GCAGCTTATAATGGTTACAAATAAAGCAATAGCATCACAAATTTCACAAATAAAGC
ATTTTTTTCACTGCATTCTAGTTGTGGTTTGTCCAAACTCATCAATGTNNCTTATCAT
GTCTGGATCCACTAGAAGGCC

UAS ODD (2,742 bases)

CAAGAAGAGAACTCTGAATAGGGAATTGGGAATTCAGTTTGTGTCGAGACGTTGTC
GGTGGCACGTCGCATCCAAAAGAACGCTCAGTCAGCTGGGAAATATACTGAGAAAA
GATACATTCGTAGATACAAGTGCTAAGCCAAAGTGAACCGTGCAGTTCGCGCAACT
AAACAATTTTAAGCCAAATAAAACTACACAAGGCCAACAAAGACAGTATAATGTCT
TCCACATCGGCCTCACCCATCAGCAACATAACCGTGGATGACGAGCTCAACTTAAGC
AGAGAACAAGACTTTGCTGAAGACGATTTCATAGTGATCAAGGAGGAGCGCGAGAC
AAGTCTCTCCCCCATGCTGACGCCCCCGCACACGCCCACCGAGGAGCCGCTGAGGA
GAGTGCATCCGGCGATAAGCGAGGAGGCGGTGGCCACCCAGCTGCACATGCGACAC
ATGGCCCACTACCAGCAGCAGCAACAACAGCAGCAACAGCAGCAGCAGCACCGCCT
GTGGCTGCAGATGCAGCAGCAGCAGCAACAACATCAGGCTCCACAGCAGTATCCAG
TTTATCCCACAGCCAGCGCCGATCCCGTGGCCGTGCACCAGCAGTTGATGAACCACT
GGATCCGCAACGCAGCCATCTACCAGCAGCAACAGCAGCAGCAGCAACATCCGCAC
CATCATCACCACCACGGCCATCCGCACCACCCTCACCCACATCCGCATCATGTGCGT
CCCTATCCCGCTGGCCTCCATAGTCTGCATGCCGCGGTCGTGGGTCGCCACTTCGGA
GCCATGCCCACCCTGAAACTGGGTGGTGCCGGTGGAGCGAGTGGTGTACCCAGCTG
CGCAACTGGCAGCAGTCGGCCAAAGAAGCAGTTCATCTGCAAGTACTGCAACCGGC
AGTTCACCAAGTCGTACAATCTGCTCATCCATGAGAGAACCCACACGGACGAGAGG
CCTTACTCCTGCGACATCTGCGGCAAGGCCTTCCGGCGACAGGATCATCTGCGGGAC
CACCGTTACATCCACTCCAAGGACAAGCCCTTCAAGTGCAGCGATTGCGGCAAGGG
TTTCTGCCAGCGCGCACCCTGGCCGTGCACAAGGTCACCCATCTGGAGGAGGGTCCG
CACAAGTGTCCCATCTGCCAGCGCAGCTTCAACCAGCGGGCCAACCTCAAGAGTCA
CCTCCAGAGCCACAGCGAGCAGAGCACCAAGGAGGTGGTGGTGACCACCTCACCAG
CCACTTCACATTCCGTGCCAAACCAGGCATTGAGTTCGCCTCAACCTGAGAATTTGG
TACAGCATCTGCCCGTCCTGGATCTATCCTCGTCATCCTCGAGCTCAGAGAAACCCA
AGCGGATGCTGGGCTTCACCATCGATGAGATCATGAGCAGATAGATTGAAGGTCCA
CCGAAACCCGGTATCCGGTTTCTTTTGAGACATTCTAAAAGAGATTCAGAGAGCTGG

ACGTCCGCCAGGCATGGGAAATGCCTGGAATGGAAAACCAAGCGAGTCCAGCCCGC
ATTCTGGCCAAAGGTGCAAGTGGAAAATCCTCGCCAGCAACTCGTAATCGCAACCC
AGTGAGCAGAAGAGTCATCCTCCGTGCAATCGGTTCTATGTAGCAGATCCCAATTGC
GTTAAACAAATGCCAAAGTCAAATAGCAGAGGAAAACTGATGAAAAATCCAATGAA
AAGTATTGCTAGCACAAGCCTCCTACAAATCAATTCCAAAACACACACACACACACAA
CTGTAAACTATTAACATAAATAAATAGCACATAATTTATATTGTAATCTAACTATTA
TTGTATGTACCTTATAGTCGTAGTAGCCCATTCAGTTCGTAGTGCTTATGGAGTAAAC
CAAATGTTATGTTACAATAGCTCAATCAAACAAACCTCAAACCCGCTTTGGTTATTG
AAAGACTGTTTTAATTATTATATTTAAATAGTACTTGAATAAATCAATCGATCGATT
GTGTAATGTTGAATTCGTTAACAGATCTTGCGGCCGCGCTCGAGGGTACCTCTAGAG
GATCTTTGTGAAGGAACCTTACTTCTGTGGTGTGACATAATTGGACAAACTACCTAC
AGAGATTTAAAGCTCTAAGGTAAATATAAAATTTTTAAGTGTATAATGTGTTAAACT
ACTGATTCTAATTGTTTGTGTATTTTAGATTCCAACCTATGGAACTGATGAATGGGA
GCAGTGGTGGAATGCCTTTAATGAGGAAAACCTGTTTTGCTCAGAAGAAATGCCATC
TAGTGATGATGAGGCTACTGCTGACTCTCAACATTCTACTCCTCCAAAAAAGAAGAG
AAAGGTAGAAGACCCCAAGGACTTTCCTTCAGAATTGCTAAGTTTTTTGAGTCATGC
TGTGTTTAGTAATAGAACTCTTGCTTGCTTTGCTATTTACACCACAAAGGAAAAAGC
TGCACTGCTATACAAGAAAATTATGGAAAAATATTTGATGTATAGTGCCTTGACTAG
AGATCATAATCAGCCATACCACATTTGTAGAGGTTTTACTTGCTTTAAAAAACCTCC
CACACCTCCCCCTGAACCTGAAACATAAAATGAATGCAATTGTTGTTGTTAACTTGT
TTATTGCAGCTTATAATGGTTACAAATAAAGCAATAGCATCACAAATTTCACAAATA
AAGCATTTTTTTCACTGCATTCTAGTTGTGGTTTGTCCAAACTCATCAATGTNNCTTA
TCATGTCTGGATCCACTAGAAGGCC

UAS FOXO (4,499 bases)

CAAGAAGAGAACTCTGAATAGGGAATTGGGAATTCTTCAGTTGTCAGTTCGCATCCA
ACTAGAAAGCAGTTAACGAGTAGTCTGTGTTTTTTCGCTTGCGGTTAAAAGCCACGA
GCTTAAGCAAAACCGCCGCCCAGCAACAACAAGAAACCCATTTAATGAGTGTGTGT
GTGTGTGAGCAGAAAATTATGCCAACAGAATTCTAAAAAATATATATGTATATATTT
AAATAACCACTAAAAATCAAGAAATCCGCACAAGTTTCGCTCAAAACAACAACACA
ATCATATATACTACCCATAAAAAAAAAACAAACCCATAAAAAAGTGCAAAGCAACC
AGGAAAATAAAGTAAAACAGTGCCAGTTAACAACAGCAAACGGCGATCAACAAAT
CCCACCGGCAAAATCAACAATTTTTTAAATCGCAATCAACAAGGAGCAACAAATTA
GCCAGCTATTTCCCATTGCAAACATTTGGCGGATTGATTGCGAAGCTTTCACATTCG
CTTCGATATCTTTTGGGCTGGCGAGGATTGCTTGAAGAGCCGCAAAGCTGCAACTGC
ACAACGAACTTTTCTACCCCATGATGGACGGCTACGCGCAGGAATGGCCCAGGCTG
ACCCACACAGATAACGGCCTGGCCATGGACCAGCTGGGCGGGGATCTGCCCCTGGA
CGTGGGCTTCGAGCCACAGACCCGGGCCAGATCCAACACATGGCCATGTCCGCGTC
CCGAAAACTTTGTGGAGCCCACCGACGAGTTGGACAGTACAAAGGCCAGCAATCAG
CAGTTGGCCCCAGGAGACTCACAGCAGGCTATACAGAATGCGAATGCAGCCAAGAA
GAACTCATCGCGTCGCAATGCATGGGGAAATCTATCCTATGCGGATCTCATCACGCA
TGCCATTGGATCGGCCACCGACAAACGATTGACACTGAGTCAGATTTACGAGTGGAT
GGTCCAGAATGTGCCATATTTCAAGGACAAGGGCGATTCGAATAGCAGTGCCGGAT
GGAAGAACTCCATACGTCACAATCTGTCGCTGCACAACCGCTTTATGAGGGTCCAAA

ACGAGGGCACCGGCAAGTCATCCTGGTGGATGCTCAACCCGGAGGCCAAGCCCGGC
AAGTCTGTGCGCCGCCGTGCCGCTTCCATGGAGACGTCCCGGTACGAGAAGCGGCG
CGGCAGGGCCAAGAAGCGGGTGGAGGCACTGCGTCAGGCGGGCGTGGTGGGCCTC
AACGATGCCACGCCCTCGCCCAGCAGCAGCGTCAGCGAGGGGCTGGATCACTTTCC
CGAGAGTCCGCTCCACAGTGGCGGTGGCTTCCAATTATCGCCCGATTTCCGGCAACG
CGCCTCATCCAATGCAGTTCCTGCGGACGCCTGAGCCCCATTAGGGCGCAGGATCTT
GAGCCCGACTGGGGATTCCCCGTTGACTACCAGAACACAACGATGACGCAGGCCCA
CGCCCAGGCGCTCGAGGAGCTGACGGGCACAATGGCGGATGAGCTGACGCTGTGCA
ACCAGCAGCAGCAAGGGTTCAGTGCCGCCTCGGGACTTCCCTCTCAGCCCCCGCCCC
CGCCCTATCAGCCGCCGCAGCATCAACAGGCGCAGCAGCAGCAACAGCAGCAGTCG
CCCTACGCCCTCAACGGCCCCGCCTCCGGCTACAACACGCTGCAGCCGCAGTCGCAG
TGCCTGCTGCACCGGTCCCTCAATTGCAGTTGCATGCACAATGCAAGAGATGGTCTC
TCGCCGAACTCAGTAACCACAACAATGTCGCCCGCCTATCCAAACAGCGAGCCCTC
ATCGGACTCCCTGAACACGTACAGCAACGTGGTGCTCGATGGTCCGGCGGACACTG
CGGCACTGATGGTGCAGCAGCAGCAGCAGCAGCAGCAACAGCAACAGCTGTCCGCC
AGCTTGGAAGATAATAACTGCGCCTCTACTTTGATAGGACAATGCCTGGAGGTGCTC
AATAACGAGGCGCAGCCGATAGACGAATTTAATCTGGAGAACTTTCCCGTGGGCAA
TCTCGAGTGCAATGTCGAGGAGCTGCTGCAGCAGGAGATGAGCTACGGCGGCCTGC
TGGACATCAATATACCGCTGGCCACGGTCAACACGAACCTGGTCAACAGCAGCAGT
GGGCCCCTGAGCATCAGCAACATTAGCAACCTCAGCAACATAAGCAGCAATTCCGG
CAGCAGTCTCAGTCTGAATCAGTTGCAGGCTCAGCTGCAACAGCAGCAGCAGCAGC
AGCAGGCGCAGCAACAGCAGCAGGCGCAGCAGCAACAACAGCAGCATCAGCAGCA
CCAGCAACAGTTGCTGCTAAATAATAACAACAACAGCAGCAGCAGCCTGGAATTGG
CAACACAAACGGCTACCACAAATCTGAATGCTCGGGTTCAGTACTCACAGCCCAGC
GTGGTGACCTCGCCACCATCCTGGGTGCACTAGATCCACTGTGGGTCGATCGTAATC
CAAATCGCAAGGATGATCGTTATAGCCGGTAGTCGTCGTTGTTGTGTGTGTGCGTGT
GTGTTGTTTGTGTCGCCATTGGAATGCTTTAAACGCTAATTCTAAGAACCGTAGTGT
AAAGAATTGTAAGCAGCCGCAGCTGGTTGGGAAACTGGAAACGAGGATTCGGATGC
GGAGTCGATGTCTTTATCGGGTATGCAGCAGCTGCAGGATCAGTGAGCCGAAAGCG
GGAGCCGCAGCTGGACCGTCACTAAAAAGTTTACAAAGATAAATCTGGTCGCTTCTA
ATCTAACATACATACATACAATACATATACATAGATATATAACTACAATTATATAGT
TATGCATTACACATAGAAACGAAGCATACAAATAATACCAATTAATCACCAATAATT
AGATGCTACAATTTGTGAAATTCTCTGAGTGGCAGAGAGCTGAGAAAACATCTTTGG
CCACCAATGAAGGGAAATTTGAATTGAATTGAAAACACGGTTTAGTTATTAGGTTCA
ACTTTGTACATATTAATTTTAATAACCTTGACGTAATGAAACCCACAAATAATAATA
ATACTTCTAATGATAATAAACAACAAGAAACAGCGTAAAACGTAAACTAAATGTTC
GACAACTAAATGGAAAAATATTTGCTAGCCAGCAGTCATCGAGGAAAATAGAAACA
AGAAAATGGCAACAGAAAATATAAATGAAAAACATAAGAAATTAGCACAATTATGT
AATAATTAATGCTTAATTAATGTATTTAATTGAAGGTCTAATTGTAATTGTATATTTT
TTTCTATAAAGAGCAAAGAAAACCCTTAAAGAAGAATACAACTTGTGTAAAAATTA
TGCTTAAGATTCGCAATGATATACGTAATAAATTCTCCGATTAGTTAGCACACATCA
AATCAGCTCGGCTTTAAAAATAAAACCCTTTGTTACATAATTATGCCAACATCCATA
TGTAGATCGTAGACCTATAACAAAGATTTGAGTGTAATACAATTAATTTCATATATT
ATTAGTAAATGTAAATGTTGATAGGCAAAAGAAAAAAACAAAATTATAACAGAATA
GCCACAAAACTCAAAAACCCAGCAGAACTAAAAGCATAAAGTAATAAGCAAATAA
ATGCGCATCATTGTGTACTATTATGGGCTAAATGTTAATTATTAACTAGATTGTACA

ATTCAAATGTAGTTCATACTATTTAAAATGCAAAAACGGAGTGAAGATGAATAAAC
TGATGATTCAATATAAGAATTGAATTCGTTAACAGATCTTGCGGCCGCGCTCGAGGG
TACCTCTAGAGGATCTTTGTGAAGGAACCTTACTTCTGTGGTGTGACATAATTGGAC
AAACTACCTACAGAGATTTAAAGCTCTAAGGTAAATATAAAATTTTTAAGTGTATAA
TGTGTTAAACTACTGATTCTAATTGTTTGTGTATTTTAGATTCCAACCTATGGAACTG
ATGAATGGGAGCAGTGGTGGAATGCCTTTAATGAGGAAAACCTGTTTTGCTCAGAA
GAAATGCCATCTAGTGATGATGAGGCTACTGCTGACTCTCAACATTCTACTCCTCCA
AAAAAGAAGAGAAAGGTAGAAGACCCCAAGGACTTTCCTTCAGAATTGCTAAGTTT
TTTGAGTCATGCTGTGTTTAGTAATAGAACTCTTGCTTGCTTTGCTATTTACACCACA
AAGGAAAAAGCTGCACTGCTATACAAGAAAATTATGGAAAAATATTTGATGTATAG
TGCCTTGACTAGAGATCATAATCAGCCATACCACATTTGTAGAGGTTTTACTTGCTTT
AAAAAACCTCCCACACCTCCCCCTGAACCTGAAACATAAAATGAATGCAATTGTTGT
TGTTAACTTGTTTATTGCAGCTTATAATGGTTACAAATAAAGCAATAGCATCACAAA
TTTCACAAATAAAGCATTTTTTTCACTGCATTCTAGTTGTGGTTTGTCCAAACTCATC
AATGTNNCTTATCATGTCTGGATCCACTAGAAGGCC

UAS SQZ (2,906 bases)

CAAGAAGAGAACTCTGAATAGGGAATTGGGAATTCATGGCCGAACTGCCGACGGCG
CCGAACGGCGTCCCCAGCGGCGATTATCTGCACCGCTCCATCGATCAGCTGCGTTCG
CTGGGTCATCTGACCACCGCCCAATTGGTTCACGACTACAAGCCCTTCAACATTAGC
GAATTCCGGCAGAATGTCGCTGAGCGACTGGACTACTCGCTGAAGAACGGCCTGGT
GCAGCACCAACAGCAAATGGTCATGGAGCAGCAGCCACATCCCGATCAGCAGCAGC
AGCAGCATCTGCATCACCCGCAACAGCAGCAGCACCCGCCGCAGCTGAAGGTCAGC
TACAGTGCGCCCAACTCGCCGCCCACTCCACACGAGCAGCAGGAACAGAAGTACGA
CCCGAATCGATCGCCGCCGCGTCAGCAGATGAGCAGCGCTAGCGGCAGTGGCAGCA
ACGGCTCCTCGCCTGAGGAGGAAAGCCGACGGGGAGACGGTGATCAGGCCAAGCCC
TACAAGTGTGGCTCGTGCAGCAAGTCCTTTGCCAACTCCTCGTACCTGTCGCAGCAC
ACGCGTATCCACCTGGGGATCAAGCCGTACCGCTGCGAGATATGTCAGCGCAAGTT
CACGCAATTGTCGCATCTCCAGCAGCACATCCGTACGCACACGGGTGACAAACCGT
ACAAATGCCGGCACGCCGGCTGCCCGAAGGCCTTCTCGCAGCTATCCAATCTGCAGT
CACACTCGCGTTGTCATCAGACGGACAAGCCGTTCAAGTGCAACTCCTGCTACAAGT
GCTTCAGCGACGAGATGACCCTGCTGGAGCACATTCCCAAGCACAAGGACTCCAAG
CACCTGAAGACGCACATCTGCAATTTGTGTGGCAAATCGTACACGCAAGAGACCTA
CCTTCAGAAACATCTGCAGAAGCACGCAGAGAAGGCGGAGAAGCAGCAGCATCGC
CACACGGCCCAGGTGGCTGCCCACCAGCAGCACGTACCGGCGAGCGGCATCGGCTT
GAATTTGCAGCGCCAGGCCATGAACGATGTGAATGCCGCATATTGGGCCAAAATGG
GCGCAGACAGTGCGGCGGCTTCGCTGGCGGAAGCCATTCAGCAGCAGTTGCCGCAG
GCCGGCGGTCAGCCGTATGGCAACTTTGCATCCCTGCAGCAGCAGCATCAGCAACA
GCAGCAGGAACTGCTGCATCATCAGCGATTGGCAGACACGCCGGGGCATTCGCATA
GTCCTCACGAGGAGGCCGCGGGCGAGGATCTCGTCCTGCGCCAGTCAACACCACAA
CATCACCTTCAGCAACAACAGCAGCAGCAGCAACAGCAGCAGGCCCAGCAGCAACA
ACAAGCGCAGCACCAGCCATCGCCTGGACCGGGAAACTCGGCCTTCACTCCACTGT
CGGCGACAGTGGCTCCACCGCCACATCTTCAACAGCATCGCGGTCCGCCAGGATCC
GCCGCTGCCTATCTGTATCAGCAGAATGCGGCGGCAGCAGCAGCGGCTTTTCCCACA

CAGCTCATCTCGCTTCACCAGATCCGAAACTATGCCCATCAGCCAGGAGCGGCGGGT
CTCATCGCTGGCGATCATCTCGCCTTGGGCCTGAGCGCTGTTAATGCCGCCAAAGAA
AAGGCGCAATAGTACAGGCCGTCAGGTCGGGGCCAAAAGAGGCAGCAACGATGAC
ATTAGCTTAGGATCAACGTTTAAGTTGGTATGGATACAAGAGCGTGGTATTTTATTC
TCTTTGAATCCATACCAATTGAAACACTGAAAGTAAAATAAGTTACTCATAGTTTCC
AGTTGGCAGTGGCATAGGAGCTGGGATTGTGACTGGTTTTTCTTAAGCATCCTTCCA
ACTTCCAAATTCATCATGAAACTTGTATACGATTCGGCCAAAATCAACTCCATCACT
GCCCAAAACCAATAAGAAAATCAACTGCAATCCTACAGTTTCTGTGCTATTTTCAAC
TGCCATATGAAAGTAATATGTAATATCTAGATCAACTCAGGATTTAAATACGATAA
TTATGCACACTTTTAGATATCTATAAGCAATATTCAAATTAAAACCTTGAACTATAC
AAATAACTCGATTATAAATAGTTCACAGTTTTATTTCTACTAGGAGCAGTACAAAG
TACAGCGGAATTCGTTAACAGATCTTGCGGCCGCGCTCGAGGGTACCTCTAGAGGAT
CTTTGTGAAGGAACCTTACTTCTGTGGTGTGACATAATTGGACAAACTACCTACAGA
GATTTAAAGCTCTAAGGTAAATATAAAATTTTTAAGTGTATAATGTGTTAAACTACT
GATTCTAATTGTTTGTGTATTTTAGATTCCAACCTATGGAACTGATGAATGGGAGCA
GTGGTGGAATGCCTTTAATGAGGAAAACCTGTTTTGCTCAGAAGAAATGCCATCTAG
TGATGATGAGGCTACTGCTGACTCTCAACATTCTACTCCTCCAAAAAAGAAGAGAAA
GGTAGAAGACCCCAAGGACTTTCCTTCAGAATTGCTAAGTTTTTTGAGTCATGCTGT
GTTTAGTAATAGAACTCTTGCTTGCTTTGCTATTTACACCACAAAGGAAAAAGCTGC
ACTGCTATACAAGAAAATTATGGAAAAATATTTGATGTATAGTGCCTTGACTAGAGA
TCATAATCAGCCATACCACATTTGTAGAGGTTTTACTTGCTTTAAAAAACCTCCCAC
ACCTCCCCCTGAACCTGAAACATAAAATGAATGCAATTGTTGTTGTTAACTTGTTTAT
TGCAGCTTATAATGGTTACAAATAAAGCAATAGCATCACAAATTTCACAAATAAAG
CATTTTTTTCACTGCATTCTAGTTGTGGTTTGTCCAAACTCATCAATGTNNCTTATCA
TGTCTGGATCCACTAGAAGGCC

UAS AP (2,505 bases)

CAAGAAGAGAACTCTGAATAGGGAATTGGGAATTCGTTAACAGATCTTGCGGCCGC
GCTCGAGGGTACCTCTAGAGGATTACCCAAGACCAACGTAGCACCGCAGCCCTTTTA
AGGAGGCATAAGGCTCGCAAGACACAACGCACAGCCTACACAACGACAAACATAA
CACGACCGACGGACATCATCCTTGGAAATAGACCTCGTGTCATTCCAAAAGACGCA
CATGGGCGTCTGCACCGAGGAGCGCCCTGTGATGCATTGGCAGCAGAGCGCAAGAT
TTCTTGGGCCCGGCGCAAGGGAGAAAAGTCCAACACCACCTGTAGCACATCAAGGG
AGCAATCAATGTGGCAGTGCTGCAGGTGCAAATAACAATCACCCATTGTTCCGCGCG
TGCTCCTCATCCTCGTGTCCAGATATTTGTGATCATAGTACAAAGCCATTTGGCAAC
GCTTACGGCACCGAGTCATTTAGAAGCTACGAAACAGCCGATCGTGCTACCTTTGAG
GACTCAGCCGCCAAATTCTCCATCAGCCGCAGTCGAACAGACTGCACGGAGGTCAG
CGACGAGACGACGTCGGGCATATCATTCAAGACCGAACCCTTCGGACCGCCCAGCA
GTCCCGAGTCCACAAGCGATAGCAAAATAACGCGCAACCTCGACGACTGCTCCGGC
TGCGGACGTCAGATACAGGATCGCTTCTACCTCTCCGCTGTGGAAAAACGGTGGCAT
GCAAGTTGCCTACAGTGCTACGCGTGTCGGCAGCCGCTGGAACGGGAATCCTCATG
CTACTCACGTGACGGCAACATTTATTGCAAAAACGATTATTATAGTTTTTTTGGTACT
CGCCGATGCTCGCGCTGCCTGGCCTCCATCAGCTCCAACGAGCTGGTCATGCGCGCC
CGAAATCTTGTTTTTCACGTCAACTGCTTCTGCTGCACTGTCTGCCACACGCCACTGA

CAAAGGGAGACCAGTACGGCATCATCGACGCCCTCATCTACTGCAGGACCCACTAC
AGCATAGCCAGGGAGGGGGATACCGCCTCATCCAGTATGAGCGCCACCTACCCGTA
CAGCGCCCAGTTCGGCTCACCCCACAACGACTCCTCGAGCCCGCACTCGGACCCTAG
TCGGAGCATTGTTCCTACGGGCATCTTTGTGCCCGCGTCCCACGTAATCAACGGACT
GCCGCAGCCAGCTCGTCAAAAGGGCAGGCCCCGCAAGCGCAAGCCCAAGGACATCG
AGGCGTTCACCGCTAACATAGATCTCAACACTGAGTACGTGGACTTTGGCCGAGGCT
CGCACCTAAGCTCCTCGTCGCGCACCAAACGAATGCGAACCTCGTTTAAGCATCACC
AGCTGCGTACCATGAAGTCCTACTTTGCCATTAATCATAACCCTGATGCAAAGGATC
TAAAACAATTGTCACAGAAAACTGGTTTACCAAAGAGGGTCCTACAGGTCTGGTTTC
AAAATGCAAGGGCCAAATGGCGCCGCATGATGATGAAGCAGGATGGCAGCGGCCTG
CTGGAGAAGGGCGAAGGCGCTCTGGACCTCGACAGCATCTCCGTGCACAGTCCTAC
GTCGTTTATATTGGGAGGACCGAACAGCACACCGCCACTTAACTTGGACTAACGGAT
GCTCAGGAAACGCGAAAACAAAATGATAGTGACAAAGGAAATGGGCGCAGCAGTC
ACCTCCGACATCATACGTTCTTATGCCAGTTGTCTATTCGCGTGCCTCATACACAGTC
CCGGACCCGGCTTGACTACTGAATCCGGATACTTGTCTAGAGGATCTTTGTGAAGGA
ACCTTACTTCTGTGGTGTGACATAATTGGACAAACTACCTACAGAGATTTAAAGCTC
TAAGGTAAATATAAAATTTTTAAGTGTATAATGTGTTAAACTACTGATTCTAATTGTT
TGTGTATTTTAGATTCCAACCTATGGAACTGATGAATGGGAGCAGTGGTGGAATGCC
TTTAATGAGGAAAACCTGTTTTGCTCAGAAGAAATGCCATCTAGTGATGATGAGGCT
ACTGCTGACTCTCAACATTCTACTCCTCCAAAAAAGAAGAGAAAGGTAGAAGACCC
CAAGGACTTTCCTTCAGAATTGCTAAGTTTTTTGAGTCATGCTGTGTTTAGTAATAGA
ACTCTTGCTTGCTTTGCTATTTACACCACAAAGGAAAAAGCTGCACTGCTATACAAG
AAAATTATGGAAAAATATTTGATGTATAGTGCCTTGACTAGAGATCATAATCAGCCA
TACCACATTTGTAGAGGTTTTACTTGCTTTAAAAAACCTCCCACACCTCCCCCTGAAC
CTGAAACATAAAATGAATGCAATTGTTGTTGTTAACTTGTTTATTGCAGCTTATAATG
GTTACAAATAAAGCAATAGCATCACAAATTTCACAAATAAAGCATTTTTTTCACTGC
ATTCTAGTTGTGGTTTGTCCAAACTCATCAATGTNNCTTATCATGTCTGGATCCACTA
GAAGGCC

UAS AP3XHA (1,740 bases)

GAAGTTCCTATTCTTCAAAAGGTATAGGAACTTCAACCGGTACAAGTTTGTACAAAA
AAGCAGGCTAAAAAGCAGGCTTCAACATGGGCGTCTGCACCGAGGAGCGCCCTGTG
ATGCATTGGCAGCAGAGCGCAAGATTTCTTGGGCCCGGCGCAAGGGAGAAAAGTCC
AACACCACCTGTAGCACATCAAGGGAGCAATCAATGTGGCAGTGCTGCAGGTGCAA
ATAACAATCACCCATTGTTCCGCGCGTGCTCCTCATCCTCGTGTCCAGATATTTGTGA
TCATAGTACAAAGCCATTTGGCAACGCTTACGGCACCGAGTCATTTAGAAGCTACGA
AACAGCCGATCGTGCTACCTTTGAGGACTCAGCCGCCAAATTCTCCATCAGCCGCAG
TCGAACAGACTGCACGGAGGTCAGCGACGAGACGACGTCGGGCATATCATTCAAGA
CCGAACCCTTCGGACCGCCCAGCAGTCCCGAGTCCACAAGCGATAGCAAAATAACG
CGCAACCTCGACGACTGCTCCGGCTGCGGACGTCAGATACAGGATCGCTTCTACCTC
TCCGCTGTGGAAAAACGGTGGCATGCAAGTTGCCTACAGTGCTACGCCTGTCGGCAG
CCGCTGGAACGGGAATCCTCATGCTACTCACGTGACGGCAACATTTATTGCAAAAAC
GATTATTATAGTTTTTTTGGTACTCGCCGATGCTCGCGCTGCCTGGCCTCCATCAGCT
CCAACGAGCTGGTCATGCGCGCCAGAAATCTTGTTTTTCACGTCAACTGCTTCTGCT

GCACTGTCTGCCACACGCCACTGACAAAGGGAGACCAGTACGGCATCATCGACGCC
CTCATCTACTGCAGGACCCACTACAGCATAGCCAGGGAGGGGGATACCGCCTCATC
CAGTATGAGCGCCACCTACCCGTACAGCGCCCAGTTCGGCTCACCCCACAACGACTC
CTCGAGCCCGCACTCGGACCCTAGTCGGAGCATTGTTCCTACGGGCATCTTTGTGCC
CGCGTCCCACGTAATCAACGGACTGCCGCAGCCAGCTCGTCAAAAGGGCAGGCCCC
GCAAGCGCAAGCCCAAGGACATCGAGGCGTTCACCGCTAACATAGATCTCAACACT
GAGTACGTGGACTTTGGCCGAGGCTCGCACCTAAGCTCCTCGTCGCGCACCAAACG
AATGCGAACCTCGTTTAAGCATCACCAGCTGCGTACCATGAAGTCCTACTTTGCCAT
TAATCATAACCCTGATGCAAAGGATCTAAAACAATTGTCACAGAAAACTGGTTTACC
AAAGAGGGTCCTACAGGTCTGGTTTCAAAATGCAAGGGCCAAATGGCGCCGCATGA
TGATGAAGCAGGATGGCAGCGGCCTGCTGGAGAAGGGCGAAGGCGCTCTGGACCTC
GACAGCATCTCCGTGCACAGTCCTACGTCGTTTATATTGGGAGGACCGAACAGCACA
CCGCCACTTAACTTGGACACCCAGCTTTCTTGTACAAAGTGGTGGTACCTCGAAGTT
CCTATTCTCTACTTAGTATAGGAACTTCCATCCTTGGAGCTCCTTCAGGAGGCGGTGC
TACTGCTGGCGCTGGTGGAGCCGGTGGACCTGCGGGGTTAATTTACCCATACGATGT
TCCTGACTATGCGGGCTATCCCTATGACGTCCCGGACTATGCAGGATCCTATCCATA
TGACGTTCCAGATTACGCTTGACGCTAGCACTAAGCGTC

UAS TTK (2,262 bases)

GAAGTTCCTATTCTTCAAAAGGTATAGGAACTTCAACCGGTACAAGTTTGTACAAAA
AAGCAGGCTAAAAAGCAGGCTTCAACATGAAGATGGCATCTCAACGCTTCTGCCTG
CGGTGGAACAACCACCAGAGCAACCTTCTGTCCGTCTTCGACCAGCTGTTGCACGCA
GAAACCTTCACAGATGTGACGCTGGCCGTCGAGGGGCAACACCTGAAGGCACACAA
GATGGTGCTATCCGCCTGCAGTCCCTACTTTAATACCCTCTTTGTAAGTCATCCGGAA
AAGCATCCGATTGTCATACTTAAGGATGTGCCCTACTCGGACATGAAGTCGTTGCTA
GACTTTATGTACAGGGGCGAGGTCTCAGTGGACCAGGAGCGACTCACTGCATTCCTG
CGCGTGGCCGAGAGCCTGCGCATCAAGGGCCTCACCGAGGTCAACGACGACAAGCC
CTCGCCGGCAGCAGCAGCTGCAGGAGCGGGTGCGACGGGCTCTGAGAGCACAGCCA
CTACACCCCAGCTGCAGCGCATCCAACCGTATTTGGTGCCCCAGCGGAATCGCTCGC
AGGCCGGCGGTCTGCTGGCCAGTGCCGCCAATGCCGGAAATACACCCACCCTGCCG
GTGCAGCCATCGCTGCTCAGCTCGGCCCTGATGCCCAAGCGAAAGAGGGGCAGACC
CCGGAAGCTATCTGGCAGCTCGAATGGCACGGGGAACGACTACGACGACTTTGATC
GCGAGAACATGATGAACGACTCCTCCGACCTGGGCAACGGCAAAATGTGCAACGAG
TCCTACTCCGGCAATGACGATGGCTCCGACGACAATCAGCCGAACGCAGGACACAC
GGATGATCTAAATGAAAGTCGCGATTCTCTGCCCTCGAAACGATCAAAGAACTCCA
AGGATCACCGCGTGGTGAGCCATCATGAAGACAACAGCACTTCCGACGGAAACGAC
AGCGATGGCGAAGGTCTGGACACATCATATATGGAACCTCAACTTATGCTGGACGA
ATATGACGAGCCCGTAGAGTTCAAGTACAACCCGCTCACCGACAACAGCTCACCCA
CGCAGGACCACACGGATGGTAGTCACCTCAACGAGCAGGCGCGCCAACAAGCCTTC
CTCATCGCTGCCCAGAGAAAACACCAGGTGGAAACAGCAGCAGCGGCGGCGGCCA
GCGGAATCAAACTTAACATTATCGGAATGGCGGCTGGTGGAGCGCAGGTGAAAAGC
ATGGTCAGCATACCCAAACTAACGCCCATTGGCAAGGTCAACGCCGCCTCCACGCC
ATTGGTCTCGCCTGCCGGCTCCTTTTCCACAGCCACGGTCAAGCCGCGCGTCCAGAA
GCGGCCCAAACTGGGCAAACAGAACGGCGATGTAAAACCGGCTGTGTTTAGCAGCC

AGGAATACCTCGACATTTACAACAGCAACGATGGATTCAAGTTGAAGGCTGCTGGT
CTGAGCGGAAGCACGCCGAACCTGAGTGCTGGATTAGGAACTCCCTCTGTCAAAAC
CAAGCTGAATCTTAGCAGCAACGTTGGCGAGGGCGAGGCGGAGGGCTCAGTGAGGG
ACTACTGCACCAAAGAGGGCGAGCATACGTACCGATGCAAAGTCTGCTCCCGCGTC
TACACGCACATTAGTAACTTCTGCCGTCACTATGTTACCTCCCACAAGCGAAATGTG
AAAGTGTACCCCTGTCCCTTCTGCTTCAAAGAGTTTACGCGCAAGGACAATATGACG
GCGCACGTGAAAATCATCCACAAGATCGAAAATCCCTCGACGGCGTTGGCCACAGT
CGCGGCAGCGAATCTAGCAGGCCAGCCACTGGGAGTTTCGGGAGCCTCGACGCCTC
CGCCGCCGGATCTGAGTGGTCAGAACTCAAATCAGTCGCTGCCAGCCACCAGCAAT
GCGCTATCCACCTCCTCCTCGTCGTCGACGTCCTCGTCCAGCGGATCCCTGGGTCCGT
TGACGACCTCCGCACCGCCAGCACCAGCAGCTGCGGCGCAGACCCAGCTTTCTTGTA
CAAAGTGGTGGTACCTCGAAGTTCCTATTCTCTACTTAGTATAGGAACTTCCATCCTT
GGAGCTCCTTCAGGAGGCGGTGCTACTGCTGGCGCTGGTGGAGCCGGTGGACCTGC
GGGGTTAATTTACCCATACGATGTTCCTGACTATGCGGGCTATCCCTATGACGTCCC
GGACTATGCAGGATCCTATCCATATGACGTTCCAGATTACGCTTGACGCTAGCACTA
AGCGTC

UAS CAD (1,614 bases)

GAAGTTCCTATTCTTCAAAAGGTATAGGAACTTCAACCGGTACAAGTTTGTACAAAA
AAGCAGGCTAAAAAGCAGGCTTCAACATGGTTTCGCACTACTACAACACACTGCCC
TACACACAAAAGCACAGTGCCGCCAATTTGGCCTATGCCTCAGCGGCGGGCCAGCC
ATGGAACTGGACGCCCAACTACCACCACACGCCGCCGAACCACCAGTTCCTGGGCG
ATGTGGACTCCTCGCATGCCGCCCACCACGCGGCCGCTGCCCACCAGATGTACTACA
ACTCCCATCACATGTTCCACTCGGCGGCGGCGGCCTCTGCCGGGGAGTGGCACTCAC
CCGCCTCGAGCACGGCGGATAACTTCGTTCAGAATGTGCCCACGTCGGCCCACCAGC
TGATGCAGCAGCACCACCATCATCACGCCCATGCCTCCAGCAGTTCCGCCAGTTCCG
GGAGCAGCAGTAGCGGTGGTGCACCGGGTGCGCCCCAGCTCAACGAGACCAACAGC
AGCATCGGCGTCGGAGGAGCAGGCGGTGGCGGTGGTGTTGGCGGGGCCACCGATGG
AGGACCAGGTTCCGCACCACCCAACCATCAGCAGCACATCGCCGAAGGTCTGCCGT
CGCCACCGATTACCGTTTCGGGCTCGGAAATCTCTAGCCCGGGGGCGCCGACTTCTG
CCTCGTCGCCGCATCATCACTTGGCACACCATTTGAGTGCCGTCGCCAACAACAACA
ACAACAACAACAACAACAATAGCCCATCCACCCATAACAACAACAACAATAAC
AATTCGGTGAGCAACAACAACAGGACATCGCCATCGAAGCCGCCATACTTCGACTG
GATGAAGAAGCCCGCCTATCCAGCACAACCACAACCAGGCAAAACCCGCACCAAGG
ATAAGTACCGCGTGGTGTACACCGACTTCCAGCGCCTGGAGCTGGAGAAGGAGTAC
TGCACCTCCCGCTACATCACCATCCGGCGGAAGAGCGAGCTCGCCCAGACGCTGTC
GCTGTCGGAGCGCCAGGTTAAGATCTGGTTCCAGAACCGCCGCGCCAAGGAGCGCA
AGCAGAACAAGAAGGGTAGCGATCCGAACGTGATGGGCGTGGGAGTGCAGCATGC
GGACTACAGCCAGCTGCTGGACGCCAAAGCGAAGCTGGAGCCGGGCCTGCACCTGT
CCCACTCGCTGGCCCACTCGATGAACCCCATGGCGGCGATGAACATTCCCGCCATGC
GCCTGCATCCCCACCTGGCCGCCCACAGCCACTCCTCGGCGGCGGTGGCGGCGCACT
CACACCAGCTGCAGCAGCAGCACAGTGCGCAGATGTCCGCTGCGGCGGCAGTGGGC
ACGCTCTCGATGACCCAGCTTTCTTGTACAAAGTGGTGGTACCTCGAAGTTCCTATTC
TCTACTTAGTATAGGAACTTCCATCCTTGGAGCTCCTTCAGGAGGCGGTGCTACTGC

TGGCGCTGGTGGAGCCGGTGGACCTGCGGGGTTAATTTACCCATACGATGTTCCTGA
CTATGCGGGCTATCCCTATGACGTCCCGGACTATGCAGGATCCTATCCATATGACGT
TCCAGATTACGCTTGACGCTAGCACTAAGCGTC

UAS SISA (900 bases)

GAAGTTCCTATTCTTCAAAAGGTATAGGAACTTCAACCGGTACAAGTTTGTACAAAA
AAGCAGGCTAAAAAGCAGGCTTCAACATGGAACGGAGTCATCTTTACTTGCCCACTC
TGAGCTACGCGGCCATGGGTCACGTATACGCACCGTATCGCGGAAGCAGTTCACCC
GCACTATCGACAGCATCGTCAACATCATCGAAGCCGGAGCAGATCGAGGAGCTGGT
GTCCCAGCAGCTGCATCATCTCAAAATGCACTACGCCGACGAGGAGCAACGCTACG
TAGACCAGATGCTCCTGGAGAATCCCATTGTTGTGGAGCGCCGTGCACCGCCGCCGC
TGAAAACGGAGCTTGCTATGGATTGCCGCGGATCGGGTTCCGGATCAGGTTCCGGTT
CTGGTTCGGATGTCAAGGATGCCCAGCGTCAGAGGGCCGAGTCGTGCCGCAAATCC
CGCTACAACAACAAGATCAAGAAGGCCAAGCTGCGCTTCCGGCACAAATTCGTCAG
CGGGCAGCTGAAGAAGAGCGCCGTCATGTTGGACACGATGCGGGATGTGATTGCCC
AGGCGGAGAGGCAGCTGCTCGAGCGGGGATATCCCGCCGCCACACTCGAGCGGATG
CGGGCCACTTTCGGCCTGGAAATGGAGCAGACCCAGCTTTCTTGTACAAAGTGGTGG
TACCTCGAAGTTCCTATTCTCTACTTAGTATAGGAACTTCCATCCTTGGAGCTCCTTC
AGGAGGCGGTGCTACTGCTGGCGCTGGTGGAGCCGGTGGACCTGCGGGGTTAATTT
ACCCATACGATGTTCCTGACTATGCGGGCTATCCCTATGACGTCCCGGACTATGCAG
GATCCTATCCATATGACGTTCCAGATTACGCTTGACGCTAGCACTAAGCGTC

UAS MYB (2,102 bases)

GGGAATTGGGAATTCGTTAACAGATCTGCTACCGGTATACAAGTTTGTACAAAAAA
GCAGGCTAAAAAGCAGGCTTCAACATGGCAAGTGCGAGCACTGAAAACGGCGAGG
AGCTGATGAATTACGGCAGCAATTCGGATTCGGAGGAGTCCGAGTACTCGGAGAAC
GAGGATACCCAAGTTTGCGACAAGGATTCACAACAGAACAGCAATGCCGATTCGGG
CTATCCACTGGACTCGCCCGAACTACAGGACTCCAAGACGACGGGGCAAAAGGGAC
AGAACAAAAGCGGTAAAACTAGCATCGGTGCAGTGCATCCGAACTATGGATTCGGC
AAACGCTGGTCCAAGTCCGAGGATGTGTTGCTCAAGCAGCTGGTGGAGACGCACGG
TGAGAACTGGGAGATTATCGGACCGCATTTCAAGGATCGCCTGGAACAGCAAGTGC
AGCAGCGTTGGGCCAAAGTCCTCAATCCGGAGCTAATCAAGGGCCCGTGGACGCGG
GACGAGGACGACATGGTTATTAAGCTGGTGCGCAACTTCGGGCCCAAGAAATGGAC
ACTAATTGCACGATATCTGAACGGACGAATTGGGAAACAGTGCCGCGAGAGATGGC
ACAACCACCTCAATCCGAATATCAAGAAAACGGCCTGGACCGAAAAGGAGGACGA
GATTATCTACCAGGCTCACTTGGAGCTGGGCAACCAGTGGGCAAAGATTGCCAAAC
GTCTGCCCGGACGCACCGATAACGCCATTAAAAACCATTGGAATTCAACAATGCGT
CGCAAATATGACGTCGAACGCAGATCGGTTAATGCATCTGGTAGCGATTTGAAGAG
TTCGCGAACCCATCTCATCACGCTGATCAAGTCCGGTGGCATCAGCAAGTGTATGAA
TAATATGCAACATAACAAGGAATCTGGTGGTGAGGCAGTTAATAAATCCGAAAACG
CCGATGGCGCTTCGGTTACTGCCGTGAAAGGTGGTGATCTGGCACAGGAATCACAA
GACGATCATCAAAAGGGATCCAATCTGGCACACCTCAGTATGCAGCATCTCATTAAG

TTGACCATGCCGCGCCAAACACCGATCATATTGAAGCGTACACGCAAACACATTCCC
GAAACTCACCATCAGGCAGGATGTTCATCAAGTGAGACATTCAACCAGGAAGAAGC
CGCCGGAAACGCCAGGTCGCGACCACCGAGTTCGCCCGTCATATCGCCCATTAAGTC
GCTACCCTTTTCACCGAGTCACTTCCTCAAGTCGCCTTGCCTGACCACCTTTGAAGAT
ATGGATCTGCGGGCTAGCACGCCGGTAACCAAGGTCTATAATCGCGTTGGAATGGA
GATCAAAAAGGAAATGGAGACATCGTCGATAGAAACTCCACACAAGAGTCAACTTG
GACCACGAACACCAACGCCCTTCAAAAAGGCACTTGCTGCCATCGGCAAGAAGAGG
GACGGTCGTCGATACGAGCCCTCCAGCCCATCCAGTCTGGTGGAGGATCTGGCCGA
AATCATCCATGAAGAGCATCTGAGCAACTCGCTGACCGCAAACAATTCCAAAATGA
TGGGCGCCGCCGATCAGAACTCAACGCTAAGCACCGAATACAATGCTCAGTCGCCG
CCGCATATGAAACGTGCCAGGAAGTCATTGCTCTCAACTTGGAGCTCCAATCATCCA
TACAACGCTGGCTCCGCCAAGAGGATTCAACCCTTCGAAACCGAGACGCCCAGCAA
GTTTCTCACATCGCCGGGCGATATTTTGAAGGACACGCTGTGCAGCGAACAGGACCT
GCCGTTCGACGAGGGCAGAAAGGAGAATCGACCATTCCATAATCGCAGAATTAACA
AGTACCGCGGTGGCTTGACCTACGATCATGTCATTGATCCCAAGTGGGCACGCGTCG
CTTGTGGCAAGTCCAGAGATCAAATGTTTATGGAGGAGCAGGCTTATGCGTGCCTCA
AAAATCTGTCCTGTATTTCGCGCTCCCTGAACTTCGAGAAACAAAAGTGTTTGGTGA
ATTCGTTCGACCGCTTTGGTTCGCTCTAAACCCAGCTTTCTTGTACAAAGTGGTGACG
TAAGCTAGCACTAAGCGTC
